# Cost-effectiveness of pharmaceutical strategies to prevent respiratory syncytial virus disease in young children: a decision-support model for use in low-income and middle-income countries

**DOI:** 10.1186/s12916-023-02827-5

**Published:** 2023-04-11

**Authors:** Sarwat Mahmud, Ranju Baral, Colin Sanderson, Clint Pecenka, Mark Jit, You Li, Andrew Clark

**Affiliations:** 1 Department of Health Services Research and Policy, Faculty of Public Health and Policy, London School of Hygiene and Tropical Medicine, London, UK; 2 PATH, Seattle, WA USA; 3 Department of Infectious Disease Epidemiology, London School of Hygiene and Tropical Medicine, London, UK; 4 Modelling and Economics Unit, Public Health England, London, UK; 5 Department of Epidemiology, School of Public Health, Nanjing Medical University, Nanjing, China

**Keywords:** RSV, LMICs, Maternal vaccine, Monoclonal antibody, Economic evaluation

## Abstract

**Background:**

Respiratory syncytial virus (RSV) is a leading cause of respiratory disease in young children. A number of mathematical models have been used to assess the cost-effectiveness of RSV prevention strategies, but these have not been designed for ease of use by multidisciplinary teams working in low-income and middle-income countries (LMICs).

**Methods:**

We describe the UNIVAC decision-support model (a proportionate outcomes static cohort model) and its approach to exploring the potential cost-effectiveness of two RSV prevention strategies: a single-dose maternal vaccine and a single-dose long-lasting monoclonal antibody (mAb) for infants. We identified model input parameters for 133 LMICs using evidence from the literature and selected national datasets. We calculated the potential cost-effectiveness of each RSV prevention strategy (compared to nothing and to each other) over the lifetimes of all children born in the year 2025 and compared our results to a separate model published by PATH. We ran sensitivity and scenario analyses to identify the inputs with the largest influence on the cost-effectiveness results.

**Results:**

Our illustrative results assuming base case input assumptions for maternal vaccination ($3.50 per dose, 69% efficacy, 6 months protection) and infant mAb ($3.50 per dose, 77% efficacy, 5 months protection) showed that both interventions were cost-saving compared to status quo in around one-third of 133 LMICs, and had a cost per DALY averted below 0.5 times the national GDP per capita in the remaining LMICs. UNIVAC generated similar results to a separate model published by PATH. Cost-effectiveness results were most sensitive to changes in the price, efficacy and duration of protection of each strategy, and the rate (and cost) of RSV hospital admissions.

**Conclusions:**

Forthcoming RSV interventions (maternal vaccines and infant mAbs) are worth serious consideration in LMICs, but there is a good deal of uncertainty around several influential inputs, including intervention price, efficacy, and duration of protection. The UNIVAC decision-support model provides a framework for country teams to build consensus on data inputs, explore scenarios, and strengthen the local ownership and policy-relevance of results.

**Supplementary Information:**

The online version contains supplementary material available at 10.1186/s12916-023-02827-5.

## Background

Respiratory syncytial virus (RSV) is a leading cause of respiratory disease in young children, causing over 100,000 RSV-ALRI (RSV-associated acute lower respiratory infection) deaths in low-income and middle-income countries (LMICs) in the year 2019 [1].

Only one prophylactic RSV intervention is currently available: an injectable monoclonal antibody (mAb), palivizumab, [2] (brand name S*ynagis* – AstraZeneca, Cambridge, UK) [3] for use in infants who are particularly vulnerable to RSV disease, including those who are preterm, immunocompromised, or living with pulmonary or congenital heart disease [4]. Palivizumab has been registered for use in over 60 countries worldwide, but its high cost (> US$ 4000 for a complete course of up to five injections [5]) makes it unaffordable in most LMICs [6]. Even in high-income countries, it is generally only cost-effective when restricted in use to months when RSV is prevalent.

An array of new RSV interventions is likely to become available in the future, including maternal vaccines, and lower-cost mAbs and vaccines for infants [7–9]. A number of mathematical models of RSV have been used to assess the potential impact and/or cost-effectiveness of these strategies [10], but these have not been designed for ease of use at country level in LMICs, i.e. by multidisciplinary country teams working on behalf of vaccine technical advisory committees [11]. The UNIVAC (universal vaccine) decision-support model [12], and earlier versions of this model [13, 14], have been used by country teams in over 30 LMICs to evaluate the impact and cost-effectiveness of several other vaccines [15]. UNIVAC has recently been adapted to allow the evaluation of two RSV prevention strategies: maternal vaccines and infant mAbs.

This paper presents an overview of the RSV component of UNIVAC, synthesises RSV model input parameters for 133 LMICs, compares the cost-effectiveness results of UNIVAC to a separate model published by PATH [16], and identifies the inputs with the largest influence on the cost-effectiveness results. The paper is not intended as a conclusive evaluation of the cost-effectiveness of RSV interventions, or comparisons between them, given the remaining uncertainties about interventions still in development. The paper aims to provide a reference document for multidisciplinary country teams in LMICs that may be interested in using UNIVAC to explore the potential cost-effectiveness of strategies to prevent RSV disease in children aged < 5 years.

## Methods

### UNIVAC decision-support model

UNIVAC is a decision-support model developed in Excel and Visual Basic for Applications. It is a proportionate outcomes static cohort model [17] that can be used to explore potential costs (intervention programme costs, healthcare cost saved by the intervention) and direct health effects (reduction in cases, visits, hospital admissions, deaths, DALYs [disability adjusted life years]), for different RSV prevention strategies, over the lifetimes of target birth cohorts. As a static model, it takes no account of indirect effects, such as herd immunity. The importance of these effects is yet to be established for maternal RSV vaccines and infant mAbs.

The primary outcome measure in UNIVAC is the cost per DALY averted. The comparator is a scenario assuming no pharmaceutical RSV intervention strategy. Healthcare costs saved by the RSV prevention strategy are subtracted from intervention programme costs to estimate incremental net costs. Incremental net costs are then divided by the number of DALYs averted to calculate the cost per DALY averted. Country teams should create one UNIVAC file per RSV prevention strategy and compare results in a separate summary table. When comparing alternative RSV interventions, strategies that are dominated (both more expensive and less effective than others) should be removed and the cost per DALY averted should be recalculated using a new comparator, i.e. comparing each strategy to the next least costly alternative.

Users of the model should specify the types of RSV disease (e.g. non-severe and severe RSV disease), intervention strategy (e.g. maternal vaccine, infant mAb), duration of the intervention period (e.g. 2025 or 2025–2034), the scope of costs to be included (e.g. government and/or societal cost perspective), currency/year of cost inputs (e.g. 2022 USD), the rate at which future costs and health effects are discounted (e.g. 0% and/or 3%). Adherence to WHO guidelines for economic evaluations of immunisation programmes is strongly recommended; a standard checklist table should be used to report methods and allow others to appraise the quality of the evaluation [18].

**Fig. 1:**
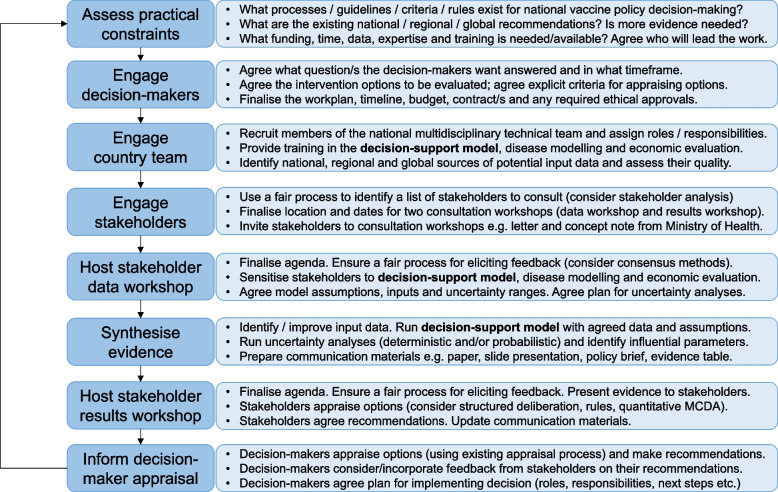
Integration of a decision-support model into a country-led vaccine decision-making process

UNIVAC has been designed for ease of use and ownership at the country level. This requires meaningful engagement with stakeholders and close alignment with existing processes for health technology appraisal and decision-making in the country concerned (Fig. 1).

### RSV disease categories and outcomes

**Fig. 2:**
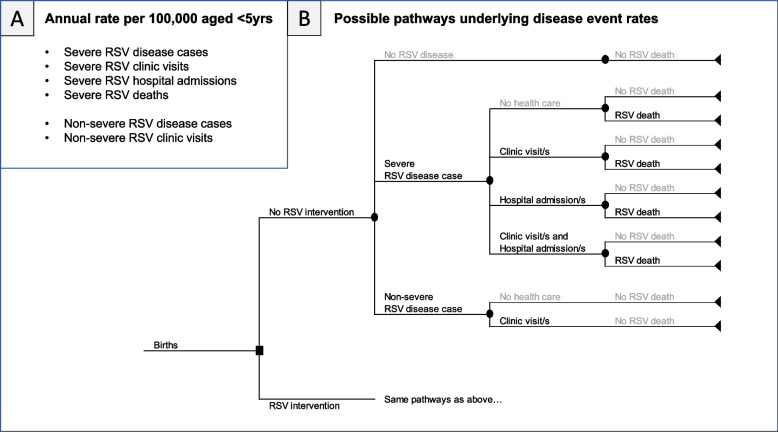
Rates of RSV disease events in the recommended setup of UNIVAC (**A**) and possible pathways underlying these disease event rates (**B**). Caption: While there are many pathways to RSV death (and therefore different probabilities of dying for different treatment pathways), for simplicity only one rate of RSV mortality is required by the model. Similarly, while many healthcare utilisation pathways (clinic visits and/or hospital admissions) are possible, for simplicity all this activity is captured within a single rate of RSV hospital admissions, and two rates of RSV clinic visits (one for severe RSV and one for non-severe RSV). The model does not include a separate category for emergency room (ER) visits, and since the model will assume the same efficacy and treatment costs for all outcomes that are grouped together, country teams should clarify whether ER visits are included within the estimated rate of clinic visits or hospital admissions. Estimates of the average treatment cost per patient should also be adjusted appropriately to account for the contribution and cost of ER visits

Bronchiolitis (blockages in the bronchioles of the lungs) and pneumonia (fluid in the air sacks or alveoli of the lungs) are the most common features of severe RSV-ALRI disease [19]. In the recommended setup of UNIVAC, the model requires rates of severe RSV disease outcomes (cases, clinic visits, hospital admissions and deaths) and non-severe RSV disease outcomes (cases, clinic visits). These are each entered as rates per 100,000 per year in children aged < 5 years. We assume a range of possible pathways can result in these disease outcomes (Fig. 2). Cases are defined as all symptomatic RSV disease cases that occur in the community, irrespective of whether they received healthcare or not. Severe cases are those that require hospital admission [20]. Clinic visits are RSV disease cases treated in an outpatient setting, e.g. health centre or hospital outpatient. Hospital admissions are RSV disease cases admitted to a hospital ward. UNIVAC includes options to adapt the RSV disease categories and their outcomes (cases, clinic visits, admissions, and deaths) if required. Country teams should try to align these as closely as possible with available disease burden estimates and efficacy end points from clinical trials. The Additional file 1: Table S1, [1, 20–33] includes a list of alternative disease categories and definitions that could be considered.

### Cost of RSV healthcare

UNIVAC calculates total healthcare costs by multiplying the number of clinic visits and hospital admissions by the respective average cost per visit or hospital admission. Average costs of visits/admissions per disease episode can be calculated outside the model by calculating the cost per visit or admission for different types of healthcare providers and the share of total visits/admissions provided by each. It is assumed that the mix of providers does not change after vaccination.

Country teams should decide on the healthcare costs to be taken into account. A government (public-sector payer) perspective will typically include bed-day costs (buildings, nurse salaries, etc.) and disease-specific costs (e.g. drugs, tests) incurred by providers in the public health sector. A societal perspective typically includes all costs included in the government perspective plus direct out-of-pocket costs borne by patients and their families (e.g. travel, drugs, and fees for using private healthcare). Country teams should also decide whether to include indirect costs associated with the lost wages of patients and family members.

### RSV interventions and schedule options

UNIVAC includes the following two RSV prevention strategies: (i) maternal vaccine – single injection co-administered with antenatal care (ANC) at 24–36 weeks gestation and (ii) infant mAb – single injection co-administered with an existing birth dose vaccine, e.g. Bacillus Calmette-Guérin (BCG). Specific product brand names are not used in the model because of the large number of products currently in the pipeline [7, 8], including maternal vaccines, such as RSVpreF [34], and infant mAbs, such as Nirsevimab [35]. The model has the option to include serious adverse events, but recent clinical trials have reported similar rates of adverse rates in the vaccine and placebo groups, so this is not currently recommended. Country teams should adapt the input parameters to reflect the particular product/s they wish to evaluate.

### Cost of RSV prevention strategies

The cost of RSV prevention strategies includes a number of parameters, including the price and wastage of doses, syringes and safety boxes. International handling fees and international transportation fees are also applied, together with the incremental cost to the health system of introducing the new RSV prevention strategy. Full calculations are provided in Additional file 1 (page 5).

Country teams should also decide on what intervention-related costs to take into account, ensuring consistency with the perspective/s used for healthcare costs. Typically a government (public-sector payer) perspective will exclude any contributions from donors (e.g. Gavi) and the level of contribution can be altered for each target birth cohort. In contrast, a societal perspective will incorporate the full price of the vaccine or mAb, irrespective of who pays. Since RSV interventions are likely to be co-administered with existing interventions (e.g. ANC or BCG) it may be reasonable to assume there are minimal incremental costs to patients and their families.

In order to estimate the potential cost of a maternal vaccination strategy, it is necessary to estimate the number of pregnant women eligible. This was done by adding the number of live births to the number of stillbirths in a given country and calendar year. Miscarriages and abortions were not factored into the calculation as the vast majority tend to occur in early pregnancy, not at 24–36 weeks of gestation, when the maternal vaccine would typically be administered. For each country, the number of live births was taken from standard UN Population Division projections by calendar year [36]. The number of stillbirths was estimated for the years 2000–2015 and adjusted to account for the expected rate of change over time [37], consistent with the approach used by Baral et al. [38].

### Calculating the impact of RSV prevention strategies

For a given week *w* of age in the birth cohort targeted by the RSV intervention, the number of disease events (cases, clinic visits, hospital admissions, deaths) was calculated as:

$$\mathrm{PY}\;\ast\;\mathrm D\;\ast\;\mathrm{Aw}\; \ast(1-(\mathrm C1\mathrm w\;\ast\;\mathrm E1\mathrm w))$$

where PY is the number of person-years lived between birth and age 5.0 years in birth cohort of interest (derived from UN Population Division interpolated single age/year population estimates [36]); D is the disease event rate (per 100,000 per year) among children < 5 years of age before the introduction of a maternal vaccine or infant mAb; Aw is the proportion of disease events < 5 years of age in week w of age; C1w is the intervention coverage estimated in week w of age (after considering realistic delays in dose administration/timeliness), and E1w is the efficacy estimated in week w of age, adjusted for any waning from the time of dose administration.

The model assumes that efficacy (and waning if applicable) starts at birth for all children born to vaccinated women. For simplicity, the vaccine coverage of pregnant mothers in a given calendar year is applied to newborns in the same calendar year. For infant mAbs, the user can either assume that the efficacy/waning starts at a specified age for all infants included in the programme (e.g. birth, 1 month) or assume it is aligned with the timeliness and coverage of other birth dose vaccines by week of age, e.g. BCG, hepatitis B or polio birth dose. Efficacy can be fixed for a period of time (e.g. 6 months) or assumed to decline gradually over time (by exploring different mean and shape parameters of a cumulative gamma distribution). Maternal vaccine efficacy may also need to be adjusted to better represent when the vaccine is administered to mothers in the gestational period.

The default setup of the model assumes a constant year-round incidence of RSV. However, many LMICs have clear seasonal RSV epidemics [39, 40], and some countries may wish to explore the cost-effectiveness of targeting only those at high risk. The proportion of the cohort that is high risk should be defined by the country team, e.g. the proportion of pregnant women due to give birth during months A, B and C or the proportion of the cohort aged < X months during months A, B and C. The target group could be further refined by identifying the proportion who are at particularly high risk, i.e. those who are preterm, immunocompromised, or living with pulmonary or congenital heart disease [4]. When exploring strategies targeted to high-risk groups, country teams will need to calculate the expected number of RSV events occurring in each month of the year and the expected intervention coverage in the corresponding months of the year.

### Options for uncertainty analysis

UNIVAC includes a facility for customising and running a range of deterministic scenarios agreed by the country team. In accordance with WHO guidelines, country teams are recommended to run at least one scenario that is most favourable to the intervention, and one that is least favourable [18]. UNIVAC also includes options to run probabilistic uncertainty analysis. If data permits, then more advanced users can specify custom distributions for each input parameter (e.g. log-normal, gamma) and quantify the extent to which different parameters are correlated. Alternatively, a less rigorous option involves specifying the minimum, maximum and most likely values for each parameter, and assuming PERT-Beta distributions [41]. As a further simplification, groups of parameters that are assumed to be highly correlated (e.g. rates of severe disease cases and rates of hospital admissions) can be assigned the same random number drawn per run. The limitations of these less rigorous options should be clearly communicated. The number of probabilistic runs specified by the country team (e.g. 1000) should be large enough to ensure stability in the 2.5th and 97.5th percentiles (95% uncertainty interval) of the primary outcome measure (cost per DALY averted). Standard charts include (a) a cost-effectiveness plane with probabilistic runs represented as clouds of uncertainty around the central estimates of incremental net costs and numbers of DALYs averted and (b) a cost-effectiveness acceptability curve (CEAC) showing the probability that each RSV strategy will be cost-effective, i.e. the proportion of probabilistic runs with cost-effectiveness ratios below different willingness-to-pay (WTP) thresholds.

### Synthesis of model input parameters and model comparison exercise

We identified a common set of input parameters for each country (*n* = 133 LMICs) based on a review of the scientific literature and new analyses of national datasets (Tables 1 and 2). Additional file 1 (pages 6–33, [1, 16, 22, 28, 34, 42–58]) provides further details and includes all inputs and uncertainty ranges (Tables S2–S8, [1, 16, 43, 44, 50] suggested for each country.

**Table 1:** Disease burden and healthcare costs used for model comparison exercise

Input parameter	Value	Source
**RSV disease event rates per 100,000 per year (< 5yrs)**		
RSV non-severe cases		
* Low income*	4450	Li 2022 [1]
*Lower middle income*	3740	Li 2022 [1]
*Upper middle income*	4830	Li 2022 [1]
RSV severe cases		
*Low income*	480	Li 2022 [1]
*Lower middle income*	1400	Li 2022 [1]
*Upper middle income*	690	Assume 1/8 of all RSV-ALRI cases in Li 2022 [1]
RSV clinic visits		
*Low income*	2642	RSV cases × 53.6% treatment for pneumonia [59]
*Lower middle income*	3395	RSV cases × 66.0% treatment for pneumonia [59]
*Upper middle income*	3949	RSV cases × 71.5% treatment for pneumonia [59]
RSV hospital admissions		
*Low income*	350	Li 2022 [1]
*Lower middle income*	620	Li 2022 [1]
*Upper middle income*	620	Li 2022 [1]
RSV deaths		
*Low income*	29.86	Li 2022 [1]
*Lower middle income*	19.76	Li 2022 [1]
*Upper middle income*	4.73	Li 2022 [1]
Cumulative percentage of RSV severe cases < 5 years, by age *		
1 m	3.91%	A Burr distribution was fitted to RSV hospital admission data from two unpublished datasets (Argentina and Vietnam) and four studies (Kenya [45], Mozambique [46], Pakistan [47], and South Africa [48]) identified in Li, 2022 [1]
3 m	35.60%	
6 m	59.53%	
1 yr	77.21%	
2yrs	89.48%	
5yrs	100.00%	
Cumulative percentage of RSV non-severe cases < 5 years, by age**		
1 m	0.29%	The ratio of non-severe to severe RSV case incidence from Li, 2022 [1] was applied in broad age bands (< 3 m, 3–5 m, 6–11 m, 12–59 m) to the fitted Burr age distribution for severe RSV cases. An updated Burr age distribution was then fitted to non-severe RSV cases
3 m	10.65%	
6 m	37.90%	
1 yr	65.85%	
2yrs	84.91%	
5yrs	100.00%	
**DALY weights**		
RSV non-severe cases	0.051	GBD, 2019 [49]
RSV severe cases	0.133	GBD, 2019 [49]
**Duration of illness (days)**		
RSV non-severe cases	5	Hall et al. [60] − / + 2 days
RSV severe cases	10	Hall et al. [60] − / + 2 days
**Healthcare costs (US$)**		
RSV clinic visits	62.24	Cost of managing pneumonia, Zhang et al. [51]
RSV hospital admissions	368.42	Cost of managing pneumonia, Zhang et al. [51]

Abbreviations: *m* months, *yrs* years

^*^ Also assumed for RSV hospital admissions, RSV deaths, and RSV clinic visits among severe RSV cases. A Burr distribution (shape 1 = 2.9, shape 2 = 0.2, scale = 7.0) was used to calculate the age distribution by week of age (Additional file 1: Fig. S1)

^**^ Also assumed for RSV clinic visits among non-severe cases. A Burr distribution (shape 1 = 3.3, shape 2 = 0.2, scale = 15.1) was used to calculate the age distribution by week of age (Additional file 1: Fig. S2)

**Table 2:** Programme impact and cost assumptions used for RSV prevention strategies in the model comparison exercise

Input parameter	Maternal vaccine	Source	Infant mAb	Source
**Impact of RSV prevention strategy**				
Programme coverage*	ANC (proxy)	Baral et al. [16]	BCG (proxy)	WUENIC [50]
Efficacy (RSV severe cases)	69.4%	Pfizer [34]	77.3%	Sanofi [52]
Efficacy (RSV non-severe cases)	51.3%	Pfizer [34]	74.5%	Sanofi [52]
Duration of protection (fixed for *n* months)	6	Pfizer [34]	5	Sanofi [52]
**Percentage wastage**				
Doses	5.00%	Assumption	5.00%	Assumption
Syringes	5.00%	Assumption	5.00%	Assumption
Safety boxes	5.00%	Assumption	5.00%	Assumption
**Price per dose (US$)**				
GAVI countries	US$ 3.50	Assumption	US$ 3.50	Assumption
Non-GAVI countries	US$ 7.00	Assumption	US$ 7.00	Assumption
**International handling (% of dose price)**				
GAVI countries	1.40%	UNICEF [58]	1.40%	UNICEF [58]
Non-GAVI countries	3.50%	UNICEF [58]	3.50%	UNICEF [58]
**International transportation (% of dose price)**				
All countries	6.00%	Debellut [61]	6.00%	Debellut [61]
**Other injection supply costs**				
Syringe price per dose (US$)	US$ 0.0278	UNICEF [54]	US$ 0.0278	UNICEF [54]
Safety box price per dose (US$)	US$ 0.0121	UNICEF [54]	US$ 0.0121	UNICEF [54]
**Incremental health system cost per dose (US$)**				
Low-income countries	US$ 0.74	ICAN [62]	US$ 0.74	ICAN [62]
Middle-income countries	US$ 2.02	ICAN [62]	US$ 2.02	ICAN [62]

^*^Additional file 1: Table S9 [16, 50]

We compared results from UNIVAC (developed by researchers at the London School of Hygiene & Tropical Medicine) to those from a separate proportionate outcome static cohort model developed in Stata by PATH [16], using established principles for multi-model comparisons [63]. Each model evaluated the cost-effectiveness of two interventions (maternal vaccine, infant mAb) over the lifetimes of a single birth cohort (2025) in 133 LMICs. Each RSV prevention strategy was compared to nothing (a scenario without any pharmaceutical RSV intervention) and to each other. We used a discount rate of 3% for future costs and health effects, a currency year of US$ 2022 [57] (January) and a societal cost perspective. The methods of this economic evaluation are summarised in Additional file 1: Table S10, [18] using the standard WHO checklist for appraisal of economic evaluations of immunisation programmes [18].

In the PATH model, disease event rates are entered in monthly age intervals, but assumed to be the same within broad age bands (< 3 m, 3–5 m, 6–11 m, 12–59 m). In UNIVAC, a single disease event rate (< 5 years) is entered, and a granular age distribution (260 weeks of age < 5 years) is applied post hoc. The age-specific rates used in the PATH model were aligned with the input data used in UNIVAC by assuming the same overall disease event rates aged < 5 years (reference year = 2019), and age distributions consistent with the parametric (Burr) age distributions fitted to data from 6 countries (Additional file 1, Figs. S1–S2). The inputs used to calculate the total cost per dose of each RSV prevention strategy were also aligned.

A standardised output spreadsheet was used to compare modelled estimates of the cost per DALY averted, and other outcome measures, across 133 LMICs. For each outcome measure, we calculated the absolute percentage difference between the two model results for all countries combined. We also compared the percentage of 133 LMICs that would be willing to pay for each intervention, at different WTP thresholds below 1 times the national GDP per capita [64].

### Identifying influential input parameters

We ran scenario and sensitivity analyses to identify the parameters that had the most influence on the UNIVAC cost-effectiveness results. For both RSV prevention strategies (maternal vaccine, infant mAb) we compared the percentage of 133 LMICs that would be willing to pay for each intervention, at different WTP thresholds, for the base case and eight alternative what-if scenarios (Additional file 1: Table S11). We also generated illustrative sensitivity analyses for two countries (one low-income country and one middle-income country). In this analysis we varied each parameter in turn by − / + 10%, i.e. by multiplying the central estimate by 0.9 and 1.1, respectively, and noting the effect of this change on the cost per DALY averted.

## Results

**Fig. 3:**
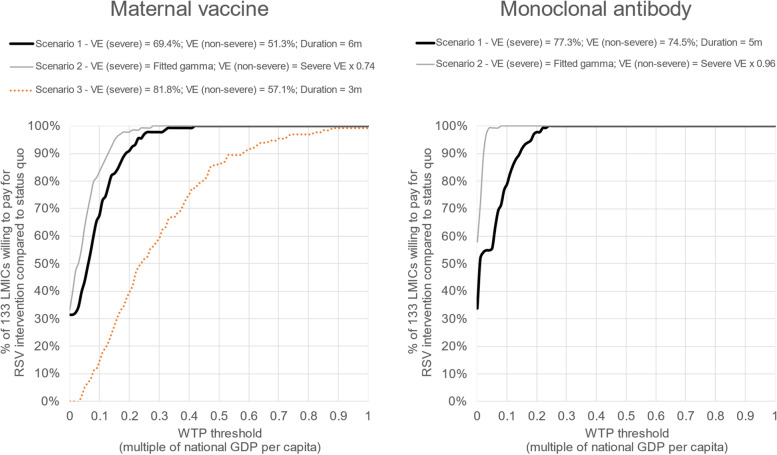
Percentage of 133 LMICs willing to pay for RSV prevention strategies (compared to no pharmaceutical intervention) at different willingness-to-pay thresholds: base case and alternative efficacy scenarios. Caption: The thick black lines show the base case (scenario 1) assumptions. For maternal vaccination, this assumes efficacy of 69.4% (severe RSV disease) and 51.3% (non-severe RSV disease) for a 6-month period and zero protection thereafter. For mAb this assumes efficacy of 77.3% (severe RSV disease) and 74.5% (non-severe RSV disease) for a 5-month period, and zero protection thereafter. The grey lines show the scenario 2 assumptions. For this scenario, we used previously described methods [53] to fit estimates of instantaneous efficacy (iE) that were consistent with the reported cumulative efficacy (cE) at 3 and 6 months of follow-up (see Additional file 1, page 32, and Fig. S3, for more details). Finally, we applied one additional scenario (scenario 3) for maternal vaccination with efficacy of 81.8% (severe RSV disease) and 57.1% (non-severe RSV disease) for a 3-month period, and zero protection thereafter

Our illustrative results assuming base case input assumptions for maternal vaccination ($3.50 per dose, 69% efficacy, 6 months protection) and infant mAb ($3.50 per dose, 77% efficacy, 5 months protection) showed that both interventions had very similar cost-effectiveness compared to no pharmaceutical RSV intervention (Fig. 3). In our base case scenario, the national cost per DALY averted for maternal vaccination was less than 0.4 times the national GDP per capita in all 133 LMICs. Cost-effectiveness ratios were slightly more favourable for infant mAb, with all national cost per DALY averted estimates below 0.25 times the national GDP per capita.

With our base case assumptions, both interventions were cost-saving compared to the status quo in around one-third of 133 LMICs (42 countries for maternal vaccine and 45 for mAb). Infant mAb prevented more DALYs in 92% (122/133) of LMICs and had a lower net cost than maternal vaccination in 86% (115/133) of LMICs. Infant mAb was dominant (had both greater impact and lower net costs than maternal vaccination) in 79% (105/133) of LMICs.

Results were very sensitive to the choice of efficacy assumptions. A scenario assuming declining vaccine protection over time improved the cost-effectiveness ratios for both interventions. The cost-effectiveness of maternal vaccination was less favourable when we assumed a shorter duration of protection (3 months) despite incorporating higher efficacy (82%) in this period (Fig. 3). For both maternal vaccines and infant mAbs, doubling the dose price had an important influence on the results (Additional file 1: Fig. S4–S5). However, > 80% of LMICs would still be willing to pay for infant mAb (and > 70% would be willing to pay for maternal vaccination) at a WTP threshold set at 0.5 times the national GDP per capita.

Illustrative sensitivity analyses in two countries showed that cost-effectiveness results were very sensitive to changes in the price, efficacy, and duration of protection of RSV prevention strategies, and the rate (and cost) of hospital admissions (Additional file 1: Fig. S6–S7).

UNIVAC results were consistent with the results of a separate model published by PATH (Additional file 1: Tables S12–S13, Fig. S8) with UNIVAC results slightly more likely to be cost-effective for mAb. The high number of cost-saving results made it difficult to compare national or aggregate cost-effective ratios in a meaningful way. However, all intermediate outcomes were similar with and without each intervention, as was the percentage of LMICs willing to pay for each intervention at different WTP thresholds.

## Discussion

This paper provides a reference document for multidisciplinary country teams that may be interested in using the UNIVAC decision-support model to explore the potential cost-effectiveness of RSV prevention strategies in LMICs. To support future analyses we have documented the methods of the modelling approach, synthesised RSV model input parameters for 133 LMICs, indicated which parameters are likely to have the greatest influence on the cost-effectiveness results, and found good agreement between UNIVAC and a separate model published by PATH.

We find that an infant mAb or maternal vaccine priced at $3.50 per dose has the potential to either be cost-saving or warrant serious consideration in all LMICs. While intervention prices remain uncertain, the Bill and Melinda Gates Foundation (BMGF) has made substantial investments in maternal immunisation, including for RSV [65]. These investments suggest that at least some RSV interventions may be priced accessibly for LMICs. We also acknowledge that prices for mAbs may substantially exceed those for maternal vaccines, but we have no basis to accurately differentiate these prices in this analysis.

In our analysis, we compared both interventions separately to status quo (no pharmaceutical RSV intervention) rather than comparing them directly to one another. This is appropriate because subtle changes in the efficacy/waning assumptions used for either intervention could have easily changed their rank order. Also, in some populations, it may only be feasible to use one of the available options to protect infants. Comparing each option to the status quo is therefore appropriate for our illustrative results. As more becomes known about each intervention (dose price, efficacy, duration of protection) calculating the incremental cost-effectiveness of one option over another would be appropriate if both can be used to protect the same infants.

One potentially important limitation of our illustrative ‘multi-country’ estimates is that we have assumed ‘year-round’ incidence of RSV disease. This will overestimate cost-effectiveness in settings with seasonal incidence because RSV interventions have short-lived protection and may either be given too early to be effective when needed or given to those with no risk of acquiring RSV. However, in these circumstances it may be feasible to target RSV interventions to those at greatest risk, requiring fewer doses per unit of health benefit. This will require careful consideration of the incremental health system cost and intervention coverage associated with targeted RSV interventions. For example, it may be unrealistic to assume BCG coverage levels can be achieved if the strategy involves catchup campaigns for older infants.

There is a good deal of uncertainty around several influential inputs, including intervention price, efficacy, and duration of protection. Running the model for different subgroups (e.g. on the basis of prematurity and/or seasonality) could also have an important influence on our results. Given these gaps in the currently available evidence, our illustrative results should not be used to prioritise one intervention over the other. Our estimates should also not be considered a replacement for more thorough economic evaluations at country level. Such studies should include engagement with stakeholders to build consensus on the most appropriate input data and scenarios for uncertainty analysis.

Social distancing and lockdown measures to control the COVID-19 pandemic have dramatically reduced the global burden of respiratory pathogens, including RSV, but as social mixing increases, RSV incidence is expected to return to pre-pandemic levels (and indeed potentially to exceed them for a few years due to greater than normal accumulation of susceptibility during the pandemic) [66]. Our results show that forthcoming RSV interventions (maternal vaccination and infant mAbs) have the potential to cost less than 0.5 times the national GDP per capita to avert each DALY, and are therefore worth serious consideration. This may be close to the health opportunity cost of spending at the healthcare budget margin in many LMICs [67] and seems close to the revealed WTP of many LMICs for vaccines [17, 68]. The precise threshold should ideally reflect the context and circumstances of each country [69] but this may be challenging to establish in many LMICs. In addition, WTP thresholds should not be the only criteria used for appraising RSV interventions at country level [70].

UNIVAC generated similar results to a separate model published by PATH. Minor differences were due to subtle differences in the methods used to estimate disease burden and intervention impact. UNIVAC modelled RSV disease events expected to occur in the first five years of life for all children born in 2025, whereas the PATH model estimated RSV disease events for the 2025 calendar year. The PATH model applied age-specific RSV disease rates (reference year 2019) directly to age-specific populations for the year 2025 to generate numbers of RSV disease cases in broad age bands. In contrast, UNIVAC applied the overall RSV disease event rate < 5 years (reference year 2019) to the number of life-years at risk between birth and age 5.0 years (derived for all those born in the year 2025) and assigned RSV disease events into weeks of age (0–260 weeks) post hoc. PATH also used monthly age bands below 6 months to calculate the expected impact of RSV prevention strategies, whereas UNIVAC calculated the expected impact in each week of age. This led to subtle differences between the modelled impact of infant mAb because the duration of protection (5 months) did not align exactly with the age bands used to align disease burden inputs for the two models (< 6 months). The high number of cost-saving results made it difficult to compare national or aggregate cost-effective ratios in a meaningful way, but both models predicted a similar percentage of LMICs that would be willing to pay for each intervention at different WTP thresholds. Alignment between UNIVAC and the PATH model is reassuring, but unsurprising given that both are proportionate outcome static cohort models. A separate static cohort model by Li et al. has also reported results consistent with the PATH model across 73 Gavi-eligible countries [71]. Models programmed in Stata or R could be made available online or integrated into a more user-friendly web application. However, one of the main advantages of UNIVAC is that it has been developed in Excel, software that is transparent and likely to be familiar to multidisciplinary teams (including non-modellers) working on behalf of vaccine decision-makers in LMICs.

Unlike dynamic models, static models are unable to capture indirect effects, such as herd immunity. For this reason, static cohort models are likely to underestimate the benefit and cost-effectiveness of RSV prevention strategies. This limitation should be carefully communicated to decision-makers when sharing results. However, it is currently unclear whether or to what extent RSV interventions will reduce infectiousness or prevent the acquisition of RSV infection, and thus whether herd immunity is an issue here. Dynamic models also require data that is often unreported or highly uncertain in LMICs. For example, a recent review of mathematical models of RSV in LMICs identified the potential importance of accounting for social contact rates and immunity from prior infection [10]. Our analysis was restricted to maternal vaccines and infant mAbs, but infant vaccines administered later in infancy could help to reduce overall transmission in the community if they provide some protection against infection and/or onward transmission. There are also several other aspects of the model structure to consider such as seasonality and individual-level heterogeneities in risk and coverage. Others have identified the potential importance of capturing interactions between other co-circulating respiratory pathogens and RSV [72], the favourable effect that RSV prevention strategies may have on reducing antibiotic prescribing in the context of antimicrobial resistance [73], and the effect that prophylactic interventions may have on the acquisition of natural immunity [74].

Our cost-effectiveness results were most sensitive to changes in the price, efficacy and duration of protection of each strategy, and the rate (and cost) of RSV hospital admissions. Other parameters were also influential, such as the mean age of severe RSV disease. For some of these inputs, it should be possible to strengthen model estimates by collecting new data or using data that are already in the public domain, e.g. the rate and costs of RSV hospital admissions and the mean age of severe RSV disease. Current estimates of RSV disease age distribution have been derived from a relatively small number of disease incidence studies and have reported age distributions in wide age bands [1]. A systematic review of RSV age distributions could help to improve the precision of modelled impact estimates, particularly if paired with similarly granular data on the coverage and timeliness of RSV interventions [75]. Similar exercises have recently been conducted for rotavirus disease [76] and intussusception [12] and these methods (systematic review, parametric curve fitting) could be readily applied to RSV.

## Conclusions

Forthcoming RSV interventions are worth serious consideration in LMICs, but there is a good deal of uncertainty around several influential inputs, including intervention price, efficacy, and duration of protection. Our experience of using decision-support models with country teams [77] has shown they have the potential to strengthen national capacity, help build consensus between stakeholders, and increase the local ownership and policy-relevance of results.

## Supplementary Information

**Additional file 1.** Additional details on methods, calculations, disease input parameters, results of the comparison exercise, sensitivity, and scenario analysis. **Table S1-S13** and  **Figure S1-S8**. **Table S1.** Definitions of disease categories. **TableS2.** Severe RSV disease, aged <5 years (per 100,000 children, per year). **Table S3.** Non-severe RSV, aged <5 years (per 100,000 children, per year). **Table S4.** Incidence of asthma in 2019, aged <5 years (per 100,000 children per year). **Table S5.** RSV mortality rate, aged <5 years (per 100,000 children per year). **Table S6.** Hospital admission rates, aged <5 years (per 100,000 children per year). **Table S7.** Incidence of clinic visits for severe RSV, aged 5 years (per 100,000 children, per year). **Table S8.** Incidence of clinic visits for non-severe RSV, aged 5 years (per 100,000 children, per year). **Figure S1.** Age distribution of severeRSV-ALRI cases in first year of life. **Figure S2.** Age distribution of non-severe and severe RSV-ALRI cases by age infirst year of life. **Table S9.** Estimates of maternal coverage (using ANC) as a proxy for maternal RSV vaccineand national immunization coverage of existing vaccines as a proxy for mAb. **Figure S3.** Efficacy scenarios used formaternal vaccination and monoclonal antibody. **Table S10.** WHO checklist for appraisal of economic evaluation andmodel comparison exercise. **Table S11.** Description of alternative scenarios. **FigureS4.** Percentage of 133 LMICs willing to pay for mAb RSV intervention. **Figure S5.** Percentage of 133 LMICswilling to pay for maternal vaccine RSV intervention. **Figure S6.** Percentage difference in cost per DALY averted in alow-income country, relative to baseline cost of US$3 for mAb and US$36, when each parameter is varied by +/-10%. **Figure S7.** Percentage difference in cost per DALY averted in a middle-income country, relative to baseline cost of US$733 for mAb and US$929 for maternal vaccine, when each parameter is varied by +/-10%. **Table S12.** Comparison of maternal vaccine estimates by UNIVAC model and PATH model for the 2025 birth cohort in 133 LMICs. **Table S13.** Comparison of monoclonal antibody estimates by UNIVAC model and PATH model for the 2025 birth cohort in 133 LMICs. **Figure S8.** LSHTM (UNIVAC) and PATH model comparison of the percentage of 133 LMICs willing to pay for RSV intervention compared to status quo.

## Data Availability

All data generated or analysed during this study are included in this published article [and its Additional file].

## Abbreviations

ALRI: Acute lower respiratory infection
ANC: Antenatal care
BCG: Bacillus Calmette-Guérin
CEAC: Cost-effectiveness acceptability curve
DALY: Disability adjusted life years
GDP: Gross domestic product
LMICs: Low-income and middle-income countries
mAb: Monoclonal antibody
PATH: Program for Appropriate Technology in Health
RSV: Respiratory syncytial virus
UN: United Nations
WHO: World Health Organization
WTP: Willingness-to-pay
