# Supplementary material for: Cost-effectiveness of pharmaceutical strategies to prevent respiratory syncytial virus disease in young children: a decision-support model for use in low-income and middle-income countries
Source: BMC Med. 2023 Apr 11;21:138. doi: 10.1186/s12916-023-02827-5 (PMC10088159; doi:10.1186/s12916-023-02827-5)
Supplement: Supplementary file 1 — Additional file 1. Additional details on methods, calculations, disease input parameters, results of the comparison exercise, sensitivity, and scenario analysis. Table S1-S13 and Figure S1-S8. Table S1. Definitions of disease categories. TableS2. Severe RSV disease, aged <5 years (per 100,000 children, per year). Table S3. Non-severe RSV, aged <5 years (per 100,000 children, per year). Table S4. Incidence of asthma in 2019, aged <5 years (per 100,000 children per year). Table S5. RSV mortality rate, aged <5 years (per 100,000 children per year). Table S6. Hospital admission rates, aged <5 years (per 100,000 children per year). Table S7. Incidence of clinic visits for severe RSV, aged 5 years (per 100,000 children, per year). Table S8. Incidence of clinic visits for non-severe RSV, aged 5 years (per 100,000 children, per year). Figure S1. Age distribution of severeRSV-ALRI cases in first year of life. Figure S2. Age distribution of non-severe and severe RSV-ALRI cases by age infirst year of life. Table S9. Estimates of maternal coverage (using ANC) as a proxy for maternal RSV vaccineand national immunization coverage of existing vaccines as a proxy for mAb. Figure S3. Efficacy scenarios used formaternal vaccination and monoclonal antibody. Table S10. WHO checklist for appraisal of economic evaluation andmodel comparison exercise. Table S11. Description of alternative scenarios. FigureS4. Percentage of 133 LMICs willing to pay for mAb RSV intervention. Figure S5. Percentage of 133 LMICswilling to pay for maternal vaccine RSV intervention. Figure S6. Percentage difference in cost per DALY averted in alow-income country, relative to baseline cost of US$3 for mAb and US$36, when each parameter is varied by +/-10%. Figure S7. Percentage difference in cost per DALY averted in a middle-income country, relative to baseline cost of US$733 for mAb and US$929 for maternal vaccine, when each parameter is varied by +/-10%. Table S12. Comparison of maternal vaccine estimates by UN [file 12916_2023_2827_MOESM1_ESM.docx]

**ADDITIONAL FILE 1**

**Cost-effectiveness of pharmaceutical strategies to prevent respiratory syncytial virus disease in young children: a decision-support model for use in low-income and middle-income countries**

Sarwat Mahmud, Ranju Baral, Colin Sanderson, Clint Pecenka, Mark Jit, You Li, Andrew Clark

**Contents**

UNIVAC disease categories **3**

**Table S1.** Definitions of disease categories **3**

UNIVAC model calculations**5**

Cost calculations **5**

Burden of RSV disease input parameters.**6**

*Severe RSV disease, aged <5 years.***6**

**Table S2.** Severe RSV disease, aged <5 years (per 100,000 children, per year) **6**

Non-severe RSV disease, aged <5 years**9**

**Table S3.** Non-severe RSV, aged <5 years (per 100,000 children, per year)**9**

*Incidence of asthma in 2019, aged <5 years (per 100,000 children per year)* **12**

**Table S4.** Incidence of asthma in 2019, aged <5 years (per 100,000 children per year)**12**

*RSV mortality, aged <5 years*…**15**

**Table S5.** RSV mortality rate, aged <5 years (per 100,000 children per year).**15**

*RSV hospital admissions, aged <5 years***18**

**Table S6.** Hospital admission rates, by narrow age bands (per 100,000 children per year)…**18**

*Clinic visits, aged <5 years***21**

**Table S7.** Incidence of clinic visits for non-severe RSV, by narrow age band (per 100,000 children, per year).**21**

**Table S8.** Incidence of clinic visits for non-severe RSV, aged 5 years (per 100,000 children, per year).**24**

*RSV disease age distribution, aged <5 years.***27**

**Figure S1**. Age distribution of severe RSV-ALRI cases in first year of life**27**

**Figure S2.** Age distribution of non-severe and severe RSV-ALRI cases by age in first year of life**28**

DALY weights and average duration of illness**28**

Disease treatment costs **29**

Coverage of RSV prevention strategies.**29**

**Table S9.** Estimates of maternal coverage (using ANC) as a proxy for maternal RSV vaccine and national immunization coverage of existing vaccines as a proxy for mAb..**30**

Efficacy of RSV intervention programs**32**

**Figure S3.** Efficacy scenarios used for maternal vaccination and monoclonal antibody…....**32**

Vaccine programme costs **33**

Methods used for economic evaluation of maternal vaccination and infant mAb in 122 LMICs**34**

**Table S10.** WHO checklist for appraisal of economic evaluation and model comparison exercise **34**

**Table S11.** Scenarios evaluated to identify influential inputs/assumption **43**

**Figure S4.** Percentage of 133 LMICs willing to pay for mAb RSV intervention. **44**

**Figure S5.** Percentage of 133 LMICs willing to pay for maternal vaccine RSV intervention.**45**

Sensitivity analysis**46**

**Figure S6**. Percentage difference in cost per DALY averted in a low-income country, relative to baseline cost of US$3 for mAb and US$36, when each parameter is varied by +/-10% **46**

**Figure S7.** Percentage difference in cost per DALY averted in a middle-income country, relative to baseline cost of US$733 for mAb and US$929 for maternal vaccine, when each parameter is varied by +/-10% **47**

Comparison exercise **48**

**Table S12.** Comparison of ***maternal vaccine*** estimates by UNIVAC model and PATH model for the 2025 birth cohort in 133 LMICs **48**

**Table S13.** Comparison of ***monoclonal antibody*** estimates by UNIVAC model and PATH model for the 2025 birth cohort in 133 LMICs **48**

**Figure S8**. LSHTM (UNIVAC) and PATH model comparison of the percentage of 133 LMICs willing to pay for RSV intervention compared to status quo **49**

**Possible definitions of RSV disease categories used in UNIVAC**

UNIVAC includes options to adapt the RSV disease categories and their outcomes (cases, clinic visits, admissions, and deaths) if required. National teams should ensure that the chosen RSV disease categories and outcomes align as closely as possible with available disease burden estimates and efficacy end points from clinical trials.

The number of disease categories, names of disease categories, and types of outcome can be determined by the user e.g. All-cause ALRI may be used instead of severe RSV-ALRI disease. However, users should ensure there is no overlap (double counting) of RSV disease across the categories considered. This may involve using and adapting existing case definitions, such as those used for severe acute respiratory infection (SARI) (20), Integrated Management of Childhood Illness (IMCI) (21), and influenza-like illness (ILI) (22). In addition, the WHO has piloted an RSV surveillance strategy, and one of the aims was to refine case definitions that are well-suited to RSV surveillance (20). Several studies have been conducted assessing the sensitivity and specificity of case definitions using SARI, IMCI, ILI, and more, and may be used to inform case definition decisions (23-26).

UNIVAC can also be set up to include ‘RSV disease sequelae’ (wheeze and asthma) but we anticipate that most national teams will exclude this from the base case scenario (i.e. the scenario with the most realistic set of assumptions) due to a shortage of data on the proportion of RSV disease survivors expected to develop sequelae, and uncertainty about the efficacy of RSV interventions on RSV sequelae (27). If included, the annual rate of RSV sequelae should be determined by subtracting the rate of RSV deaths from the rate of RSV disease cases (non-severe and severe combined) and multiplying by the proportion of RSV disease survivors expected to develop sequelae.

**Table S1.** Definitions of disease categories.

| **Disease** | **Definition** | **Reference** |
| --- | --- | --- |
| Severe RSV-ALRI | *Community based studies*   - cough or difficulty in breathing with one danger sign below (previously WHO very severe pneumonia): cyanosis, difficulty in breastfeeding or drinking, vomiting everything, convulsions, lethargy, unconsciousness, and head nodding   *Hospital based studies*   - Hospitalised ALRI with hypoxaemia as defined below: - At altitude ≤2 500 m above sea level, SpO2 <90% in children aged 1–<60 months and <88% for neonates (<1 month); at altitude >2 500 m above sea level, SpO2 <87% in children aged 1–<60 months and <85% for neonates (<1 month). - Hospitalised ALRI with one danger sign (cyanosis, difficulty in breastfeeding or drinking, vomiting everything, convulsions, lethargy, unconsciousness, and head nodding) OR proxies for very severe disease (e.g. mechanical ventilation OR ICU admission) — previously very severe pneumonia. | Li, 2022 (1) |
|  | - Cough or difficulty in breathing with chest wall indrawing and laboratory confirmed RSV - Increased RR (>60 breaths/ min) OR - Chest wall indrawing and laboratory confirmed RSV. | Shi, 2017 (28) |
|  | - Severe and very severe pneumonia case definition used as a proxy for severe RSV-ALRI - Cough or difficulty breathing with chest in-drawing when calm - Stridor when calm - Not-able to breastfeed/drink - Convulsions - Lethargy - Unconscious - Vomit everything | IMCI (21) |
|  | - Respiratory infection/cough which requires hospital admission and the onset is within the last 10 days. - Apnoea - Sepsis | SARI (20) |
|  | *Laboratory criterion*   - RSV infection as confirmed by a fit-for-purpose, fully validated PCR assay with high specificity and sufficient sensitivity on upper respiratory samples.   *Clinical criteria*   - Respiratory infection defined as a cough or difficult breathing AND - lower respiratory tract infection defined as fast breathing by WHO criteria or SpO_2_ < 95% AND - ≥1 of the following features of severe disease - Pulse oximetry < 93% - Lower chest wall in-drawing | WHO consultation on RSV Vaccine Development report, 2015 (29) |
|  | *Laboratory criterion*   - RSV infection confirmed on nasal swab by quantitative PCR.   *Clinical criteria*   - Oxygen saturation <92%, OR - Difficulty breathing leading to:   - Irritability/agitation, or - Lethargy/sleepiness, or - Severe chest indrawing, or  - Reduced/no vocalization, or - Apnoea >20 sec, or - Cyanosis, or  - Stop feeding well/dehydration. | GSK clinical trial (30) |
| Non-severe RSV-ALRI | - Cough or difficulty breathing with increased respiratory rate - Laboratory confirmed RSV | Shi, 2017 (28) |
|  | - Non-severe pneumonia case definitions used as a proxy for non-severe RSV-ALRI - Cough or difficulty breathing with fast breathing. - Fast breathing defined as:   - <2 months: ≥ 60 breaths/minute   - 2-12 months: ≥ 50 breaths/minute - 12-59 months: ≥ 40 breaths/minute | IMCI (21) |
|  | - - Three or more episodes of wheezing during the first year of life. | Blanken, 2013 (31) |
| All-cause recurrent wheezing | Long-term respiratory disease resulting in inflammation and narrowing of airways. Clinical features vary depending on severity. | WHO, Bousquet, 2010 (32, 33) |

**UNIVAC model calculations for the cost of RSV prevention strategies**

UNIVAC is designed for economic analysis rather than detailed planning and budgeting, and therefore focuses on costs and consequences of doses procured and administered, rather than costs associated with other planning considerations e.g. purchasing buffer stocks. When calculating the cost of prevention strategies, UNIVAC uses the mid-year population size for the number of people alive in the single year of age and single calendar year targeted by the intervention. For most vaccines evaluated in UNIVAC, this is usually the mid-year population for children aged <12 months. The mid-year population for children aged 12-23 months may also be used for to calculate programme costs for the small proportion of infant vaccines that are administered after age 12 months (due to the country having an older target age for a particular dose, or due to poor vaccine timeliness). Some vaccines (e.g. human papillomavirus vaccines) are administered at a much older age (e.g. 10 years) and use the mid-year population for the same year of age. For doses administered in a single dose at birth (eg mAb for RSV), a country team may prefer to use the number of live births as a basis for programme cost calculations, and UNIVAC includes an option to select this if required. For single dose maternal vaccination (e.g. RSV maternal vaccination) UNIVAC uses the number of late-stage pregnancies (live births plus stillbirths).

The total cost of an intervention programme in a given calendar year, *y*, single year of age, *a*, and dose *d*, is calculated as:

(M_y,a_ * C _y,a,d_ * (1 / (1 - W_v_)) * (P_v_ + P_v_ * H_v_ + P_v_ * T_v_)) ‘vaccine costs

+ (M_y,a_ * C _y,a,d_ * (1 / (1 - W_s_)) * (P_s_ + P_s_ * H_s_ + P_s_ * T_s_)) ‘syringe costs

+ (M_y,a_ * C _y,a,d_ * (1/ (1 - W_b_)) * (P_b_ + P_b_ * H_b_ + P_b_ * T_b_)) ‘safety box costs

+ (M_y,a_ * C _y,a,d_ * S) ‘system costs

Where: M is the mid-year population for a given calendar year, *y*, and single year of age, *a* (for maternal vaccination, this is calculated as the number of pregnant women i.e. live births + stillbirths); C is the incremental percentage coverage of dose *d* in year *y* and age *a*; W is the % wastage for vaccines *v*, syringes *s*, and safety boxes, *b*. P price per dose for vaccines v, syringes s, and safety boxes, b. H is the international handling fee, eg for the UNICEF supply division or PAHO revolving fund, expressed as a % of the price per dose for vaccines *v*, syringes *s*, and safety boxes, *b*. T is the international transportation fee, including insurance, expressed as a % of the price per dose for vaccines *v*, syringes *s*, and safety boxes, *b*. S is the incremental health system cost per dose. This refers to any additional financial costs (e.g. vehicles, refrigerators, new vaccination cards etc.) and economic costs (e.g. nurse time, cold chain manager time etc.) incurred by the health system as a result of introducing the new RSV intervention.

**Sources of recommended data included in UNIVAC**

UNIVAC includes recommended input data for all required model parameters in 133 LMICs. Where possible, these should be improved at country level following consultation with national stakeholders. Most of the disease burden parameters were based on recent disease burden estimates by Li et al (1), which were available by the income status of the country (low income, lower middle income, upper middle income).

1. *Rate of severe RSV disease, aged <5 years.*

RSV incidence estimates for chest wall indrawing were used as a proxy for severe RSV disease (1). Estimates for children aged <5 years by income status were extracted and converted into the standard format required by UNIVAC i.e. per 100,000 per year. The estimate was not available for upper-middle income, so we assumed 1/8^th^ of the rate for all-severity RSV cases in upper-middle income countries were severe (same fraction observed in high income countries (1)).

**Table S2.** Severe RSV disease, aged <5 years (per 100,000 children, per year).

| **Country** | **Income** | **Incidence of severe RSV** | | | **Source** |
| --- | --- | --- | --- | --- | --- |
|  |  | **Mid** | **Low** | **High** |  |
| Afghanistan | LI | 480 | 50 | 4,520 | Li, 2022 (1) |
| Albania | UMI | 690 | 423 | 1,998 | Li, 2022 (1) |
| Algeria | UMI | 690 | 423 | 1,998 | Li, 2022 (1) |
| Angola | LMI | 1,400 | 800 | 2,420 | Li, 2022 (1) |
| Argentina | UMI | 690 | 423 | 1,998 | Li, 2022 (1) |
| Armenia | LMI | 1,400 | 800 | 2,420 | Li, 2022 (1) |
| Azerbaijan | UMI | 690 | 423 | 1,998 | Li, 2022 (1) |
| Bangladesh | LMI | 1,400 | 800 | 2,420 | Li, 2022 (1) |
| Belarus | UMI | 690 | 423 | 1,998 | Li, 2022 (1) |
| Belize | UMI | 690 | 423 | 1,998 | Li, 2022 (1) |
| Benin | LI | 480 | 50 | 4,520 | Li, 2022 (1) |
| Bhutan | LMI | 1,400 | 800 | 2,420 | Li, 2022 (1) |
| Bolivia (Plurinational State of) | LMI | 1,400 | 800 | 2,420 | Li, 2022 (1) |
| Bosnia and Herzegovina | UMI | 690 | 423 | 1,998 | Li, 2022 (1) |
| Botswana | UMI | 690 | 423 | 1,998 | Li, 2022 (1) |
| Brazil | UMI | 690 | 423 | 1,998 | Li, 2022 (1) |
| Bulgaria | UMI | 690 | 423 | 1,998 | Li, 2022 (1) |
| Burkina Faso | LI | 480 | 50 | 4,520 | Li, 2022 (1) |
| Burundi | LI | 480 | 50 | 4,520 | Li, 2022 (1) |
| Cabo Verde | LMI | 1,400 | 800 | 2,420 | Li, 2022 (1) |
| Cambodia | LMI | 1,400 | 800 | 2,420 | Li, 2022 (1) |
| Cameroon | LMI | 1,400 | 800 | 2,420 | Li, 2022 (1) |
| Central African Republic | LI | 480 | 50 | 4,520 | Li, 2022 (1) |
| Chad | LI | 480 | 50 | 4,520 | Li, 2022 (1) |
| China | UMI | 690 | 423 | 1,998 | Li, 2022 (1) |
| Colombia | UMI | 690 | 423 | 1,998 | Li, 2022 (1) |
| Comoros | LI | 480 | 50 | 4,520 | Li, 2022 (1) |
| Congo | LMI | 1,400 | 800 | 2,420 | Li, 2022 (1) |
| Costa Rica | UMI | 690 | 423 | 1,998 | Li, 2022 (1) |
| Côte d'Ivoire | LMI | 1,400 | 800 | 2,420 | Li, 2022 (1) |
| Croatia | UMI | 690 | 423 | 1,998 | Li, 2022 (1) |
| Cuba | UMI | 690 | 423 | 1,998 | Li, 2022 (1) |
| Dem. People's Republic of Korea | LI | 480 | 50 | 4,520 | Li, 2022 (1) |
| Democratic Republic of the Congo | LI | 480 | 50 | 4,520 | Li, 2022 (1) |
| Djibouti | LMI | 1,400 | 800 | 2,420 | Li, 2022 (1) |
| Dominican Republic | UMI | 690 | 423 | 1,998 | Li, 2022 (1) |
| Ecuador | UMI | 690 | 423 | 1,998 | Li, 2022 (1) |
| Egypt | LMI | 1,400 | 800 | 2,420 | Li, 2022 (1) |
| El Salvador | LMI | 1,400 | 800 | 2,420 | Li, 2022 (1) |
| Equatorial Guinea | UMI | 690 | 423 | 1,998 | Li, 2022 (1) |
| Eritrea | LI | 480 | 50 | 4,520 | Li, 2022 (1) |
| Ethiopia | LI | 480 | 50 | 4,520 | Li, 2022 (1) |
| Fiji | UMI | 690 | 423 | 1,998 | Li, 2022 (1) |
| Gabon | UMI | 690 | 423 | 1,998 | Li, 2022 (1) |
| Gambia | LI | 480 | 50 | 4,520 | Li, 2022 (1) |
| Georgia | LMI | 1,400 | 800 | 2,420 | Li, 2022 (1) |
| Ghana | LMI | 1,400 | 800 | 2,420 | Li, 2022 (1) |
| Grenada | UMI | 690 | 423 | 1,998 | Li, 2022 (1) |
| Guatemala | LMI | 1,400 | 800 | 2,420 | Li, 2022 (1) |
| Guinea | LI | 480 | 50 | 4,520 | Li, 2022 (1) |
| Guinea-Bissau | LI | 480 | 50 | 4,520 | Li, 2022 (1) |
| Guyana | UMI | 690 | 423 | 1,998 | Li, 2022 (1) |
| Haiti | LI | 480 | 50 | 4,520 | Li, 2022 (1) |
| Honduras | LMI | 1,400 | 800 | 2,420 | Li, 2022 (1) |
| India | LMI | 1,400 | 800 | 2,420 | Li, 2022 (1) |
| Indonesia | LMI | 1,400 | 800 | 2,420 | Li, 2022 (1) |
| Iran (Islamic Republic of) | UMI | 690 | 423 | 1,998 | Li, 2022 (1) |
| Iraq | UMI | 690 | 423 | 1,998 | Li, 2022 (1) |
| Jamaica | UMI | 690 | 423 | 1,998 | Li, 2022 (1) |
| Jordan | LMI | 1,400 | 800 | 2,420 | Li, 2022 (1) |
| Kazakhstan | UMI | 690 | 423 | 1,998 | Li, 2022 (1) |
| Kenya | LMI | 1,400 | 800 | 2,420 | Li, 2022 (1) |
| Kiribati | LMI | 1,400 | 800 | 2,420 | Li, 2022 (1) |
| Kosovo | LMI | 1,400 | 800 | 2,420 | Li, 2022 (1) |
| Kyrgyzstan | LMI | 1,400 | 800 | 2,420 | Li, 2022 (1) |
| Lao People's Democratic Republic | LMI | 1,400 | 800 | 2,420 | Li, 2022 (1) |
| Lebanon | UMI | 690 | 423 | 1,998 | Li, 2022 (1) |
| Lesotho | LMI | 1,400 | 800 | 2,420 | Li, 2022 (1) |
| Liberia | LI | 480 | 50 | 4,520 | Li, 2022 (1) |
| Libya | UMI | 690 | 423 | 1,998 | Li, 2022 (1) |
| Madagascar | LI | 480 | 50 | 4,520 | Li, 2022 (1) |
| Malawi | LI | 480 | 50 | 4,520 | Li, 2022 (1) |
| Malaysia | UMI | 690 | 423 | 1,998 | Li, 2022 (1) |
| Maldives | UMI | 690 | 423 | 1,998 | Li, 2022 (1) |
| Mali | LI | 480 | 50 | 4,520 | Li, 2022 (1) |
| Marshall Islands | UMI | 690 | 423 | 1,998 | Li, 2022 (1) |
| Mauritania | LMI | 1,400 | 800 | 2,420 | Li, 2022 (1) |
| Mauritius | UMI | 690 | 423 | 1,998 | Li, 2022 (1) |
| Mexico | UMI | 690 | 423 | 1,998 | Li, 2022 (1) |
| Micronesia (Fed. States of) | LMI | 1,400 | 800 | 2,420 | Li, 2022 (1) |
| Mongolia | LMI | 1,400 | 800 | 2,420 | Li, 2022 (1) |
| Montenegro | UMI | 690 | 423 | 1,998 | Li, 2022 (1) |
| Morocco | LMI | 1,400 | 800 | 2,420 | Li, 2022 (1) |
| Mozambique | LI | 480 | 50 | 4,520 | Li, 2022 (1) |
| Myanmar | LMI | 1,400 | 800 | 2,420 | Li, 2022 (1) |
| Namibia | UMI | 690 | 423 | 1,998 | Li, 2022 (1) |
| Nepal | LI | 480 | 50 | 4,520 | Li, 2022 (1) |
| Nicaragua | LMI | 1,400 | 800 | 2,420 | Li, 2022 (1) |
| Niger | LI | 480 | 50 | 4,520 | Li, 2022 (1) |
| Nigeria | LMI | 1,400 | 800 | 2,420 | Li, 2022 (1) |
| Pakistan | LMI | 1,400 | 800 | 2,420 | Li, 2022 (1) |
| State of Palestine | LMI | 1,400 | 800 | 2,420 | Li, 2022 (1) |
| Panama | UMI | 690 | 423 | 1,998 | Li, 2022 (1) |
| Papua New Guinea | LMI | 1,400 | 800 | 2,420 | Li, 2022 (1) |
| Paraguay | UMI | 690 | 423 | 1,998 | Li, 2022 (1) |
| Peru | UMI | 690 | 423 | 1,998 | Li, 2022 (1) |
| Philippines | LMI | 1,400 | 800 | 2,420 | Li, 2022 (1) |
| Republic of Moldova | LMI | 1,400 | 800 | 2,420 | Li, 2022 (1) |
| Romania | UMI | 690 | 423 | 1,998 | Li, 2022 (1) |
| Russian Federation | UMI | 690 | 423 | 1,998 | Li, 2022 (1) |
| Rwanda | LI | 480 | 50 | 4,520 | Li, 2022 (1) |
| Saint Lucia | UMI | 690 | 423 | 1,998 | Li, 2022 (1) |
| Saint Vincent and the Grenadines | UMI | 690 | 423 | 1,998 | Li, 2022 (1) |
| Samoa | UMI | 690 | 423 | 1,998 | Li, 2022 (1) |
| Sao Tome and Principe | LMI | 1,400 | 800 | 2,420 | Li, 2022 (1) |
| Senegal | LI | 480 | 50 | 4,520 | Li, 2022 (1) |
| Serbia | UMI | 690 | 423 | 1,998 | Li, 2022 (1) |
| Sierra Leone | LI | 480 | 50 | 4,520 | Li, 2022 (1) |
| Solomon Islands | LMI | 1,400 | 800 | 2,420 | Li, 2022 (1) |
| Somalia | LI | 480 | 50 | 4,520 | Li, 2022 (1) |
| South Africa | UMI | 690 | 423 | 1,998 | Li, 2022 (1) |
| South Sudan | LI | 480 | 50 | 4,520 | Li, 2022 (1) |
| Sri Lanka | LMI | 1,400 | 800 | 2,420 | Li, 2022 (1) |
| Sudan | LMI | 1,400 | 800 | 2,420 | Li, 2022 (1) |
| Suriname | UMI | 690 | 423 | 1,998 | Li, 2022 (1) |
| Eswatini | LMI | 1,400 | 800 | 2,420 | Li, 2022 (1) |
| Syrian Arab Republic | LMI | 1,400 | 800 | 2,420 | Li, 2022 (1) |
| Tajikistan | LMI | 1,400 | 800 | 2,420 | Li, 2022 (1) |
| North Macedonia | UMI | 690 | 423 | 1,998 | Li, 2022 (1) |
| Thailand | UMI | 690 | 423 | 1,998 | Li, 2022 (1) |
| Timor-Leste | LMI | 1,400 | 800 | 2,420 | Li, 2022 (1) |
| Togo | LI | 480 | 50 | 4,520 | Li, 2022 (1) |
| Tonga | UMI | 690 | 423 | 1,998 | Li, 2022 (1) |
| Tunisia | LMI | 1,400 | 800 | 2,420 | Li, 2022 (1) |
| Turkey | UMI | 690 | 423 | 1,998 | Li, 2022 (1) |
| Turkmenistan | UMI | 690 | 423 | 1,998 | Li, 2022 (1) |
| Tuvalu | UMI | 690 | 423 | 1,998 | Li, 2022 (1) |
| Uganda | LI | 480 | 50 | 4,520 | Li, 2022 (1) |
| Ukraine | LMI | 1,400 | 800 | 2,420 | Li, 2022 (1) |
| United Republic of Tanzania | LI | 480 | 50 | 4,520 | Li, 2022 (1) |
| Uzbekistan | LMI | 1,400 | 800 | 2,420 | Li, 2022 (1) |
| Vanuatu | LMI | 1,400 | 800 | 2,420 | Li, 2022 (1) |
| Venezuela (Bolivarian Republic of) | UMI | 690 | 423 | 1,998 | Li, 2022 (1) |
| Vietnam | LMI | 1,400 | 800 | 2,420 | Li, 2022 (1) |
| Yemen | LMI | 1,400 | 800 | 2,420 | Li, 2022 (1) |
| Zambia | LMI | 1,400 | 800 | 2,420 | Li, 2022 (1) |
| Zimbabwe | LI | 480 | 50 | 4,520 | Li, 2022 (1) |

Acronym: LI, low income; LMI, low-middle income; RSV, respiratory syncytial virus; UMI, upper middle income.

Users may consider using ICD-10 codes to estimate the burden of disease as an alternative to default data in the UNIVAC model, however the data must be used with caution. Using RSV specific ICD-10 codes has been shown to underestimate the burden of RSV infections (42). Even if used in combination with general ALRI ICD-10 codes, as recommended (42), the resulting estimates may be biased by differences in coding behaviour, such as in that between primary and secondary care.

1. *Rate of non-severe RSV disease, aged <5 years.*

The incidence of non-severe RSV disease was estimated by subtracting the incidence of severe RSV from the incidence of all-severity RSV by income status (low income, lower middle income, upper middle income) (1) and converting this into the standard format required by UNIVAC i.e. per 100,000 per year.

**Table S3.** Non-severe RSV, aged <5 years (per 100,000 children, per year).

| **Country** | **Income** | **Incidence of non-severe RSV** | | | **Source** |
| --- | --- | --- | --- | --- | --- |
|  |  | **Mid** | **Low** | **High** |  |
| Afghanistan | LI | 4,450 | 2,654 | 7,474 | Li, 2022 (1) |
| Albania | UMI | 4,830 | 2,223 | 10,491 | Li, 2022 (1) |
| Algeria | UMI | 4,830 | 2,223 | 10,491 | Li, 2022 (1) |
| Angola | LMI | 3,740 | 2,750 | 5,079 | Li, 2022 (1) |
| Argentina | UMI | 4,830 | 2,223 | 10,491 | Li, 2022 (1) |
| Armenia | LMI | 3,740 | 2,750 | 5,079 | Li, 2022 (1) |
| Azerbaijan | UMI | 4,830 | 2,223 | 10,491 | Li, 2022 (1) |
| Bangladesh | LMI | 3,740 | 2,750 | 5,079 | Li, 2022 (1) |
| Belarus | UMI | 4,830 | 2,223 | 10,491 | Li, 2022 (1) |
| Belize | UMI | 4,830 | 2,223 | 10,491 | Li, 2022 (1) |
| Benin | LI | 4,450 | 2,654 | 7,474 | Li, 2022 (1) |
| Bhutan | LMI | 3,740 | 2,750 | 5,079 | Li, 2022 (1) |
| Bolivia (Plurinational State of) | LMI | 3,740 | 2,750 | 5,079 | Li, 2022 (1) |
| Bosnia and Herzegovina | UMI | 4,830 | 2,223 | 10,491 | Li, 2022 (1) |
| Botswana | UMI | 4,830 | 2,223 | 10,491 | Li, 2022 (1) |
| Brazil | UMI | 4,830 | 2,223 | 10,491 | Li, 2022 (1) |
| Bulgaria | UMI | 4,830 | 2,223 | 10,491 | Li, 2022 (1) |
| Burkina Faso | LI | 4,450 | 2,654 | 7,474 | Li, 2022 (1) |
| Burundi | LI | 4,450 | 2,654 | 7,474 | Li, 2022 (1) |
| Cabo Verde | LMI | 3,740 | 2,750 | 5,079 | Li, 2022 (1) |
| Cambodia | LMI | 3,740 | 2,750 | 5,079 | Li, 2022 (1) |
| Cameroon | LMI | 3,740 | 2,750 | 5,079 | Li, 2022 (1) |
| Central African Republic | LI | 4,450 | 2,654 | 7,474 | Li, 2022 (1) |
| Chad | LI | 4,450 | 2,654 | 7,474 | Li, 2022 (1) |
| China | UMI | 4,830 | 2,223 | 10,491 | Li, 2022 (1) |
| Colombia | UMI | 4,830 | 2,223 | 10,491 | Li, 2022 (1) |
| Comoros | LI | 4,450 | 2,654 | 7,474 | Li, 2022 (1) |
| Congo | LMI | 3,740 | 2,750 | 5,079 | Li, 2022 (1) |
| Costa Rica | UMI | 4,830 | 2,223 | 10,491 | Li, 2022 (1) |
| Côte d'Ivoire | LMI | 3,740 | 2,750 | 5,079 | Li, 2022 (1) |
| Croatia | UMI | 4,830 | 2,223 | 10,491 | Li, 2022 (1) |
| Cuba | UMI | 4,830 | 2,223 | 10,491 | Li, 2022 (1) |
| Dem. People's Republic of Korea | LI | 4,450 | 2,654 | 7,474 | Li, 2022 (1) |
| Democratic Republic of the Congo | LI | 4,450 | 2,654 | 7,474 | Li, 2022 (1) |
| Djibouti | LMI | 3,740 | 2,750 | 5,079 | Li, 2022 (1) |
| Dominican Republic | UMI | 4,830 | 2,223 | 10,491 | Li, 2022 (1) |
| Ecuador | UMI | 4,830 | 2,223 | 10,491 | Li, 2022 (1) |
| Egypt | LMI | 3,740 | 2,750 | 5,079 | Li, 2022 (1) |
| El Salvador | LMI | 3,740 | 2,750 | 5,079 | Li, 2022 (1) |
| Equatorial Guinea | UMI | 4,830 | 2,223 | 10,491 | Li, 2022 (1) |
| Eritrea | LI | 4,450 | 2,654 | 7,474 | Li, 2022 (1) |
| Ethiopia | LI | 4,450 | 2,654 | 7,474 | Li, 2022 (1) |
| Fiji | UMI | 4,830 | 2,223 | 10,491 | Li, 2022 (1) |
| Gabon | UMI | 4,830 | 2,223 | 10,491 | Li, 2022 (1) |
| Gambia | LI | 4,450 | 2,654 | 7,474 | Li, 2022 (1) |
| Georgia | LMI | 3,740 | 2,750 | 5,079 | Li, 2022 (1) |
| Ghana | LMI | 3,740 | 2,750 | 5,079 | Li, 2022 (1) |
| Grenada | UMI | 4,830 | 2,223 | 10,491 | Li, 2022 (1) |
| Guatemala | LMI | 3,740 | 2,750 | 5,079 | Li, 2022 (1) |
| Guinea | LI | 4,450 | 2,654 | 7,474 | Li, 2022 (1) |
| Guinea-Bissau | LI | 4,450 | 2,654 | 7,474 | Li, 2022 (1) |
| Guyana | UMI | 4,830 | 2,223 | 10,491 | Li, 2022 (1) |
| Haiti | LI | 4,450 | 2,654 | 7,474 | Li, 2022 (1) |
| Honduras | LMI | 3,740 | 2,750 | 5,079 | Li, 2022 (1) |
| India | LMI | 3,740 | 2,750 | 5,079 | Li, 2022 (1) |
| Indonesia | LMI | 3,740 | 2,750 | 5,079 | Li, 2022 (1) |
| Iran (Islamic Republic of) | UMI | 4,830 | 2,223 | 10,491 | Li, 2022 (1) |
| Iraq | UMI | 4,830 | 2,223 | 10,491 | Li, 2022 (1) |
| Jamaica | UMI | 4,830 | 2,223 | 10,491 | Li, 2022 (1) |
| Jordan | LMI | 3,740 | 2,750 | 5,079 | Li, 2022 (1) |
| Kazakhstan | UMI | 4,830 | 2,223 | 10,491 | Li, 2022 (1) |
| Kenya | LMI | 3,740 | 2,750 | 5,079 | Li, 2022 (1) |
| Kiribati | LMI | 3,740 | 2,750 | 5,079 | Li, 2022 (1) |
| Kosovo | LMI | 3,740 | 2,750 | 5,079 | Li, 2022 (1) |
| Kyrgyzstan | LMI | 3,740 | 2,750 | 5,079 | Li, 2022 (1) |
| Lao People's Democratic Republic | LMI | 3,740 | 2,750 | 5,079 | Li, 2022 (1) |
| Lebanon | UMI | 4,830 | 2,223 | 10,491 | Li, 2022 (1) |
| Lesotho | LMI | 3,740 | 2,750 | 5,079 | Li, 2022 (1) |
| Liberia | LI | 4,450 | 2,654 | 7,474 | Li, 2022 (1) |
| Libya | UMI | 4,830 | 2,223 | 10,491 | Li, 2022 (1) |
| Madagascar | LI | 4,450 | 2,654 | 7,474 | Li, 2022 (1) |
| Malawi | LI | 4,450 | 2,654 | 7,474 | Li, 2022 (1) |
| Malaysia | UMI | 4,830 | 2,223 | 10,491 | Li, 2022 (1) |
| Maldives | UMI | 4,830 | 2,223 | 10,491 | Li, 2022 (1) |
| Mali | LI | 4,450 | 2,654 | 7,474 | Li, 2022 (1) |
| Marshall Islands | UMI | 4,830 | 2,223 | 10,491 | Li, 2022 (1) |
| Mauritania | LMI | 3,740 | 2,750 | 5,079 | Li, 2022 (1) |
| Mauritius | UMI | 4,830 | 2,223 | 10,491 | Li, 2022 (1) |
| Mexico | UMI | 4,830 | 2,223 | 10,491 | Li, 2022 (1) |
| Micronesia (Fed. States of) | LMI | 3,740 | 2,750 | 5,079 | Li, 2022 (1) |
| Mongolia | LMI | 3,740 | 2,750 | 5,079 | Li, 2022 (1) |
| Montenegro | UMI | 4,830 | 2,223 | 10,491 | Li, 2022 (1) |
| Morocco | LMI | 3,740 | 2,750 | 5,079 | Li, 2022 (1) |
| Mozambique | LI | 4,450 | 2,654 | 7,474 | Li, 2022 (1) |
| Myanmar | LMI | 3,740 | 2,750 | 5,079 | Li, 2022 (1) |
| Namibia | UMI | 4,830 | 2,223 | 10,491 | Li, 2022 (1) |
| Nepal | LI | 4,450 | 2,654 | 7,474 | Li, 2022 (1) |
| Nicaragua | LMI | 3,740 | 2,750 | 5,079 | Li, 2022 (1) |
| Niger | LI | 4,450 | 2,654 | 7,474 | Li, 2022 (1) |
| Nigeria | LMI | 3,740 | 2,750 | 5,079 | Li, 2022 (1) |
| Pakistan | LMI | 3,740 | 2,750 | 5,079 | Li, 2022 (1) |
| State of Palestine | LMI | 3,740 | 2,750 | 5,079 | Li, 2022 (1) |
| Panama | UMI | 4,830 | 2,223 | 10,491 | Li, 2022 (1) |
| Papua New Guinea | LMI | 3,740 | 2,750 | 5,079 | Li, 2022 (1) |
| Paraguay | UMI | 4,830 | 2,223 | 10,491 | Li, 2022 (1) |
| Peru | UMI | 4,830 | 2,223 | 10,491 | Li, 2022 (1) |
| Philippines | LMI | 3,740 | 2,750 | 5,079 | Li, 2022 (1) |
| Republic of Moldova | LMI | 3,740 | 2,750 | 5,079 | Li, 2022 (1) |
| Romania | UMI | 4,830 | 2,223 | 10,491 | Li, 2022 (1) |
| Russian Federation | UMI | 4,830 | 2,223 | 10,491 | Li, 2022 (1) |
| Rwanda | LI | 4,450 | 2,654 | 7,474 | Li, 2022 (1) |
| Saint Lucia | UMI | 4,830 | 2,223 | 10,491 | Li, 2022 (1) |
| Saint Vincent and the Grenadines | UMI | 4,830 | 2,223 | 10,491 | Li, 2022 (1) |
| Samoa | UMI | 4,830 | 2,223 | 10,491 | Li, 2022 (1) |
| Sao Tome and Principe | LMI | 3,740 | 2,750 | 5,079 | Li, 2022 (1) |
| Senegal | LI | 4,450 | 2,654 | 7,474 | Li, 2022 (1) |
| Serbia | UMI | 4,830 | 2,223 | 10,491 | Li, 2022 (1) |
| Sierra Leone | LI | 4,450 | 2,654 | 7,474 | Li, 2022 (1) |
| Solomon Islands | LMI | 3,740 | 2,750 | 5,079 | Li, 2022 (1) |
| Somalia | LI | 4,450 | 2,654 | 7,474 | Li, 2022 (1) |
| South Africa | UMI | 4,830 | 2,223 | 10,491 | Li, 2022 (1) |
| South Sudan | LI | 4,450 | 2,654 | 7,474 | Li, 2022 (1) |
| Sri Lanka | LMI | 3,740 | 2,750 | 5,079 | Li, 2022 (1) |
| Sudan | LMI | 3,740 | 2,750 | 5,079 | Li, 2022 (1) |
| Suriname | UMI | 4,830 | 2,223 | 10,491 | Li, 2022 (1) |
| Eswatini | LMI | 3,740 | 2,750 | 5,079 | Li, 2022 (1) |
| Syrian Arab Republic | LMI | 3,740 | 2,750 | 5,079 | Li, 2022 (1) |
| Tajikistan | LMI | 3,740 | 2,750 | 5,079 | Li, 2022 (1) |
| North Macedonia | UMI | 4,830 | 2,223 | 10,491 | Li, 2022 (1) |
| Thailand | UMI | 4,830 | 2,223 | 10,491 | Li, 2022 (1) |
| Timor-Leste | LMI | 3,740 | 2,750 | 5,079 | Li, 2022 (1) |
| Togo | LI | 4,450 | 2,654 | 7,474 | Li, 2022 (1) |
| Tonga | UMI | 4,830 | 2,223 | 10,491 | Li, 2022 (1) |
| Tunisia | LMI | 3,740 | 2,750 | 5,079 | Li, 2022 (1) |
| Turkey | UMI | 4,830 | 2,223 | 10,491 | Li, 2022 (1) |
| Turkmenistan | UMI | 4,830 | 2,223 | 10,491 | Li, 2022 (1) |
| Tuvalu | UMI | 4,830 | 2,223 | 10,491 | Li, 2022 (1) |
| Uganda | LI | 4,450 | 2,654 | 7,474 | Li, 2022 (1) |
| Ukraine | LMI | 3,740 | 2,750 | 5,079 | Li, 2022 (1) |
| United Republic of Tanzania | LI | 4,450 | 2,654 | 7,474 | Li, 2022 (1) |
| Uzbekistan | LMI | 3,740 | 2,750 | 5,079 | Li, 2022 (1) |
| Vanuatu | LMI | 3,740 | 2,750 | 5,079 | Li, 2022 (1) |
| Venezuela (Bolivarian Republic of) | UMI | 4,830 | 2,223 | 10,491 | Li, 2022 (1) |
| Viet Nam | LMI | 3,740 | 2,750 | 5,079 | Li, 2022 (1) |
| Yemen | LMI | 3,740 | 2,750 | 5,079 | Li, 2022 (1) |
| Zambia | LMI | 3,740 | 2,750 | 5,079 | Li, 2022 (1) |
| Zimbabwe | LI | 4,450 | 2,654 | 7,474 | Li, 2022 (1) |

Acronym: LI, low income; LMI, low-middle income; RSV, respiratory syncytial virus; UMI, upper middle income.

Users may consider using ICD-10 codes to estimate the burden of disease as an alternative to default data in the UNIVAC model, however the data must be used with caution. Using RSV specific ICD-10 codes has been shown to underestimate the burden of RSV infections (42). Even if used in combination with general ALRI ICD-10 codes, as recommended (42), the resulting estimates may be biased by differences in coding behaviour, such as in that between primary and secondary care.

1. *Rate of recurrent wheezing, aged <5 years.*

We identified potential inputs for the incidence of recurrent wheezing and report them here for completeness. However, this disease category was not considered in the cost-effectiveness analysis reported in the main paper due to uncertainty around the possible impact of RSV prevention strategies on this outcome. Due to the paucity of data. the incidence of asthma was used as a proxy for recurrent wheezing, using data from GBD, 2019 (43).

**Table S4.** Incidence of asthma in 2019, aged <5 years (per 100,000 children per year).

| **Country** | **Incidence of asthma** | | | **Source** |
| --- | --- | --- | --- | --- |
|  | **Mid** | **Low** | **High** |  |
| Afghanistan | 1,477 | 893 | 2,306 | GBD, 2019 (43) |
| Albania | 1,135 | 667 | 1,794 | GBD, 2019 (43) |
| Algeria | 1,577 | 930 | 2,474 | GBD, 2019 (43) |
| Angola | 1,472 | 952 | 2,217 | GBD, 2019 (43) |
| Argentina | 2,242 | 1,324 | 3,470 | GBD, 2019 (43) |
| Armenia | 988 | 540 | 1,640 | GBD, 2019 (43) |
| Azerbaijan | 955 | 531 | 1,595 | GBD, 2019 (43) |
| Bangladesh | 272 | 155 | 433 | GBD, 2019 (43) |
| Belarus | 1,451 | 814 | 2,437 | GBD, 2019 (43) |
| Belize | 3,744 | 2,355 | 5,551 | GBD, 2019 (43) |
| Benin | 1,059 | 641 | 1,735 | GBD, 2019 (43) |
| Bhutan | 312 | 193 | 482 | GBD, 2019 (43) |
| Bolivia (Plurinational State of) | 3,668 | 2,387 | 5,445 | GBD, 2019 (43) |
| Bosnia and Herzegovina | 1,658 | 954 | 2,637 | GBD, 2019 (43) |
| Botswana | 884 | 522 | 1,376 | GBD, 2019 (43) |
| Brazil | 3,552 | 2,146 | 5,568 | GBD, 2019 (43) |
| Bulgaria | 1,431 | 844 | 2,283 | GBD, 2019 (43) |
| Burkina Faso | 1,178 | 752 | 1,841 | GBD, 2019 (43) |
| Burundi | 1,987 | 1,308 | 2,918 | GBD, 2019 (43) |
| Cabo Verde | 1,142 | 667 | 1,910 | GBD, 2019 (43) |
| Cambodia | 1,246 | 765 | 1,893 | GBD, 2019 (43) |
| Cameroon | 972 | 569 | 1,594 | GBD, 2019 (43) |
| Central African Republic | 1,540 | 1,044 | 2,226 | GBD, 2019 (43) |
| Chad | 895 | 553 | 1,421 | GBD, 2019 (43) |
| China | 1,381 | 777 | 2,287 | GBD, 2019 (43) |
| Colombia | 2,208 | 1,325 | 3,406 | GBD, 2019 (43) |
| Comoros | 1,925 | 1,223 | 2,924 | GBD, 2019 (43) |
| Congo | 1,587 | 982 | 2,464 | GBD, 2019 (43) |
| Costa Rica | 2,983 | 1,770 | 4,576 | GBD, 2019 (43) |
| C√¥te d'Ivoire | 1,163 | 688 | 1,911 | GBD, 2019 (43) |
| Croatia | 1,507 | 877 | 2,412 | GBD, 2019 (43) |
| Cuba | 4,600 | 2,844 | 6,776 | GBD, 2019 (43) |
| Democratic People's Republic of Korea | 1,600 | 955 | 2,519 | GBD, 2019 (43) |
| Democratic Republic of the Congo | 1,418 | 925 | 2,120 | GBD, 2019 (43) |
| Djibouti | 1,948 | 1,246 | 2,882 | GBD, 2019 (43) |
| Dominican Republic | 2,614 | 1,681 | 3,905 | GBD, 2019 (43) |
| Ecuador | 3,594 | 2,212 | 5,376 | GBD, 2019 (43) |
| Egypt | 1,447 | 907 | 2,296 | GBD, 2019 (43) |
| El Salvador | 2,893 | 1,749 | 4,399 | GBD, 2019 (43) |
| Equatorial Guinea | 1,485 | 913 | 2,288 | GBD, 2019 (43) |
| Eritrea | 1,925 | 1,256 | 2,851 | GBD, 2019 (43) |
| Ethiopia | 1,583 | 948 | 2,493 | GBD, 2019 (43) |
| Fiji | 1,091 | 683 | 1,683 | GBD, 2019 (43) |
| Gabon | 1,388 | 846 | 2,208 | GBD, 2019 (43) |
| Gambia | 1,038 | 622 | 1,643 | GBD, 2019 (43) |
| Georgia | 1,040 | 598 | 1,700 | GBD, 2019 (43) |
| Ghana | 918 | 550 | 1,487 | GBD, 2019 (43) |
| Grenada | 4,466 | 2,746 | 6,654 | GBD, 2019 (43) |
| Guatemala | 1,884 | 1,182 | 2,905 | GBD, 2019 (43) |
| Guinea | 1,215 | 725 | 1,977 | GBD, 2019 (43) |
| Guinea-Bissau | 1,012 | 599 | 1,624 | GBD, 2019 (43) |
| Guyana | 3,610 | 2,365 | 5,314 | GBD, 2019 (43) |
| Haiti | 4,522 | 3,161 | 6,136 | GBD, 2019 (43) |
| Honduras | 2,444 | 1,568 | 3,739 | GBD, 2019 (43) |
| India | 601 | 330 | 997 | GBD, 2019 (43) |
| Indonesia | 1,475 | 902 | 2,321 | GBD, 2019 (43) |
| Iran (Islamic Republic of) | 1,492 | 876 | 2,368 | GBD, 2019 (43) |
| Iraq | 1,668 | 982 | 2,617 | GBD, 2019 (43) |
| Jamaica | 3,997 | 2,484 | 5,922 | GBD, 2019 (43) |
| Jordan | 1,932 | 1,149 | 2,967 | GBD, 2019 (43) |
| Kazakhstan | 764 | 423 | 1,231 | GBD, 2019 (43) |
| Kenya | 1,441 | 850 | 2,313 | GBD, 2019 (43) |
| Kiribati | 1,149 | 784 | 1,652 | GBD, 2019 (43) |
| Kosovo | 1,378 | 2,155 | 812 | GBD, 2019 (43) |
| Kyrgyzstan | 1,071 | 607 | 1,817 | GBD, 2019 (43) |
| Lao People's Democratic Republic | 957 | 611 | 1,470 | GBD, 2019 (43) |
| Lebanon | 1,878 | 1,143 | 2,917 | GBD, 2019 (43) |
| Lesotho | 406 | 241 | 653 | GBD, 2019 (43) |
| Liberia | 1,073 | 643 | 1,708 | GBD, 2019 (43) |
| Libya | 1,692 | 1,034 | 2,657 | GBD, 2019 (43) |
| Madagascar | 2,333 | 1,545 | 3,424 | GBD, 2019 (43) |
| Malawi | 1,863 | 1,176 | 2,791 | GBD, 2019 (43) |
| Malaysia | 1,423 | 830 | 2,206 | GBD, 2019 (43) |
| Maldives | 1,457 | 893 | 2,192 | GBD, 2019 (43) |
| Mali | 895 | 520 | 1,416 | GBD, 2019 (43) |
| Marshall Islands | 1,326 | 821 | 2,010 | GBD, 2019 (43) |
| Mauritania | 1,479 | 849 | 2,422 | GBD, 2019 (43) |
| Mauritius | 1,484 | 930 | 2,264 | GBD, 2019 (43) |
| Mexico | 1,976 | 1,155 | 3,124 | GBD, 2019 (43) |
| Micronesia (Federated States of) | 1,455 | 886 | 2,258 | GBD, 2019 (43) |
| Mongolia | 928 | 518 | 1,527 | GBD, 2019 (43) |
| Montenegro | 1,449 | 842 | 2,291 | GBD, 2019 (43) |
| Morocco | 1,376 | 817 | 2,106 | GBD, 2019 (43) |
| Mozambique | 1,860 | 1,191 | 2,768 | GBD, 2019 (43) |
| Myanmar | 851 | 570 | 1,279 | GBD, 2019 (43) |
| Namibia | 653 | 383 | 1,064 | GBD, 2019 (43) |
| Nepal | 216 | 124 | 350 | GBD, 2019 (43) |
| Nicaragua | 2,426 | 1,516 | 3,634 | GBD, 2019 (43) |
| Niger | 1,127 | 721 | 1,802 | GBD, 2019 (43) |
| Nigeria | 1,748 | 1,044 | 2,762 | GBD, 2019 (43) |
| Pakistan | 425 | 248 | 699 | GBD, 2019 (43) |
| Panama | 2,842 | 1,768 | 4,243 | GBD, 2019 (43) |
| Papua New Guinea | 1,603 | 1,099 | 2,274 | GBD, 2019 (43) |
| Paraguay | 3,551 | 2,191 | 5,321 | GBD, 2019 (43) |
| Peru | 3,845 | 2,322 | 5,909 | GBD, 2019 (43) |
| Philippines | 2,834 | 1,864 | 4,245 | GBD, 2019 (43) |
| Republic of Moldova | 1,261 | 706 | 2,093 | GBD, 2019 (43) |
| Romania | 1,621 | 955 | 2,553 | GBD, 2019 (43) |
| Russian Federation | 1,321 | 706 | 2,192 | GBD, 2019 (43) |
| Rwanda | 3,419 | 2,261 | 5,121 | GBD, 2019 (43) |
| Saint Lucia | 3,921 | 2,493 | 5,805 | GBD, 2019 (43) |
| Saint Vincent and the Grenadines | 3,818 | 2,354 | 5,698 | GBD, 2019 (43) |
| Samoa | 1,409 | 827 | 2,201 | GBD, 2019 (43) |
| Sao Tome and Principe | 1,127 | 651 | 1,811 | GBD, 2019 (43) |
| Senegal | 908 | 533 | 1,447 | GBD, 2019 (43) |
| Serbia | 1,195 | 706 | 1,910 | GBD, 2019 (43) |
| Sierra Leone | 1,108 | 687 | 1,798 | GBD, 2019 (43) |
| Solomon Islands | 1,422 | 873 | 2,191 | GBD, 2019 (43) |
| Somalia | 1,878 | 1,240 | 2,807 | GBD, 2019 (43) |
| South Africa | 1,953 | 943 | 3,414 | GBD, 2019 (43) |
| South Sudan | 2,004 | 1,317 | 2,955 | GBD, 2019 (43) |
| Sri Lanka | 1,334 | 798 | 2,120 | GBD, 2019 (43) |
| Palestine | 1,650 | 971 | 2,599 | GBD, 2019 (43) |
| Sudan | 1,626 | 1,000 | 2,593 | GBD, 2019 (43) |
| Suriname | 3,708 | 2,320 | 5,539 | GBD, 2019 (43) |
| Eswatini | 948 | 552 | 1,574 | GBD, 2019 (43) |
| Syrian Arab Republic | 1,548 | 1,023 | 2,302 | GBD, 2019 (43) |
| Tajikistan | 938 | 514 | 1,549 | GBD, 2019 (43) |
| North Macedonia | 1,599 | 943 | 2,536 | GBD, 2019 (43) |
| Thailand | 1,713 | 1,020 | 2,647 | GBD, 2019 (43) |
| Timor-Leste | 1,538 | 1,049 | 2,227 | GBD, 2019 (43) |
| Togo | 1,265 | 749 | 2,101 | GBD, 2019 (43) |
| Tonga | 1,709 | 1,043 | 2,652 | GBD, 2019 (43) |
| Tunisia | 1,625 | 960 | 2,543 | GBD, 2019 (43) |
| Turkey | 1,836 | 1,112 | 2,750 | GBD, 2019 (43) |
| Turkmenistan | 959 | 540 | 1,588 | GBD, 2019 (43) |
| Tuvalu | 1,652 | 983 | 2,624 | GBD, 2019 (43) |
| Uganda | 2,296 | 1,480 | 3,509 | GBD, 2019 (43) |
| Ukraine | 1,770 | 958 | 2,947 | GBD, 2019 (43) |
| United Republic of Tanzania | 2,852 | 1,849 | 4,302 | GBD, 2019 (43) |
| Uzbekistan | 1,102 | 618 | 1,838 | GBD, 2019 (43) |
| Vanuatu | 1,380 | 793 | 2,195 | GBD, 2019 (43) |
| Venezuela (Bolivarian Republic of) | 2,371 | 1,425 | 3,570 | GBD, 2019 (43) |
| Vietnam | 1,347 | 807 | 2,080 | GBD, 2019 (43) |
| Yemen | 1,571 | 972 | 2,445 | GBD, 2019 (43) |
| Zambia | 1,284 | 802 | 1,987 | GBD, 2019 (43) |
| Zimbabwe | 542 | 320 | 870 | GBD, 2019 (43) |

Acronym: LI, low income; LMI, low-middle income; RSV, respiratory syncytial virus; UMI, upper middle income.

1. *Rate of RSV mortality, aged <5 years.*

RSV mortality rates, by income status (low income, lower middle income, upper middle income) were taken from Li 2022 (1) and adjusted into the standard format required by UNIVAC i.e. per 100,000 per year.

**Table S5.** RSV mortality rate, aged <5 years (per 100,000 children per year).

| **Country** | **Income** | **RSV mortality rate** | | | **Source** |
| --- | --- | --- | --- | --- | --- |
|  |  | **Mid** | **Low** | **High** |  |
| Afghanistan | LI | 29.86 | 23.89 | 37.32 | Li, 2022 (1) |
| Albania | UMI | 4.73 | 3.62 | 6.41 | Li, 2022 (1) |
| Algeria | UMI | 4.73 | 3.62 | 6.41 | Li, 2022 (1) |
| Angola | LMI | 19.76 | 14.82 | 26.68 | Li, 2022 (1) |
| Argentina | UMI | 4.73 | 3.62 | 6.41 | Li, 2022 (1) |
| Armenia | LMI | 19.76 | 14.82 | 26.68 | Li, 2022 (1) |
| Azerbaijan | UMI | 4.73 | 3.62 | 6.41 | Li, 2022 (1) |
| Bangladesh | LMI | 19.76 | 14.82 | 26.68 | Li, 2022 (1) |
| Belarus | UMI | 4.73 | 3.62 | 6.41 | Li, 2022 (1) |
| Belize | UMI | 4.73 | 3.62 | 6.41 | Li, 2022 (1) |
| Benin | LI | 29.86 | 23.89 | 37.32 | Li, 2022 (1) |
| Bhutan | LMI | 19.76 | 14.82 | 26.68 | Li, 2022 (1) |
| Bolivia (Plurinational State of) | LMI | 19.76 | 14.82 | 26.68 | Li, 2022 (1) |
| Bosnia and Herzegovina | UMI | 4.73 | 3.62 | 6.41 | Li, 2022 (1) |
| Botswana | UMI | 4.73 | 3.62 | 6.41 | Li, 2022 (1) |
| Brazil | UMI | 4.73 | 3.62 | 6.41 | Li, 2022 (1) |
| Bulgaria | UMI | 4.73 | 3.62 | 6.41 | Li, 2022 (1) |
| Burkina Faso | LI | 29.86 | 23.89 | 37.32 | Li, 2022 (1) |
| Burundi | LI | 29.86 | 23.89 | 37.32 | Li, 2022 (1) |
| Cabo Verde | LMI | 19.76 | 14.82 | 26.68 | Li, 2022 (1) |
| Cambodia | LMI | 19.76 | 14.82 | 26.68 | Li, 2022 (1) |
| Cameroon | LMI | 19.76 | 14.82 | 26.68 | Li, 2022 (1) |
| Central African Republic | LI | 29.86 | 23.89 | 37.32 | Li, 2022 (1) |
| Chad | LI | 29.86 | 23.89 | 37.32 | Li, 2022 (1) |
| China | UMI | 4.73 | 3.62 | 6.41 | Li, 2022 (1) |
| Colombia | UMI | 4.73 | 3.62 | 6.41 | Li, 2022 (1) |
| Comoros | LI | 29.86 | 23.89 | 37.32 | Li, 2022 (1) |
| Congo | LMI | 19.76 | 14.82 | 26.68 | Li, 2022 (1) |
| Costa Rica | UMI | 4.73 | 3.62 | 6.41 | Li, 2022 (1) |
| Côte d'Ivoire | LMI | 19.76 | 14.82 | 26.68 | Li, 2022 (1) |
| Croatia | UMI | 4.73 | 3.62 | 6.41 | Li, 2022 (1) |
| Cuba | UMI | 4.73 | 3.62 | 6.41 | Li, 2022 (1) |
| Dem. People's Republic of Korea | LI | 29.86 | 23.89 | 37.32 | Li, 2022 (1) |
| Democratic Republic of the Congo | LI | 29.86 | 23.89 | 37.32 | Li, 2022 (1) |
| Djibouti | LMI | 19.76 | 14.82 | 26.68 | Li, 2022 (1) |
| Dominican Republic | UMI | 4.73 | 3.62 | 6.41 | Li, 2022 (1) |
| Ecuador | UMI | 4.73 | 3.62 | 6.41 | Li, 2022 (1) |
| Egypt | LMI | 19.76 | 14.82 | 26.68 | Li, 2022 (1) |
| El Salvador | LMI | 19.76 | 14.82 | 26.68 | Li, 2022 (1) |
| Equatorial Guinea | UMI | 4.73 | 3.62 | 6.41 | Li, 2022 (1) |
| Eritrea | LI | 29.86 | 23.89 | 37.32 | Li, 2022 (1) |
| Ethiopia | LI | 29.86 | 23.89 | 37.32 | Li, 2022 (1) |
| Fiji | UMI | 4.73 | 3.62 | 6.41 | Li, 2022 (1) |
| Gabon | UMI | 4.73 | 3.62 | 6.41 | Li, 2022 (1) |
| Gambia | LI | 29.86 | 23.89 | 37.32 | Li, 2022 (1) |
| Georgia | LMI | 19.76 | 14.82 | 26.68 | Li, 2022 (1) |
| Ghana | LMI | 19.76 | 14.82 | 26.68 | Li, 2022 (1) |
| Grenada | UMI | 4.73 | 3.62 | 6.41 | Li, 2022 (1) |
| Guatemala | LMI | 19.76 | 14.82 | 26.68 | Li, 2022 (1) |
| Guinea | LI | 29.86 | 23.89 | 37.32 | Li, 2022 (1) |
| Guinea-Bissau | LI | 29.86 | 23.89 | 37.32 | Li, 2022 (1) |
| Guyana | UMI | 4.73 | 3.62 | 6.41 | Li, 2022 (1) |
| Haiti | LI | 29.86 | 23.89 | 37.32 | Li, 2022 (1) |
| Honduras | LMI | 19.76 | 14.82 | 26.68 | Li, 2022 (1) |
| India | LMI | 19.76 | 14.82 | 26.68 | Li, 2022 (1) |
| Indonesia | LMI | 19.76 | 14.82 | 26.68 | Li, 2022 (1) |
| Iran (Islamic Republic of) | UMI | 4.73 | 3.62 | 6.41 | Li, 2022 (1) |
| Iraq | UMI | 4.73 | 3.62 | 6.41 | Li, 2022 (1) |
| Jamaica | UMI | 4.73 | 3.62 | 6.41 | Li, 2022 (1) |
| Jordan | LMI | 19.76 | 14.82 | 26.68 | Li, 2022 (1) |
| Kazakhstan | UMI | 4.73 | 3.62 | 6.41 | Li, 2022 (1) |
| Kenya | LMI | 19.76 | 14.82 | 26.68 | Li, 2022 (1) |
| Kiribati | LMI | 19.76 | 14.82 | 26.68 | Li, 2022 (1) |
| Kosovo | LMI | 19.76 | 14.82 | 26.68 | Li, 2022 (1) |
| Kyrgyzstan | LMI | 19.76 | 14.82 | 26.68 | Li, 2022 (1) |
| Lao People's Democratic Republic | LMI | 19.76 | 14.82 | 26.68 | Li, 2022 (1) |
| Lebanon | UMI | 4.73 | 3.62 | 6.41 | Li, 2022 (1) |
| Lesotho | LMI | 19.76 | 14.82 | 26.68 | Li, 2022 (1) |
| Liberia | LI | 29.86 | 23.89 | 37.32 | Li, 2022 (1) |
| Libya | UMI | 4.73 | 3.62 | 6.41 | Li, 2022 (1) |
| Madagascar | LI | 29.86 | 23.89 | 37.32 | Li, 2022 (1) |
| Malawi | LI | 29.86 | 23.89 | 37.32 | Li, 2022 (1) |
| Malaysia | UMI | 4.73 | 3.62 | 6.41 | Li, 2022 (1) |
| Maldives | UMI | 4.73 | 3.62 | 6.41 | Li, 2022 (1) |
| Mali | LI | 29.86 | 23.89 | 37.32 | Li, 2022 (1) |
| Marshall Islands | UMI | 4.73 | 3.62 | 6.41 | Li, 2022 (1) |
| Mauritania | LMI | 19.76 | 14.82 | 26.68 | Li, 2022 (1) |
| Mauritius | UMI | 4.73 | 3.62 | 6.41 | Li, 2022 (1) |
| Mexico | UMI | 4.73 | 3.62 | 6.41 | Li, 2022 (1) |
| Micronesia (Fed. States of) | LMI | 19.76 | 14.82 | 26.68 | Li, 2022 (1) |
| Mongolia | LMI | 19.76 | 14.82 | 26.68 | Li, 2022 (1) |
| Montenegro | UMI | 4.73 | 3.62 | 6.41 | Li, 2022 (1) |
| Morocco | LMI | 19.76 | 14.82 | 26.68 | Li, 2022 (1) |
| Mozambique | LI | 29.86 | 23.89 | 37.32 | Li, 2022 (1) |
| Myanmar | LMI | 19.76 | 14.82 | 26.68 | Li, 2022 (1) |
| Namibia | UMI | 4.73 | 3.62 | 6.41 | Li, 2022 (1) |
| Nepal | LI | 29.86 | 23.89 | 37.32 | Li, 2022 (1) |
| Nicaragua | LMI | 19.76 | 14.82 | 26.68 | Li, 2022 (1) |
| Niger | LI | 29.86 | 23.89 | 37.32 | Li, 2022 (1) |
| Nigeria | LMI | 19.76 | 14.82 | 26.68 | Li, 2022 (1) |
| Pakistan | LMI | 19.76 | 14.82 | 26.68 | Li, 2022 (1) |
| State of Palestine | LMI | 19.76 | 14.82 | 26.68 | Li, 2022 (1) |
| Panama | UMI | 4.73 | 3.62 | 6.41 | Li, 2022 (1) |
| Papua New Guinea | LMI | 19.76 | 14.82 | 26.68 | Li, 2022 (1) |
| Paraguay | UMI | 4.73 | 3.62 | 6.41 | Li, 2022 (1) |
| Peru | UMI | 4.73 | 3.62 | 6.41 | Li, 2022 (1) |
| Philippines | LMI | 19.76 | 14.82 | 26.68 | Li, 2022 (1) |
| Republic of Moldova | LMI | 19.76 | 14.82 | 26.68 | Li, 2022 (1) |
| Romania | UMI | 4.73 | 3.62 | 6.41 | Li, 2022 (1) |
| Russian Federation | UMI | 4.73 | 3.62 | 6.41 | Li, 2022 (1) |
| Rwanda | LI | 29.86 | 23.89 | 37.32 | Li, 2022 (1) |
| Saint Lucia | UMI | 4.73 | 3.62 | 6.41 | Li, 2022 (1) |
| Saint Vincent and the Grenadines | UMI | 4.73 | 3.62 | 6.41 | Li, 2022 (1) |
| Samoa | UMI | 4.73 | 3.62 | 6.41 | Li, 2022 (1) |
| Sao Tome and Principe | LMI | 19.76 | 14.82 | 26.68 | Li, 2022 (1) |
| Senegal | LI | 29.86 | 23.89 | 37.32 | Li, 2022 (1) |
| Serbia | UMI | 4.73 | 3.62 | 6.41 | Li, 2022 (1) |
| Sierra Leone | LI | 29.86 | 23.89 | 37.32 | Li, 2022 (1) |
| Solomon Islands | LMI | 19.76 | 14.82 | 26.68 | Li, 2022 (1) |
| Somalia | LI | 29.86 | 23.89 | 37.32 | Li, 2022 (1) |
| South Africa | UMI | 4.73 | 3.62 | 6.41 | Li, 2022 (1) |
| South Sudan | LI | 29.86 | 23.89 | 37.32 | Li, 2022 (1) |
| Sri Lanka | LMI | 19.76 | 14.82 | 26.68 | Li, 2022 (1) |
| Sudan | LMI | 19.76 | 14.82 | 26.68 | Li, 2022 (1) |
| Suriname | UMI | 4.73 | 3.62 | 6.41 | Li, 2022 (1) |
| Eswatini | LMI | 19.76 | 14.82 | 26.68 | Li, 2022 (1) |
| Syrian Arab Republic | LMI | 19.76 | 14.82 | 26.68 | Li, 2022 (1) |
| Tajikistan | LMI | 19.76 | 14.82 | 26.68 | Li, 2022 (1) |
| North Macedonia | UMI | 4.73 | 3.62 | 6.41 | Li, 2022 (1) |
| Thailand | UMI | 4.73 | 3.62 | 6.41 | Li, 2022 (1) |
| Timor-Leste | LMI | 19.76 | 14.82 | 26.68 | Li, 2022 (1) |
| Togo | LI | 29.86 | 23.89 | 37.32 | Li, 2022 (1) |
| Tonga | UMI | 4.73 | 3.62 | 6.41 | Li, 2022 (1) |
| Tunisia | LMI | 19.76 | 14.82 | 26.68 | Li, 2022 (1) |
| Turkey | UMI | 4.73 | 3.62 | 6.41 | Li, 2022 (1) |
| Turkmenistan | UMI | 4.73 | 3.62 | 6.41 | Li, 2022 (1) |
| Tuvalu | UMI | 4.73 | 3.62 | 6.41 | Li, 2022 (1) |
| Uganda | LI | 29.86 | 23.89 | 37.32 | Li, 2022 (1) |
| Ukraine | LMI | 19.76 | 14.82 | 26.68 | Li, 2022 (1) |
| United Republic of Tanzania | LI | 29.86 | 23.89 | 37.32 | Li, 2022 (1) |
| Uzbekistan | LMI | 19.76 | 14.82 | 26.68 | Li, 2022 (1) |
| Vanuatu | LMI | 19.76 | 14.82 | 26.68 | Li, 2022 (1) |
| Venezuela (Bolivarian Republic of) | UMI | 4.73 | 3.62 | 6.41 | Li, 2022 (1) |
| Vietnam | LMI | 19.76 | 14.82 | 26.68 | Li, 2022 (1) |
| Yemen | LMI | 19.76 | 14.82 | 26.68 | Li, 2022 (1) |
| Zambia | LMI | 19.76 | 14.82 | 26.68 | Li, 2022 (1) |
| Zimbabwe | LI | 29.86 | 23.89 | 37.32 | Li, 2022 (1) |

Acronym: LI, low income; LMI, low-middle income; RSV, respiratory syncytial virus; UMI, upper middle income.

1. *Rate of RSV hospital admissions, aged <5 years*

Incidence rates for RSV-associated lower respiratory infection hospital admissions were extracted (1) by income status (low income, lower middle income, upper middle income) and converted into the standard format required by UNIVAC i.e. per 100,000 per year.

**Table S6.** Hospital admission rates, aged <5 years (per 100,000 children per year).

| **Country** | **Income** | **Hospital admission rate** | | | **Source** |
| --- | --- | --- | --- | --- | --- |
|  |  | **Mid** | **Low** | **High** |  |
| Afghanistan | LI | 350 | 200 | 630 | Li, 2022 (1) |
| Albania | UMI | 620 | 380 | 1,030 | Li, 2022 (1) |
| Algeria | UMI | 620 | 380 | 1,030 | Li, 2022 (1) |
| Angola | LMI | 620 | 400 | 940 | Li, 2022 (1) |
| Argentina | UMI | 620 | 380 | 1,030 | Li, 2022 (1) |
| Armenia | LMI | 620 | 400 | 940 | Li, 2022 (1) |
| Azerbaijan | UMI | 620 | 380 | 1,030 | Li, 2022 (1) |
| Bangladesh | LMI | 620 | 400 | 940 | Li, 2022 (1) |
| Belarus | UMI | 620 | 380 | 1,030 | Li, 2022 (1) |
| Belize | UMI | 620 | 380 | 1,030 | Li, 2022 (1) |
| Benin | LI | 350 | 200 | 630 | Li, 2022 (1) |
| Bhutan | LMI | 620 | 400 | 940 | Li, 2022 (1) |
| Bolivia (Plurinational State of) | LMI | 620 | 400 | 940 | Li, 2022 (1) |
| Bosnia and Herzegovina | UMI | 620 | 380 | 1,030 | Li, 2022 (1) |
| Botswana | UMI | 620 | 380 | 1,030 | Li, 2022 (1) |
| Brazil | UMI | 620 | 380 | 1,030 | Li, 2022 (1) |
| Bulgaria | UMI | 620 | 380 | 1,030 | Li, 2022 (1) |
| Burkina Faso | LI | 350 | 200 | 630 | Li, 2022 (1) |
| Burundi | LI | 350 | 200 | 630 | Li, 2022 (1) |
| Cabo Verde | LMI | 620 | 400 | 940 | Li, 2022 (1) |
| Cambodia | LMI | 620 | 400 | 940 | Li, 2022 (1) |
| Cameroon | LMI | 620 | 400 | 940 | Li, 2022 (1) |
| Central African Republic | LI | 350 | 200 | 630 | Li, 2022 (1) |
| Chad | LI | 350 | 200 | 630 | Li, 2022 (1) |
| China | UMI | 620 | 380 | 1,030 | Li, 2022 (1) |
| Colombia | UMI | 620 | 380 | 1,030 | Li, 2022 (1) |
| Comoros | LI | 350 | 200 | 630 | Li, 2022 (1) |
| Congo | LMI | 620 | 400 | 940 | Li, 2022 (1) |
| Costa Rica | UMI | 620 | 380 | 1,030 | Li, 2022 (1) |
| Côte d'Ivoire | LMI | 620 | 400 | 940 | Li, 2022 (1) |
| Croatia | UMI | 620 | 380 | 1,030 | Li, 2022 (1) |
| Cuba | UMI | 620 | 380 | 1,030 | Li, 2022 (1) |
| Dem. People's Republic of Korea | LI | 350 | 200 | 630 | Li, 2022 (1) |
| Democratic Republic of the Congo | LI | 350 | 200 | 630 | Li, 2022 (1) |
| Djibouti | LMI | 620 | 400 | 940 | Li, 2022 (1) |
| Dominican Republic | UMI | 620 | 380 | 1,030 | Li, 2022 (1) |
| Ecuador | UMI | 620 | 380 | 1,030 | Li, 2022 (1) |
| Egypt | LMI | 620 | 400 | 940 | Li, 2022 (1) |
| El Salvador | LMI | 620 | 400 | 940 | Li, 2022 (1) |
| Equatorial Guinea | UMI | 620 | 380 | 1,030 | Li, 2022 (1) |
| Eritrea | LI | 350 | 200 | 630 | Li, 2022 (1) |
| Ethiopia | LI | 350 | 200 | 630 | Li, 2022 (1) |
| Fiji | UMI | 620 | 380 | 1,030 | Li, 2022 (1) |
| Gabon | UMI | 620 | 380 | 1,030 | Li, 2022 (1) |
| Gambia | LI | 350 | 200 | 630 | Li, 2022 (1) |
| Georgia | LMI | 620 | 400 | 940 | Li, 2022 (1) |
| Ghana | LMI | 620 | 400 | 940 | Li, 2022 (1) |
| Grenada | UMI | 620 | 380 | 1,030 | Li, 2022 (1) |
| Guatemala | LMI | 620 | 400 | 940 | Li, 2022 (1) |
| Guinea | LI | 350 | 200 | 630 | Li, 2022 (1) |
| Guinea-Bissau | LI | 350 | 200 | 630 | Li, 2022 (1) |
| Guyana | UMI | 620 | 380 | 1,030 | Li, 2022 (1) |
| Haiti | LI | 350 | 200 | 630 | Li, 2022 (1) |
| Honduras | LMI | 620 | 400 | 940 | Li, 2022 (1) |
| India | LMI | 620 | 400 | 940 | Li, 2022 (1) |
| Indonesia | LMI | 620 | 400 | 940 | Li, 2022 (1) |
| Iran (Islamic Republic of) | UMI | 620 | 380 | 1,030 | Li, 2022 (1) |
| Iraq | UMI | 620 | 380 | 1,030 | Li, 2022 (1) |
| Jamaica | UMI | 620 | 380 | 1,030 | Li, 2022 (1) |
| Jordan | LMI | 620 | 400 | 940 | Li, 2022 (1) |
| Kazakhstan | UMI | 620 | 380 | 1,030 | Li, 2022 (1) |
| Kenya | LMI | 620 | 400 | 940 | Li, 2022 (1) |
| Kiribati | LMI | 620 | 400 | 940 | Li, 2022 (1) |
| Kosovo | LMI | 620 | 400 | 940 | Li, 2022 (1) |
| Kyrgyzstan | LMI | 620 | 400 | 940 | Li, 2022 (1) |
| Lao People's Democratic Republic | LMI | 620 | 400 | 940 | Li, 2022 (1) |
| Lebanon | UMI | 620 | 380 | 1,030 | Li, 2022 (1) |
| Lesotho | LMI | 620 | 400 | 940 | Li, 2022 (1) |
| Liberia | LI | 350 | 200 | 630 | Li, 2022 (1) |
| Libya | UMI | 620 | 380 | 1,030 | Li, 2022 (1) |
| Madagascar | LI | 350 | 200 | 630 | Li, 2022 (1) |
| Malawi | LI | 350 | 200 | 630 | Li, 2022 (1) |
| Malaysia | UMI | 620 | 380 | 1,030 | Li, 2022 (1) |
| Maldives | UMI | 620 | 380 | 1,030 | Li, 2022 (1) |
| Mali | LI | 350 | 200 | 630 | Li, 2022 (1) |
| Marshall Islands | UMI | 620 | 380 | 1,030 | Li, 2022 (1) |
| Mauritania | LMI | 620 | 400 | 940 | Li, 2022 (1) |
| Mauritius | UMI | 620 | 380 | 1,030 | Li, 2022 (1) |
| Mexico | UMI | 620 | 380 | 1,030 | Li, 2022 (1) |
| Micronesia (Fed. States of) | LMI | 620 | 400 | 940 | Li, 2022 (1) |
| Mongolia | LMI | 620 | 400 | 940 | Li, 2022 (1) |
| Montenegro | UMI | 620 | 380 | 1,030 | Li, 2022 (1) |
| Morocco | LMI | 620 | 400 | 940 | Li, 2022 (1) |
| Mozambique | LI | 350 | 200 | 630 | Li, 2022 (1) |
| Myanmar | LMI | 620 | 400 | 940 | Li, 2022 (1) |
| Namibia | UMI | 620 | 380 | 1,030 | Li, 2022 (1) |
| Nepal | LI | 350 | 200 | 630 | Li, 2022 (1) |
| Nicaragua | LMI | 620 | 400 | 940 | Li, 2022 (1) |
| Niger | LI | 350 | 200 | 630 | Li, 2022 (1) |
| Nigeria | LMI | 620 | 400 | 940 | Li, 2022 (1) |
| Pakistan | LMI | 620 | 400 | 940 | Li, 2022 (1) |
| State of Palestine | LMI | 620 | 400 | 940 | Li, 2022 (1) |
| Panama | UMI | 620 | 380 | 1,030 | Li, 2022 (1) |
| Papua New Guinea | LMI | 620 | 400 | 940 | Li, 2022 (1) |
| Paraguay | UMI | 620 | 380 | 1,030 | Li, 2022 (1) |
| Peru | UMI | 620 | 380 | 1,030 | Li, 2022 (1) |
| Philippines | LMI | 620 | 400 | 940 | Li, 2022 (1) |
| Republic of Moldova | LMI | 620 | 400 | 940 | Li, 2022 (1) |
| Romania | UMI | 620 | 380 | 1,030 | Li, 2022 (1) |
| Russian Federation | UMI | 620 | 380 | 1,030 | Li, 2022 (1) |
| Rwanda | LI | 350 | 200 | 630 | Li, 2022 (1) |
| Saint Lucia | UMI | 620 | 380 | 1,030 | Li, 2022 (1) |
| Saint Vincent and the Grenadines | UMI | 620 | 380 | 1,030 | Li, 2022 (1) |
| Samoa | UMI | 620 | 380 | 1,030 | Li, 2022 (1) |
| Sao Tome and Principe | LMI | 620 | 400 | 940 | Li, 2022 (1) |
| Senegal | LI | 350 | 200 | 630 | Li, 2022 (1) |
| Serbia | UMI | 620 | 380 | 1,030 | Li, 2022 (1) |
| Sierra Leone | LI | 350 | 200 | 630 | Li, 2022 (1) |
| Solomon Islands | LMI | 620 | 400 | 940 | Li, 2022 (1) |
| Somalia | LI | 350 | 200 | 630 | Li, 2022 (1) |
| South Africa | UMI | 620 | 380 | 1,030 | Li, 2022 (1) |
| South Sudan | LI | 350 | 200 | 630 | Li, 2022 (1) |
| Sri Lanka | LMI | 620 | 400 | 940 | Li, 2022 (1) |
| Sudan | LMI | 620 | 400 | 940 | Li, 2022 (1) |
| Suriname | UMI | 620 | 380 | 1,030 | Li, 2022 (1) |
| Eswatini | LMI | 620 | 400 | 940 | Li, 2022 (1) |
| Syrian Arab Republic | LMI | 620 | 400 | 940 | Li, 2022 (1) |
| Tajikistan | LMI | 620 | 400 | 940 | Li, 2022 (1) |
| North Macedonia | UMI | 620 | 380 | 1,030 | Li, 2022 (1) |
| Thailand | UMI | 620 | 380 | 1,030 | Li, 2022 (1) |
| Timor-Leste | LMI | 620 | 400 | 940 | Li, 2022 (1) |
| Togo | LI | 350 | 200 | 630 | Li, 2022 (1) |
| Tonga | UMI | 620 | 380 | 1,030 | Li, 2022 (1) |
| Tunisia | LMI | 620 | 400 | 940 | Li, 2022 (1) |
| Turkey | UMI | 620 | 380 | 1,030 | Li, 2022 (1) |
| Turkmenistan | UMI | 620 | 380 | 1,030 | Li, 2022 (1) |
| Tuvalu | UMI | 620 | 380 | 1,030 | Li, 2022 (1) |
| Uganda | LI | 350 | 200 | 630 | Li, 2022 (1) |
| Ukraine | LMI | 620 | 400 | 940 | Li, 2022 (1) |
| United Republic of Tanzania | LI | 350 | 200 | 630 | Li, 2022 (1) |
| Uzbekistan | LMI | 620 | 400 | 940 | Li, 2022 (1) |
| Vanuatu | LMI | 620 | 400 | 940 | Li, 2022 (1) |
| Venezuela (Bolivarian Republic of) | UMI | 620 | 380 | 1,030 | Li, 2022 (1) |
| Vietnam | LMI | 620 | 400 | 940 | Li, 2022 (1) |
| Yemen | LMI | 620 | 400 | 940 | Li, 2022 (1) |
| Zambia | LMI | 620 | 400 | 940 | Li, 2022 (1) |
| Zimbabwe | LI | 350 | 200 | 630 | Li, 2022 (1) |

Acronym: LI, low income; LMI, low-middle income; RSV, respiratory syncytial virus; UMI, upper middle income.

1. *Rate of RSV clinic visits for severe RSV disease, aged <5 years*

WHO provides information on the percentage of children aged <5 years with symptoms of pneumonia that were taken to a healthcare provider (44). First, we calculated the mean value for all countries with data in each income stratum. Second, we multiplied this treatment percentage by the incidence of severe RSV-ALRI cases (1) to generate the rate of clinic visits among severe RSV disease cases.

**Table S7.** Incidence of clinic visits for severe RSV, aged 5 years (per 100,000 children, per year).

| **Country** | **Income** | **Incidence of clinic visits** | | | **Source** |
| --- | --- | --- | --- | --- | --- |
|  |  | **Mid** | **Low** | **High** |  |
| Afghanistan | LI | 257 | 27 | 2,422 | Severe rate x 54% (WHO) (44) |
| Albania | UMI | 494 | 303 | 1,430 | Severe rate x 72% (WHO) (44) |
| Algeria | UMI | 494 | 303 | 1,430 | Severe rate x 72% (WHO) (44) |
| Angola | LMI | 925 | 528 | 1,598 | Severe rate x 66% (WHO) (44) |
| Argentina | UMI | 494 | 303 | 1,430 | Severe rate x 72% (WHO) (44) |
| Armenia | LMI | 925 | 528 | 1,598 | Severe rate x 66% (WHO) (44) |
| Azerbaijan | UMI | 494 | 303 | 1,430 | Severe rate x 72% (WHO) (44) |
| Bangladesh | LMI | 925 | 528 | 1,598 | Severe rate x 66% (WHO) (44) |
| Belarus | UMI | 494 | 303 | 1,430 | Severe rate x 72% (WHO) (44) |
| Belize | UMI | 494 | 303 | 1,430 | Severe rate x 72% (WHO) (44) |
| Benin | LI | 257 | 27 | 2,422 | Severe rate x 54% (WHO) (44) |
| Bhutan | LMI | 925 | 528 | 1,598 | Severe rate x 66% (WHO) (44) |
| Bolivia (Plurinational State of) | LMI | 925 | 528 | 1,598 | Severe rate x 66% (WHO) (44) |
| Bosnia and Herzegovina | UMI | 494 | 303 | 1,430 | Severe rate x 72% (WHO) (44) |
| Botswana | UMI | 494 | 303 | 1,430 | Severe rate x 72% (WHO) (44) |
| Brazil | UMI | 494 | 303 | 1,430 | Severe rate x 72% (WHO) (44) |
| Bulgaria | UMI | 494 | 303 | 1,430 | Severe rate x 72% (WHO) (44) |
| Burkina Faso | LI | 257 | 27 | 2,422 | Severe rate x 54% (WHO) (44) |
| Burundi | LI | 257 | 27 | 2,422 | Severe rate x 54% (WHO) (44) |
| Cabo Verde | LMI | 925 | 528 | 1,598 | Severe rate x 66% (WHO) (44) |
| Cambodia | LMI | 925 | 528 | 1,598 | Severe rate x 66% (WHO) (44) |
| Cameroon | LMI | 925 | 528 | 1,598 | Severe rate x 66% (WHO) (44) |
| Central African Republic | LI | 257 | 27 | 2,422 | Severe rate x 54% (WHO) (44) |
| Chad | LI | 257 | 27 | 2,422 | Severe rate x 54% (WHO) (44) |
| China | UMI | 494 | 303 | 1,430 | Severe rate x 72% (WHO) (44) |
| Colombia | UMI | 494 | 303 | 1,430 | Severe rate x 72% (WHO) (44) |
| Comoros | LI | 257 | 27 | 2,422 | Severe rate x 54% (WHO) (44) |
| Congo | LMI | 925 | 528 | 1,598 | Severe rate x 66% (WHO) (44) |
| Costa Rica | UMI | 494 | 303 | 1,430 | Severe rate x 72% (WHO) (44) |
| Côte d'Ivoire | LMI | 925 | 528 | 1,598 | Severe rate x 66% (WHO) (44) |
| Croatia | UMI | 494 | 303 | 1,430 | Severe rate x 72% (WHO) (44) |
| Cuba | UMI | 494 | 303 | 1,430 | Severe rate x 72% (WHO) (44) |
| Dem. People's Republic of Korea | LI | 257 | 27 | 2,422 | Severe rate x 54% (WHO) (44) |
| Democratic Republic of the Congo | LI | 257 | 27 | 2,422 | Severe rate x 54% (WHO) (44) |
| Djibouti | LMI | 925 | 528 | 1,598 | Severe rate x 66% (WHO) (44) |
| Dominican Republic | UMI | 494 | 303 | 1,430 | Severe rate x 72% (WHO) (44) |
| Ecuador | UMI | 494 | 303 | 1,430 | Severe rate x 72% (WHO) (44) |
| Egypt | LMI | 925 | 528 | 1,598 | Severe rate x 66% (WHO) (44) |
| El Salvador | LMI | 925 | 528 | 1,598 | Severe rate x 66% (WHO) (44) |
| Equatorial Guinea | UMI | 494 | 303 | 1,430 | Severe rate x 72% (WHO) (44) |
| Eritrea | LI | 257 | 27 | 2,422 | Severe rate x 54% (WHO) (44) |
| Ethiopia | LI | 257 | 27 | 2,422 | Severe rate x 54% (WHO) (44) |
| Fiji | UMI | 494 | 303 | 1,430 | Severe rate x 72% (WHO) (44) |
| Gabon | UMI | 494 | 303 | 1,430 | Severe rate x 72% (WHO) (44) |
| Gambia | LI | 257 | 27 | 2,422 | Severe rate x 54% (WHO) (44) |
| Georgia | LMI | 925 | 528 | 1,598 | Severe rate x 66% (WHO) (44) |
| Ghana | LMI | 925 | 528 | 1,598 | Severe rate x 66% (WHO) (44) |
| Grenada | UMI | 494 | 303 | 1,430 | Severe rate x 72% (WHO) (44) |
| Guatemala | LMI | 925 | 528 | 1,598 | Severe rate x 66% (WHO) (44) |
| Guinea | LI | 257 | 27 | 2,422 | Severe rate x 54% (WHO) (44) |
| Guinea-Bissau | LI | 257 | 27 | 2,422 | Severe rate x 54% (WHO) (44) |
| Guyana | UMI | 494 | 303 | 1,430 | Severe rate x 72% (WHO) (44) |
| Haiti | LI | 257 | 27 | 2,422 | Severe rate x 54% (WHO) (44) |
| Honduras | LMI | 925 | 528 | 1,598 | Severe rate x 66% (WHO) (44) |
| India | LMI | 925 | 528 | 1,598 | Severe rate x 66% (WHO) (44) |
| Indonesia | LMI | 925 | 528 | 1,598 | Severe rate x 66% (WHO) (44) |
| Iran (Islamic Republic of) | UMI | 494 | 303 | 1,430 | Severe rate x 72% (WHO) (44) |
| Iraq | UMI | 494 | 303 | 1,430 | Severe rate x 72% (WHO) (44) |
| Jamaica | UMI | 494 | 303 | 1,430 | Severe rate x 72% (WHO) (44) |
| Jordan | LMI | 925 | 528 | 1,598 | Severe rate x 66% (WHO) (44) |
| Kazakhstan | UMI | 494 | 303 | 1,430 | Severe rate x 72% (WHO) (44) |
| Kenya | LMI | 925 | 528 | 1,598 | Severe rate x 66% (WHO) (44) |
| Kiribati | LMI | 925 | 528 | 1,598 | Severe rate x 66% (WHO) (44) |
| Kosovo | LMI | 925 | 528 | 1,598 | Severe rate x 66% (WHO) (44) |
| Kyrgyzstan | LMI | 925 | 528 | 1,598 | Severe rate x 66% (WHO) (44) |
| Lao People's Democratic Republic | LMI | 925 | 528 | 1,598 | Severe rate x 66% (WHO) (44) |
| Lebanon | UMI | 494 | 303 | 1,430 | Severe rate x 72% (WHO) (44) |
| Lesotho | LMI | 925 | 528 | 1,598 | Severe rate x 66% (WHO) (44) |
| Liberia | LI | 257 | 27 | 2,422 | Severe rate x 54% (WHO) (44) |
| Libya | UMI | 494 | 303 | 1,430 | Severe rate x 72% (WHO) (44) |
| Madagascar | LI | 257 | 27 | 2,422 | Severe rate x 54% (WHO) (44) |
| Malawi | LI | 257 | 27 | 2,422 | Severe rate x 54% (WHO) (44) |
| Malaysia | UMI | 494 | 303 | 1,430 | Severe rate x 72% (WHO) (44) |
| Maldives | UMI | 494 | 303 | 1,430 | Severe rate x 72% (WHO) (44) |
| Mali | LI | 257 | 27 | 2,422 | Severe rate x 54% (WHO) (44) |
| Marshall Islands | UMI | 494 | 303 | 1,430 | Severe rate x 72% (WHO) (44) |
| Mauritania | LMI | 925 | 528 | 1,598 | Severe rate x 66% (WHO) (44) |
| Mauritius | UMI | 494 | 303 | 1,430 | Severe rate x 72% (WHO) (44) |
| Mexico | UMI | 494 | 303 | 1,430 | Severe rate x 72% (WHO) (44) |
| Micronesia (Fed. States of) | LMI | 925 | 528 | 1,598 | Severe rate x 66% (WHO) (44) |
| Mongolia | LMI | 925 | 528 | 1,598 | Severe rate x 66% (WHO) (44) |
| Montenegro | UMI | 494 | 303 | 1,430 | Severe rate x 72% (WHO) (44) |
| Morocco | LMI | 925 | 528 | 1,598 | Severe rate x 66% (WHO) (44) |
| Mozambique | LI | 257 | 27 | 2,422 | Severe rate x 54% (WHO) (44) |
| Myanmar | LMI | 925 | 528 | 1,598 | Severe rate x 66% (WHO) (44) |
| Namibia | UMI | 494 | 303 | 1,430 | Severe rate x 72% (WHO) (44) |
| Nepal | LI | 257 | 27 | 2,422 | Severe rate x 54% (WHO) (44) |
| Nicaragua | LMI | 925 | 528 | 1,598 | Severe rate x 66% (WHO) (44) |
| Niger | LI | 257 | 27 | 2,422 | Severe rate x 54% (WHO) (44) |
| Nigeria | LMI | 925 | 528 | 1,598 | Severe rate x 66% (WHO) (44) |
| Pakistan | LMI | 925 | 528 | 1,598 | Severe rate x 66% (WHO) (44) |
| State of Palestine | LMI | 494 | 303 | 1,430 | Severe rate x 66% (WHO) (44) |
| Panama | UMI | 925 | 528 | 1,598 | Severe rate x 72% (WHO) (44) |
| Papua New Guinea | LMI | 494 | 303 | 1,430 | Severe rate x 66% (WHO) (44) |
| Paraguay | UMI | 494 | 303 | 1,430 | Severe rate x 72% (WHO) (44) |
| Peru | UMI | 925 | 528 | 1,598 | Severe rate x 72% (WHO) (44) |
| Philippines | LMI | 925 | 528 | 1,598 | Severe rate x 66% (WHO) (44) |
| Republic of Moldova | LMI | 494 | 303 | 1,430 | Severe rate x 66% (WHO) (44) |
| Romania | UMI | 494 | 303 | 1,430 | Severe rate x 72% (WHO) (44) |
| Russian Federation | UMI | 257 | 27 | 2,422 | Severe rate x 72% (WHO) (44) |
| Rwanda | LI | 494 | 303 | 1,430 | Severe rate x 54% (WHO) (44) |
| Saint Lucia | UMI | 494 | 303 | 1,430 | Severe rate x 72% (WHO) (44) |
| Saint Vincent and the Grenadines | UMI | 494 | 303 | 1,430 | Severe rate x 72% (WHO) (44) |
| Samoa | UMI | 925 | 528 | 1,598 | Severe rate x 72% (WHO) (44) |
| Sao Tome and Principe | LMI | 257 | 27 | 2,422 | Severe rate x 66% (WHO) (44) |
| Senegal | LI | 494 | 303 | 1,430 | Severe rate x 54% (WHO) (44) |
| Serbia | UMI | 257 | 27 | 2,422 | Severe rate x 72% (WHO) (44) |
| Sierra Leone | LI | 925 | 528 | 1,598 | Severe rate x 54% (WHO) (44) |
| Solomon Islands | LMI | 257 | 27 | 2,422 | Severe rate x 66% (WHO) (44) |
| Somalia | LI | 494 | 303 | 1,430 | Severe rate x 54% (WHO) (44) |
| South Africa | UMI | 257 | 27 | 2,422 | Severe rate x 72% (WHO) (44) |
| South Sudan | LI | 925 | 528 | 1,598 | Severe rate x 54% (WHO) (44) |
| Sri Lanka | LMI | 925 | 528 | 1,598 | Severe rate x 66% (WHO) (44) |
| Sudan | LMI | 925 | 528 | 1,598 | Severe rate x 66% (WHO) (44) |
| Suriname | UMI | 494 | 303 | 1,430 | Severe rate x 72% (WHO) (44) |
| Eswatini | LMI | 925 | 528 | 1,598 | Severe rate x 66% (WHO) (44) |
| Syrian Arab Republic | LMI | 925 | 528 | 1,598 | Severe rate x 66% (WHO) (44) |
| Tajikistan | LMI | 925 | 528 | 1,598 | Severe rate x 66% (WHO) (44) |
| North Macedonia | UMI | 494 | 303 | 1,430 | Severe rate x 72% (WHO) (44) |
| Thailand | UMI | 494 | 303 | 1,430 | Severe rate x 72% (WHO) (44) |
| Timor-Leste | LMI | 925 | 528 | 1,598 | Severe rate x 66% (WHO) (44) |
| Togo | LI | 257 | 27 | 2,422 | Severe rate x 54% (WHO) (44) |
| Tonga | UMI | 494 | 303 | 1,430 | Severe rate x 72% (WHO) (44) |
| Tunisia | LMI | 925 | 528 | 1,598 | Severe rate x 66% (WHO) (44) |
| Turkey | UMI | 494 | 303 | 1,430 | Severe rate x 72% (WHO) (44) |
| Turkmenistan | UMI | 494 | 303 | 1,430 | Severe rate x 72% (WHO) (44) |
| Tuvalu | UMI | 494 | 303 | 1,430 | Severe rate x 72% (WHO) (44) |
| Uganda | LI | 257 | 27 | 2,422 | Severe rate x 54% (WHO) (44) |
| Ukraine | LMI | 925 | 528 | 1,598 | Severe rate x 66% (WHO) (44) |
| United Republic of Tanzania | LI | 257 | 27 | 2,422 | Severe rate x 54% (WHO) (44) |
| Uzbekistan | LMI | 925 | 528 | 1,598 | Severe rate x 66% (WHO) (44) |
| Vanuatu | LMI | 925 | 528 | 1,598 | Severe rate x 66% (WHO) (44) |
| Venezuela (Bolivarian Republic of) | UMI | 494 | 303 | 1,430 | Severe rate x 72% (WHO) (44) |
| Vietnam | LMI | 925 | 528 | 1,598 | Severe rate x 66% (WHO) (44) |
| Yemen | LMI | 925 | 528 | 1,598 | Severe rate x 66% (WHO) (44) |
| Zambia | LMI | 925 | 528 | 1,598 | Severe rate x 66% (WHO) (44) |
| Zimbabwe | LI | 257 | 27 | 2,422 | Severe rate x 54% (WHO) (44) |

Acronym: LI, low income; LMI, low-middle income; RSV, respiratory syncytial virus; UMI, upper middle income.

1. *Rate of RSV clinic visits for non-severe RSV disease, aged <5 years*

As described above, we calculated the mean percentage of children aged <5 years with symptoms of pneumonia that were taken to a healthcare provider, by income stratum (44), and multiplied this by the incidence of non-severe RSV-ALRI cases (1) to generate the rate of clinic visits among non-severe RSV disease cases.

**Table S8.** Incidence of clinic visits for non-severe RSV, aged 5 years (per 100,000 children, per year).

| **Country** | **Income** | **Incidence of clinic visits** | | | **Source** |
| --- | --- | --- | --- | --- | --- |
|  |  | **Mid** | **Low** | **High** |  |
| Afghanistan | LI | 2,385 | 1,422 | 4,005 | Non-severe rate x 54% (WHO) (44) |
| Albania | UMI | 3,455 | 1,590 | 7,506 | Non-severe rate x 72% (WHO) (44) |
| Algeria | UMI | 3,455 | 1,590 | 7,506 | Non-severe rate x 72% (WHO) (44) |
| Angola | LMI | 2,470 | 1,816 | 3,354 | Non-severe rate x 66% (WHO) (44) |
| Argentina | UMI | 3,455 | 1,590 | 7,506 | Non-severe rate x 72% (WHO) (44) |
| Armenia | LMI | 2,470 | 1,816 | 3,354 | Non-severe rate x 66% (WHO) (44) |
| Azerbaijan | UMI | 3,455 | 1,590 | 7,506 | Non-severe rate x 72% (WHO) (44) |
| Bangladesh | LMI | 2,470 | 1,816 | 3,354 | Non-severe rate x 66% (WHO) (44) |
| Belarus | UMI | 3,455 | 1,590 | 7,506 | Non-severe rate x 72% (WHO) (44) |
| Belize | UMI | 3,455 | 1,590 | 7,506 | Non-severe rate x 72% (WHO) (44) |
| Benin | LI | 2,385 | 1,422 | 4,005 | Non-severe rate x 54% (WHO) (44) |
| Bhutan | LMI | 2,470 | 1,816 | 3,354 | Non-severe rate x 66% (WHO) (44) |
| Bolivia (Plurinational State of) | LMI | 2,470 | 1,816 | 3,354 | Non-severe rate x 66% (WHO) (44) |
| Bosnia and Herzegovina | UMI | 3,455 | 1,590 | 7,506 | Non-severe rate x 72% (WHO) (44) |
| Botswana | UMI | 3,455 | 1,590 | 7,506 | Non-severe rate x 72% (WHO) (44) |
| Brazil | UMI | 3,455 | 1,590 | 7,506 | Non-severe rate x 72% (WHO) (44) |
| Bulgaria | UMI | 3,455 | 1,590 | 7,506 | Non-severe rate x 72% (WHO) (44) |
| Burkina Faso | LI | 2,385 | 1,422 | 4,005 | Non-severe rate x 54% (WHO) (44) |
| Burundi | LI | 2,385 | 1,422 | 4,005 | Non-severe rate x 54% (WHO) (44) |
| Cabo Verde | LMI | 2,470 | 1,816 | 3,354 | Non-severe rate x 66% (WHO) (44) |
| Cambodia | LMI | 2,470 | 1,816 | 3,354 | Non-severe rate x 66% (WHO) (44) |
| Cameroon | LMI | 2,470 | 1,816 | 3,354 | Non-severe rate x 66% (WHO) (44) |
| Central African Republic | LI | 2,385 | 1,422 | 4,005 | Non-severe rate x 54% (WHO) (44) |
| Chad | LI | 2,385 | 1,422 | 4,005 | Non-severe rate x 54% (WHO) (44) |
| China | UMI | 3,455 | 1,590 | 7,506 | Non-severe rate x 72% (WHO) (44) |
| Colombia | UMI | 3,455 | 1,590 | 7,506 | Non-severe rate x 72% (WHO) (44) |
| Comoros | LI | 2,385 | 1,422 | 4,005 | Non-severe rate x 54% (WHO) (44) |
| Congo | LMI | 2,470 | 1,816 | 3,354 | Non-severe rate x 66% (WHO) (44) |
| Costa Rica | UMI | 3,455 | 1,590 | 7,506 | Non-severe rate x 72% (WHO) (44) |
| Côte d'Ivoire | LMI | 2,470 | 1,816 | 3,354 | Non-severe rate x 66% (WHO) (44) |
| Croatia | UMI | 3,455 | 1,590 | 7,506 | Non-severe rate x 72% (WHO) (44) |
| Cuba | UMI | 3,455 | 1,590 | 7,506 | Non-severe rate x 72% (WHO) (44) |
| Dem. People's Republic of Korea | LI | 2,385 | 1,422 | 4,005 | Non-severe rate x 54% (WHO) (44) |
| Democratic Republic of the Congo | LI | 2,385 | 1,422 | 4,005 | Non-severe rate x 54% (WHO) (44) |
| Djibouti | LMI | 2,470 | 1,816 | 3,354 | Non-severe rate x 66% (WHO) (44) |
| Dominican Republic | UMI | 3,455 | 1,590 | 7,506 | Non-severe rate x 72% (WHO) (44) |
| Ecuador | UMI | 3,455 | 1,590 | 7,506 | Non-severe rate x 72% (WHO) (44) |
| Egypt | LMI | 2,470 | 1,816 | 3,354 | Non-severe rate x 66% (WHO) (44) |
| El Salvador | LMI | 2,470 | 1,816 | 3,354 | Non-severe rate x 66% (WHO) (44) |
| Equatorial Guinea | UMI | 3,455 | 1,590 | 7,506 | Non-severe rate x 72% (WHO) (44) |
| Eritrea | LI | 2,385 | 1,422 | 4,005 | Non-severe rate x 54% (WHO) (44) |
| Ethiopia | LI | 2,385 | 1,422 | 4,005 | Non-severe rate x 54% (WHO) (44) |
| Fiji | UMI | 3,455 | 1,590 | 7,506 | Non-severe rate x 72% (WHO) (44) |
| Gabon | UMI | 3,455 | 1,590 | 7,506 | Non-severe rate x 72% (WHO) (44) |
| Gambia | LI | 2,385 | 1,422 | 4,005 | Non-severe rate x 54% (WHO) (44) |
| Georgia | LMI | 2,470 | 1,816 | 3,354 | Non-severe rate x 66% (WHO) (44) |
| Ghana | LMI | 2,470 | 1,816 | 3,354 | Non-severe rate x 66% (WHO) (44) |
| Grenada | UMI | 3,455 | 1,590 | 7,506 | Non-severe rate x 72% (WHO) (44) |
| Guatemala | LMI | 2,470 | 1,816 | 3,354 | Non-severe rate x 66% (WHO) (44) |
| Guinea | LI | 2,385 | 1,422 | 4,005 | Non-severe rate x 54% (WHO) (44) |
| Guinea-Bissau | LI | 2,385 | 1,422 | 4,005 | Non-severe rate x 54% (WHO) (44) |
| Guyana | UMI | 3,455 | 1,590 | 7,506 | Non-severe rate x 72% (WHO) (44) |
| Haiti | LI | 2,385 | 1,422 | 4,005 | Non-severe rate x 54% (WHO) (44) |
| Honduras | LMI | 2,470 | 1,816 | 3,354 | Non-severe rate x 66% (WHO) (44) |
| India | LMI | 2,470 | 1,816 | 3,354 | Non-severe rate x 66% (WHO) (44) |
| Indonesia | LMI | 2,470 | 1,816 | 3,354 | Non-severe rate x 66% (WHO) (44) |
| Iran (Islamic Republic of) | UMI | 3,455 | 1,590 | 7,506 | Non-severe rate x 72% (WHO) (44) |
| Iraq | UMI | 3,455 | 1,590 | 7,506 | Non-severe rate x 72% (WHO) (44) |
| Jamaica | UMI | 3,455 | 1,590 | 7,506 | Non-severe rate x 72% (WHO) (44) |
| Jordan | LMI | 2,470 | 1,816 | 3,354 | Non-severe rate x 66% (WHO) (44) |
| Kazakhstan | UMI | 3,455 | 1,590 | 7,506 | Non-severe rate x 72% (WHO) (44) |
| Kenya | LMI | 2,470 | 1,816 | 3,354 | Non-severe rate x 66% (WHO) (44) |
| Kiribati | LMI | 2,470 | 1,816 | 3,354 | Non-severe rate x 66% (WHO) (44) |
| Kosovo | LMI | 2,470 | 1,816 | 3,354 | Non-severe rate x 66% (WHO) (44) |
| Kyrgyzstan | LMI | 2,470 | 1,816 | 3,354 | Non-severe rate x 66% (WHO) (44) |
| Lao People's Democratic Republic | LMI | 2,470 | 1,816 | 3,354 | Non-severe rate x 66% (WHO) (44) |
| Lebanon | UMI | 3,455 | 1,590 | 7,506 | Non-severe rate x 72% (WHO) (44) |
| Lesotho | LMI | 2,470 | 1,816 | 3,354 | Non-severe rate x 66% (WHO) (44) |
| Liberia | LI | 2,385 | 1,422 | 4,005 | Non-severe rate x 54% (WHO) (44) |
| Libya | UMI | 3,455 | 1,590 | 7,506 | Non-severe rate x 72% (WHO) (44) |
| Madagascar | LI | 2,385 | 1,422 | 4,005 | Non-severe rate x 54% (WHO) (44) |
| Malawi | LI | 2,385 | 1,422 | 4,005 | Non-severe rate x 54% (WHO) (44) |
| Malaysia | UMI | 3,455 | 1,590 | 7,506 | Non-severe rate x 72% (WHO) (44) |
| Maldives | UMI | 3,455 | 1,590 | 7,506 | Non-severe rate x 72% (WHO) (44) |
| Mali | LI | 2,385 | 1,422 | 4,005 | Non-severe rate x 54% (WHO) (44) |
| Marshall Islands | UMI | 3,455 | 1,590 | 7,506 | Non-severe rate x 72% (WHO) (44) |
| Mauritania | LMI | 2,470 | 1,816 | 3,354 | Non-severe rate x 66% (WHO) (44) |
| Mauritius | UMI | 3,455 | 1,590 | 7,506 | Non-severe rate x 72% (WHO) (44) |
| Mexico | UMI | 3,455 | 1,590 | 7,506 | Non-severe rate x 72% (WHO) (44) |
| Micronesia (Fed. States of) | LMI | 2,470 | 1,816 | 3,354 | Non-severe rate x 66% (WHO) (44) |
| Mongolia | LMI | 2,470 | 1,816 | 3,354 | Non-severe rate x 66% (WHO) (44) |
| Montenegro | UMI | 3,455 | 1,590 | 7,506 | Non-severe rate x 72% (WHO) (44) |
| Morocco | LMI | 2,470 | 1,816 | 3,354 | Non-severe rate x 66% (WHO) (44) |
| Mozambique | LI | 2,385 | 1,422 | 4,005 | Non-severe rate x 54% (WHO) (44) |
| Myanmar | LMI | 2,470 | 1,816 | 3,354 | Non-severe rate x 66% (WHO) (44) |
| Namibia | UMI | 3,455 | 1,590 | 7,506 | Non-severe rate x 72% (WHO) (44) |
| Nepal | LI | 2,385 | 1,422 | 4,005 | Non-severe rate x 54% (WHO) (44) |
| Nicaragua | LMI | 2,470 | 1,816 | 3,354 | Non-severe rate x 66% (WHO) (44) |
| Niger | LI | 2,385 | 1,422 | 4,005 | Non-severe rate x 54% (WHO) (44) |
| Nigeria | LMI | 2,470 | 1,816 | 3,354 | Non-severe rate x 66% (WHO) (44) |
| Pakistan | LMI | 2,470 | 1,816 | 3,354 | Non-severe rate x 66% (WHO) (44) |
| State of Palestine | LMI | 2,470 | 1,816 | 3,354 | Non-severe rate x 66% (WHO) (44) |
| Panama | UMI | 3,455 | 1,590 | 7,506 | Non-severe rate x 72% (WHO) (44) |
| Papua New Guinea | LMI | 2,470 | 1,816 | 3,354 | Non-severe rate x 66% (WHO) (44) |
| Paraguay | UMI | 3,455 | 1,590 | 7,506 | Non-severe rate x 72% (WHO) (44) |
| Peru | UMI | 3,455 | 1,590 | 7,506 | Non-severe rate x 72% (WHO) (44) |
| Philippines | LMI | 2,470 | 1,816 | 3,354 | Non-severe rate x 66% (WHO) (44) |
| Republic of Moldova | LMI | 2,470 | 1,816 | 3,354 | Non-severe rate x 66% (WHO) (44) |
| Romania | UMI | 3,455 | 1,590 | 7,506 | Non-severe rate x 72% (WHO) (44) |
| Russian Federation | UMI | 3,455 | 1,590 | 7,506 | Non-severe rate x 72% (WHO) (44) |
| Rwanda | LI | 2,385 | 1,422 | 4,005 | Non-severe rate x 54% (WHO) (44) |
| Saint Lucia | UMI | 3,455 | 1,590 | 7,506 | Non-severe rate x 72% (WHO) (44) |
| Saint Vincent and the Grenadines | UMI | 3,455 | 1,590 | 7,506 | Non-severe rate x 72% (WHO) (44) |
| Samoa | UMI | 3,455 | 1,590 | 7,506 | Non-severe rate x 72% (WHO) (44) |
| Sao Tome and Principe | LMI | 2,470 | 1,816 | 3,354 | Non-severe rate x 66% (WHO) (44) |
| Senegal | LI | 2,385 | 1,422 | 4,005 | Non-severe rate x 54% (WHO) (44) |
| Serbia | UMI | 3,455 | 1,590 | 7,506 | Non-severe rate x 72% (WHO) (44) |
| Sierra Leone | LI | 2,385 | 1,422 | 4,005 | Non-severe rate x 54% (WHO) (44) |
| Solomon Islands | LMI | 2,470 | 1,816 | 3,354 | Non-severe rate x 66% (WHO) (44) |
| Somalia | LI | 2,385 | 1,422 | 4,005 | Non-severe rate x 54% (WHO) (44) |
| South Africa | UMI | 3,455 | 1,590 | 7,506 | Non-severe rate x 72% (WHO) (44) |
| South Sudan | LI | 2,385 | 1,422 | 4,005 | Non-severe rate x 54% (WHO) (44) |
| Sri Lanka | LMI | 2,470 | 1,816 | 3,354 | Non-severe rate x 66% (WHO) (44) |
| Sudan | LMI | 2,470 | 1,816 | 3,354 | Non-severe rate x 66% (WHO) (44) |
| Suriname | UMI | 3,455 | 1,590 | 7,506 | Non-severe rate x 72% (WHO) (44) |
| Eswatini | LMI | 2,470 | 1,816 | 3,354 | Non-severe rate x 66% (WHO) (44) |
| Syrian Arab Republic | LMI | 2,470 | 1,816 | 3,354 | Non-severe rate x 66% (WHO) (44) |
| Tajikistan | LMI | 2,470 | 1,816 | 3,354 | Non-severe rate x 66% (WHO) (44) |
| North Macedonia | UMI | 3,455 | 1,590 | 7,506 | Non-severe rate x 72% (WHO) (44) |
| Thailand | UMI | 3,455 | 1,590 | 7,506 | Non-severe rate x 72% (WHO) (44) |
| Timor-Leste | LMI | 2,470 | 1,816 | 3,354 | Non-severe rate x 66% (WHO) (44) |
| Togo | LI | 2,385 | 1,422 | 4,005 | Non-severe rate x 54% (WHO) (44) |
| Tonga | UMI | 3,455 | 1,590 | 7,506 | Non-severe rate x 72% (WHO) (44) |
| Tunisia | LMI | 2,470 | 1,816 | 3,354 | Non-severe rate x 66% (WHO) (44) |
| Turkey | UMI | 3,455 | 1,590 | 7,506 | Non-severe rate x 72% (WHO) (44) |
| Turkmenistan | UMI | 3,455 | 1,590 | 7,506 | Non-severe rate x 72% (WHO) (44) |
| Tuvalu | UMI | 3,455 | 1,590 | 7,506 | Non-severe rate x 72% (WHO) (44) |
| Uganda | LI | 2,385 | 1,422 | 4,005 | Non-severe rate x 54% (WHO) (44) |
| Ukraine | LMI | 2,470 | 1,816 | 3,354 | Non-severe rate x 66% (WHO) (44) |
| United Republic of Tanzania | LI | 2,385 | 1,422 | 4,005 | Non-severe rate x 54% (WHO) (44) |
| Uzbekistan | LMI | 2,470 | 1,816 | 3,354 | Non-severe rate x 66% (WHO) (44) |
| Vanuatu | LMI | 2,470 | 1,816 | 3,354 | Non-severe rate x 66% (WHO) (44) |
| Venezuela (Bolivarian Republic of) | UMI | 3,455 | 1,590 | 7,506 | Non-severe rate x 72% (WHO) (44) |
| Viet Nam | LMI | 2,470 | 1,816 | 3,354 | Non-severe rate x 66% (WHO) (44) |
| Yemen | LMI | 2,470 | 1,816 | 3,354 | Non-severe rate x 66% (WHO) (44) |
| Zambia | LMI | 2,470 | 1,816 | 3,354 | Non-severe rate x 66% (WHO) (44) |
| Zimbabwe | LI | 2,385 | 1,422 | 4,005 | Non-severe rate x 54% (WHO) (44) |

Acronym: LI, low income; LMI, low-middle income; RSV, respiratory syncytial virus; UMI, upper middle income.

1. *Age distribution for severe RSV disease, aged <5 years.*

Current estimates for RSV disease age distributions are reported in wide age bands (1, 28). More granular age distributions (260 weeks of age <5 years) allow more accurate estimation of the potential impact of RSV prevention strategies. We identified 6 country datasets of RSV hospital admissions with ≥3 age groups in the first year of life and a collective sample size of ≥30 children. Four studies (Kenya (45), Mozambique (46), Pakistan (47), and South Africa (48)) were identified from the reference list of Li, 2022 (1). We also included unpublished data from two other countries (Argentina and Vietnam).

We fitted a range of parametric distributions to the age distributions in each country and chose the distribution with the best goodness-of-fit (the 3-parameter Burr distribution). A pooled Burr distribution was then fitted to the average of the 6 frequency estimates in each week of age (Figure S1).

**Figure S1.** Age distribution of severe RSV-ALRI cases in first year of life.


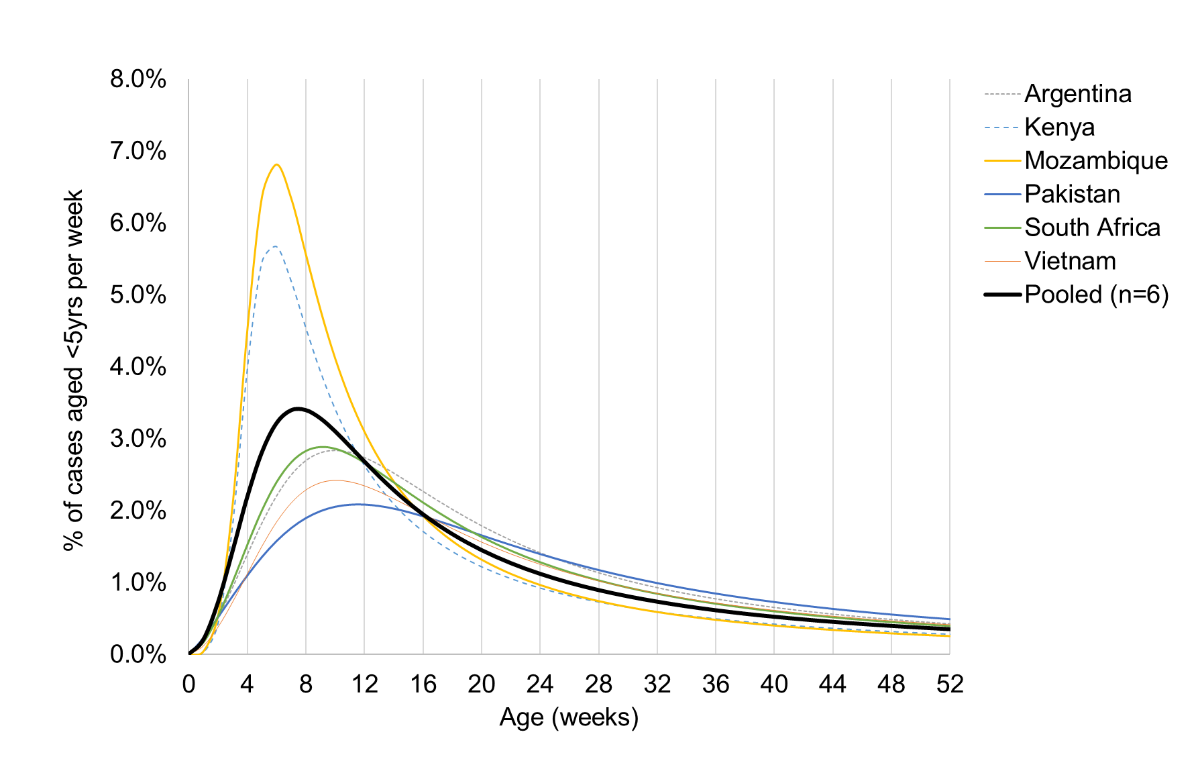


1. *Age distribution for non-severe RSV disease, aged <5 years.*

The ratio of severe to non-severe RSV disease was reported in broad age bands (<3m, 3-5m, 6-11m, 12-59m) in Li, 2022 (1). These ratios were applied to the fitted age distribution for severe RSV disease, and the Burr distribution was refit to generate the age distribution for non-severe RSV disease by week of age (Figure S2).

**Figure S2.** Age distribution of non-severe and severe RSV-ALRI cases by age in first year of

life.


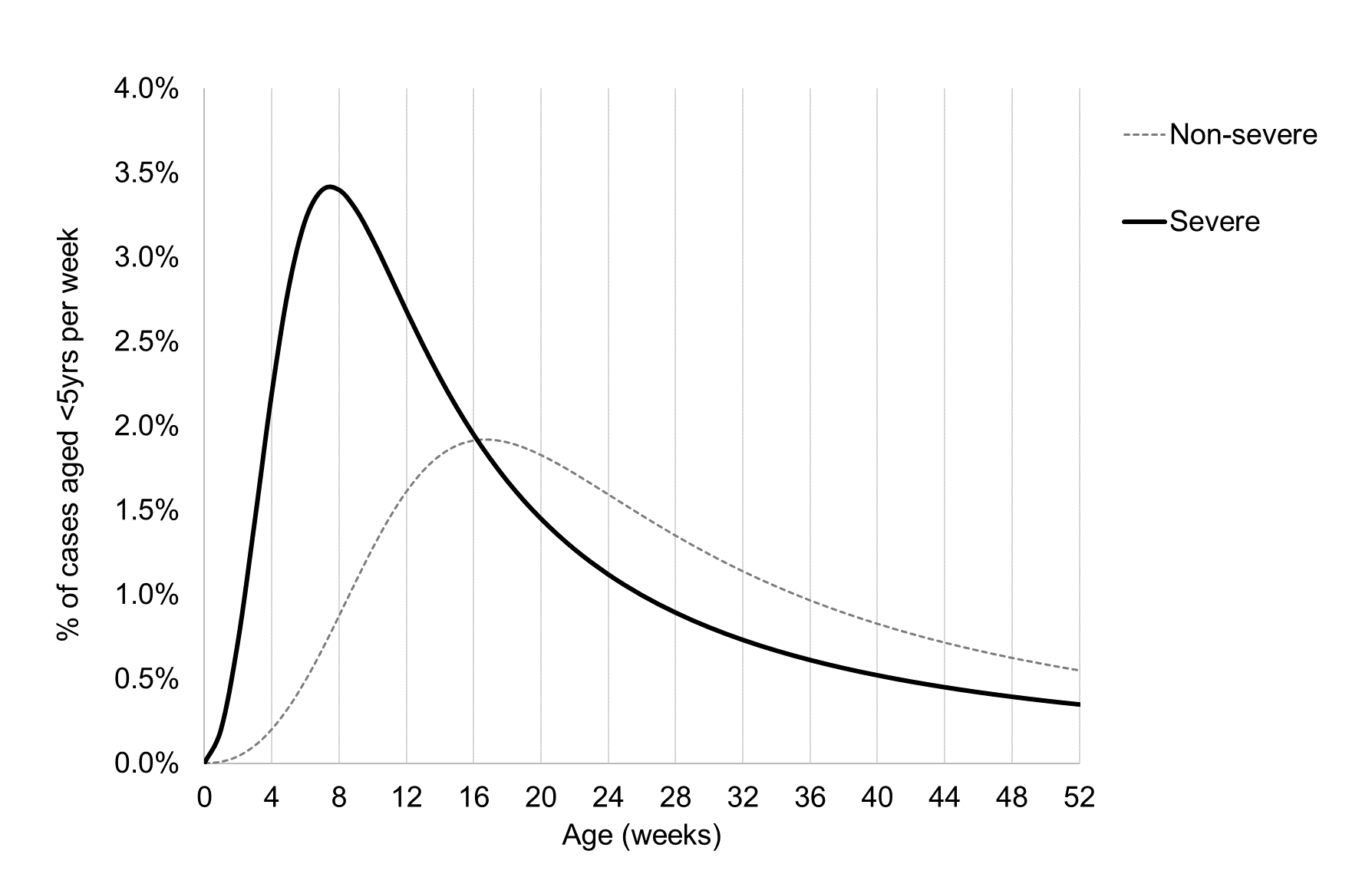


1. *DALY weights and duration of illness.*

The DALY weights were extracted from GBD, 2019 (49) using the DALY weights for acute episode of moderate and severe lower respiratory infections at 0.051 (95% CI: 0.032-0.074) and 0.133 (95% CI: 0.088-0.190), respectively, as proxies for non-severe and severe RSV infections. The DALY weight for partially controlled asthma at 0.036 (95% CI: 0.022-0.055) was used as a proxy for recurrent wheezing (49).

The average duration of RSV infection was assumed to be 5 and 10 days for non-severe RSV and severe RSV, respectively (60). Asthma was excluded from this analysis, but if included, would have had an assumed duration of around 3 years.

1. *Cost per RSV clinic visit and cost per RSV hospital admission*

Costs were extracted from Zhang *et al* (51), a global systematic review of the cost of managing severe pneumonia. This review identified 27 studies from 14 LMICs (Argentina, Bangladesh, Brazil, Colombia, Fiji, Guinea, India, Indonesia, Jordan, Kenya, Pakistan, South Africa, Vietnam, Zambia). For the cost per RSV clinic visit, we used the mean cost in LMICs of managing severe pneumonia in an outpatient setting. For the cost per RSV hospital admission separate mean costs were provided for managing severe pneumonia in primary/secondary and tertiary hospital care in LMICs, so we calculated a weighted average assuming 20% of hospital care was administered in tertiary hospitals. An inflation calculator (57) was applied to inflate 2013 USD to 2022 USD (January).

The costs reported in Zhang *et al* (51) represent all-cause pneumonia rather than RSV-attributable pneumonia and/or bronchiolitis. There was also substantial variation in the cost perspective and age ranges reported by the different studies. RSV healthcare costs should therefore be strengthened at country level to ensure they capture all items relevant to the chosen cost perspective, and to ensure they represent children aged <5 years.

1. *Coverage of RSV prevention strategies*

Maternal vaccination coverage was based on ANC coverage reported in Baral, 2020 (16). If a country did not have an estimate, a proxy country was selected, based on neighbouring countries of similar income level.

Infant mAb coverage was based on national immunization coverage estimates for BCG in the year 2021. These were extracted from WHO/UNICEF (50). Estimates for Kosovo were unavailable. Instead, coverage from Bosnia was used as a proxy, a neighbouring country with similar income level.

**Table S9.** Estimates of maternal coverage (using ANC) as a proxy for maternal RSV vaccine and national immunization coverage of existing vaccines as a proxy for mAb.

| **Country** | **World Bank Income** | **WHO region** | **Maternal coverage (%)** | **Source** | **BCG coverage (%)** | **Source** |
| --- | --- | --- | --- | --- | --- | --- |
| Afghanistan | LI | EMR | 16 | Baral, 2020 (16) | 87 | WUENIC, 2021 (50) |
| Albania | UMI | EUR | 71 | Baral, 2020 (16) | 98 | WUENIC, 2021 (50) |
| Algeria | UMI | AFR | 75 | Baral, 2020 (16) | 99 | WUENIC, 2021 (50) |
| Angola | LMI | AFR | 51 | Baral, 2020 (16) | 58 | WUENIC, 2021 (50) |
| Argentina | UMI | AMR | 84 | Baral, 2020 (16) | 85 | WUENIC, 2021 (50) |
| Armenia | LMI | EUR | 94 | Baral, 2020 (16) | 99 | WUENIC, 2021 (50) |
| Azerbaijan | UMI | EUR | 53 | Baral, 2020 (16) | 94 | WUENIC, 2021 (50) |
| Bangladesh | LMI | SEAR | 25 | Baral, 2020 (16) | 99 | WUENIC, 2021 (50) |
| Belarus | UMI | EUR | 94 | Baral, 2020 (16) | 97 | WUENIC, 2021 (50) |
| Belize | UMI | AMR | 89 | Baral, 2020 (16) | 76 | WUENIC, 2021 (50) |
| Benin | LI | AFR | 56 | Baral, 2020 (16) | 88 | WUENIC, 2021 (50) |
| Bhutan | LMI | SEAR | 81 | Baral, 2020 (16) | 98 | WUENIC, 2021 (50) |
| Bolivia (Plurinational State of) | LMI | AMR | 59 | Baral, 2020 (16) | 82 | WUENIC, 2021 (50) |
| Bosnia and Herzegovina | UMI | EUR | 69 | Baral, 2020 (16) | 95 | WUENIC, 2021 (50) |
| Botswana | UMI | AFR | 79 | Baral, 2020 (16) | 98 | WUENIC, 2021 (50) |
| Brazil | UMI | AMR | 84 | Baral, 2020 (16) | 67 | WUENIC, 2021 (50) |
| Bulgaria | UMI | EUR | 81 | Baral, 2020 (16) | 97 | WUENIC, 2021 (50) |
| Burkina Faso | LI | AFR | 58 | Baral, 2020 (16) | 98 | WUENIC, 2021 (50) |
| Burundi | LI | AFR | 39 | Baral, 2020 (16) | 83 | WUENIC, 2021 (50) |
| Cabo Verde | LMI | AFR | 81 | Baral, 2020 (16) | 98 | WUENIC, 2021 (50) |
| Cambodia | LMI | WPR | 62 | Baral, 2020 (16) | 98 | WUENIC, 2021 (50) |
| Cameroon | LMI | AFR | 55 | Baral, 2020 (16) | 80 | WUENIC, 2021 (50) |
| Central African Republic | LI | AFR | 30 | Baral, 2020 (16) | 61 | WUENIC, 2021 (50) |
| Chad | LI | AFR | 19 | Baral, 2020 (16) | 60 | WUENIC, 2021 (50) |
| China | UMI | WPR | 79 | Baral, 2020 (16) | 99 | WUENIC, 2021 (50) |
| Colombia | UMI | AMR | 86 | Baral, 2020 (16) | 89 | WUENIC, 2021 (50) |
| Comoros | LI | AFR | 54 | Baral, 2020 (16) | 91 | WUENIC, 2021 (50) |
| Congo | LMI | AFR | 74 | Baral, 2020 (16) | 72 | WUENIC, 2021 (50) |
| Costa Rica | UMI | AMR | 89 | Baral, 2020 (16) | 99 | WUENIC, 2021 (50) |
| Côte d'Ivoire | LMI | AFR | 55 | Baral, 2020 (16) | 86 | WUENIC, 2021 (50) |
| Croatia | UMI | EUR | 69 | Baral, 2020 (16) | 98 | WUENIC, 2021 (50) |
| Cuba | UMI | AMR | 93 | Baral, 2020 (16) | 99 | WUENIC, 2021 (50) |
| Dem. People's Republic of Korea | LI | SEAR | 87 | Baral, 2020 (16) | 99 | WUENIC, 2021 (50) |
| Democratic Republic of the Congo | LI | AFR | 46 | Baral, 2020 (16) | 73 | WUENIC, 2021 (50) |
| Djibouti | LMI | EMR | 44 | Baral, 2020 (16) | 77 | WUENIC, 2021 (50) |
| Dominican Republic | UMI | AMR | 89 | Baral, 2020 (16) | 85 | WUENIC, 2021 (50) |
| Ecuador | UMI | AMR | 59 | Baral, 2020 (16) | 81 | WUENIC, 2021 (50) |
| Egypt | LMI | EMR | 68 | Baral, 2020 (16) | 96 | WUENIC, 2021 (50) |
| El Salvador | LMI | AMR | 85 | Baral, 2020 (16) | 79 | WUENIC, 2021 (50) |
| Equatorial Guinea | UMI | AFR | 73 | Baral, 2020 (16) | 85 | WUENIC, 2021 (50) |
| Eritrea | LI | AFR | 58 | Baral, 2020 (16) | 97 | WUENIC, 2021 (50) |
| Eswatini | LMI | AFR | 73 | Baral, 2020 (16) | 97 | WUENIC, 2021 (50) |
| Ethiopia | LI | AFR | 24 | Baral, 2020 (16) | 70 | WUENIC, 2021 (50) |
| Fiji | UMI | WPR | 94 | Baral, 2020 (16) | 99 | WUENIC, 2021 (50) |
| Gabon | UMI | AFR | 76 | Baral, 2020 (16) | 85 | WUENIC, 2021 (50) |
| Gambia | LI | AFR | 65 | Baral, 2020 (16) | 88 | WUENIC, 2021 (50) |
| Georgia | LMI | EUR | 82 | Baral, 2020 (16) | 96 | WUENIC, 2021 (50) |
| Ghana | LMI | AFR | 75 | Baral, 2020 (16) | 93 | WUENIC, 2021 (50) |
| Grenada | UMI | AMR | 92 | Baral, 2020 (16) | 92 | WUENIC, 2021 (50) |
| Guatemala | LMI | AMR | 71 | Baral, 2020 (16) | 86 | WUENIC, 2021 (50) |
| Guinea | LI | AFR | 42 | Baral, 2020 (16) | 73 | WUENIC, 2021 (50) |
| Guinea-Bissau | LI | AFR | 65 | Baral, 2020 (16) | 67 | WUENIC, 2021 (50) |
| Guyana | UMI | AMR | 72 | Baral, 2020 (16) | 94 | WUENIC, 2021 (50) |
| Haiti | LI | AMR | 67 | Baral, 2020 (16) | 73 | WUENIC, 2021 (50) |
| Honduras | LMI | AMR | 83 | Baral, 2020 (16) | 83 | WUENIC, 2021 (50) |
| India | LMI | SEAR | 41 | Baral, 2020 (16) | 85 | WUENIC, 2021 (50) |
| Indonesia | LMI | SEAR | 70 | Baral, 2020 (16) | 87 | WUENIC, 2021 (50) |
| Iran (Islamic Republic of) | UMI | EMR | 91 | Baral, 2020 (16) | 98 | WUENIC, 2021 (50) |
| Iraq | UMI | EMR | 51 | Baral, 2020 (16) | 99 | WUENIC, 2021 (50) |
| Jamaica | UMI | AMR | 87 | Baral, 2020 (16) | 99 | WUENIC, 2021 (50) |
| Jordan | LMI | EMR | 84 | Baral, 2020 (16) | 76 | WUENIC, 2021 (50) |
| Kazakhstan | UMI | EUR | 86 | Baral, 2020 (16) | 93 | WUENIC, 2021 (50) |
| Kenya | LMI | AFR | 62 | Baral, 2020 (16) | 92 | WUENIC, 2021 (50) |
| Kiribati | LMI | WPR | 64 | Baral, 2020 (16) | 93 | WUENIC, 2021 (50) |
| Kosovo | LMI | EUR | 69 | Baral, 2020 (16) | 95 | WUENIC, 2021 (50) |
| Kyrgyzstan | LMI | EUR | 88 | Baral, 2020 (16) | 96 | WUENIC, 2021 (50) |
| Lao People's Democratic Republic | LMI | WPR | 22 | Baral, 2020 (16) | 90 | WUENIC, 2021 (50) |
| Lebanon | UMI | EMR | 83 | Baral, 2020 (16) | 77 | WUENIC, 2021 (50) |
| Lesotho | LMI | AFR | 70 | Baral, 2020 (16) | 87 | WUENIC, 2021 (50) |
| Liberia | LI | AFR | 77 | Baral, 2020 (16) | 85 | WUENIC, 2021 (50) |
| Libya | UMI | EMR | 79 | Baral, 2020 (16) | 74 | WUENIC, 2021 (50) |
| Madagascar | LI | AFR | 36 | Baral, 2020 (16) | 73 | WUENIC, 2021 (50) |
| Malawi | LI | AFR | 57 | Baral, 2020 (16) | 87 | WUENIC, 2021 (50) |
| Malaysia | UMI | WPR | 82 | Baral, 2020 (16) | 99 | WUENIC, 2021 (50) |
| Maldives | UMI | SEAR | 84 | Baral, 2020 (16) | 99 | WUENIC, 2021 (50) |
| Mali | LI | AFR | 32 | Baral, 2020 (16) | 78 | WUENIC, 2021 (50) |
| Marshall Islands | UMI | WPR | 64 | Baral, 2020 (16) | 89 | WUENIC, 2021 (50) |
| Mauritania | LMI | AFR | 63 | Baral, 2020 (16) | 80 | WUENIC, 2021 (50) |
| Mauritius | UMI | AFR | 79 | Baral, 2020 (16) | 96 | WUENIC, 2021 (50) |
| Mexico | UMI | AMR | 92 | Baral, 2020 (16) | 28 | WUENIC, 2021 (50) |
| Micronesia (Fed. States of) | LMI | WPR | 53 | Baral, 2020 (16) | 84 | WUENIC, 2021 (50) |
| Mongolia | LMI | WPR | 86 | Baral, 2020 (16) | 99 | WUENIC, 2021 (50) |
| Montenegro | UMI | EUR | 77 | Baral, 2020 (16) | 67 | WUENIC, 2021 (50) |
| Morocco | LMI | EMR | 35 | Baral, 2020 (16) | 99 | WUENIC, 2021 (50) |
| Mozambique | LI | AFR | 42 | Baral, 2020 (16) | 91 | WUENIC, 2021 (50) |
| Myanmar | LMI | SEAR | 47 | Baral, 2020 (16) | 87 | WUENIC, 2021 (50) |
| Namibia | UMI | AFR | 72 | Baral, 2020 (16) | 94 | WUENIC, 2021 (50) |
| Nepal | LI | SEAR | 56 | Baral, 2020 (16) | 92 | WUENIC, 2021 (50) |
| Nicaragua | LMI | AMR | 77 | Baral, 2020 (16) | 93 | WUENIC, 2021 (50) |
| Niger | LI | AFR | 36 | Baral, 2020 (16) | 94 | WUENIC, 2021 (50) |
| Nigeria | LMI | AFR | 34 | Baral, 2020 (16) | 67 | WUENIC, 2021 (50) |
| North Macedonia | UMI | EUR | 90 | Baral, 2020 (16) | 93 | WUENIC, 2021 (50) |
| Pakistan | LMI | EMR | 33 | Baral, 2020 (16) | 91 | WUENIC, 2021 (50) |
| State of Palestine | LMI | EMR | 84 | Baral, 2020 (16) | 99 | WUENIC, 2021 (50) |
| Panama | UMI | AMR | 86 | Baral, 2020 (16) | 99 | WUENIC, 2021 (50) |
| Papua New Guinea | LMI | WPR | 48 | Baral, 2020 (16) | 52 | WUENIC, 2021 (50) |
| Paraguay | UMI | AMR | 74 | Baral, 2020 (16) | 84 | WUENIC, 2021 (50) |
| Peru | UMI | AMR | 88 | Baral, 2020 (16) | 87 | WUENIC, 2021 (50) |
| Philippines | LMI | WPR | 77 | Baral, 2020 (16) | 64 | WUENIC, 2021 (50) |
| Republic of Moldova | LMI | EUR | 91 | Baral, 2020 (16) | 95 | WUENIC, 2021 (50) |
| Romania | UMI | EUR | 53 | Baral, 2020 (16) | 97 | WUENIC, 2021 (50) |
| Russian Federation | UMI | EUR | 81 | Baral, 2020 (16) | 98 | WUENIC, 2021 (50) |
| Rwanda | LI | AFR | 67 | Baral, 2020 (16) | 89 | WUENIC, 2021 (50) |
| Saint Lucia | UMI | AMR | 88 | Baral, 2020 (16) | 89 | WUENIC, 2021 (50) |
| Saint Vincent and the Grenadines | UMI | AMR | 95 | Baral, 2020 (16) | 99 | WUENIC, 2021 (50) |
| Samoa | UMI | WPR | 76 | Baral, 2020 (16) | 99 | WUENIC, 2021 (50) |
| Sao Tome and Principe | LMI | AFR | 80 | Baral, 2020 (16) | 95 | WUENIC, 2021 (50) |
| Senegal | LI | AFR | 66 | Baral, 2020 (16) | 95 | WUENIC, 2021 (50) |
| Serbia | UMI | EUR | 89 | Baral, 2020 (16) | 98 | WUENIC, 2021 (50) |
| Sierra Leone | LI | AFR | 76 | Baral, 2020 (16) | 72 | WUENIC, 2021 (50) |
| Solomon Islands | LMI | WPR | 64 | Baral, 2020 (16) | 82 | WUENIC, 2021 (50) |
| Somalia | LI | EMR | 4 | Baral, 2020 (16) | 37 | WUENIC, 2021 (50) |
| South Africa | UMI | AFR | 75 | Baral, 2020 (16) | 86 | WUENIC, 2021 (50) |
| South Sudan | LI | AFR | 23 | Baral, 2020 (16) | 52 | WUENIC, 2021 (50) |
| Sri Lanka | LMI | SEAR | 81 | Baral, 2020 (16) | 99 | WUENIC, 2021 (50) |
| Sudan | LMI | EMR | 50 | Baral, 2020 (16) | 92 | WUENIC, 2021 (50) |
| Suriname | UMI | AMR | 70 | Baral, 2020 (16) | 79 | WUENIC, 2021 (50) |
| Syrian Arab Republic | LMI | EMR | 55 | Baral, 2020 (16) | 74 | WUENIC, 2021 (50) |
| Tajikistan | LMI | EUR | 48 | Baral, 2020 (16) | 98 | WUENIC, 2021 (50) |
| Thailand | UMI | SEAR | 90 | Baral, 2020 (16) | 99 | WUENIC, 2021 (50) |
| Timor-Leste | LMI | SEAR | 46 | Baral, 2020 (16) | 88 | WUENIC, 2021 (50) |
| Togo | LI | AFR | 44 | Baral, 2020 (16) | 96 | WUENIC, 2021 (50) |
| Tonga | UMI | WPR | 83 | Baral, 2020 (16) | 99 | WUENIC, 2021 (50) |
| Tunisia | LMI | EMR | 78 | Baral, 2020 (16) | 85 | WUENIC, 2021 (50) |
| Turkey | UMI | EUR | 81 | Baral, 2020 (16) | 96 | WUENIC, 2021 (50) |
| Turkmenistan | UMI | EUR | 92 | Baral, 2020 (16) | 98 | WUENIC, 2021 (50) |
| Tuvalu | UMI | WPR | 64 | Baral, 2020 (16) | 99 | WUENIC, 2021 (50) |
| Uganda | LI | AFR | 63 | Baral, 2020 (16) | 91 | WUENIC, 2021 (50) |
| Ukraine | LMI | EUR | 65 | Baral, 2020 (16) | 93 | WUENIC, 2021 (50) |
| United Republic of Tanzania | LI | AFR | 54 | Baral, 2020 (16) | 87 | WUENIC, 2021 (50) |
| Uzbekistan | LMI | EUR | 78 | Baral, 2020 (16) | 99 | WUENIC, 2021 (50) |
| Vanuatu | LMI | WPR | 44 | Baral, 2020 (16) | 77 | WUENIC, 2021 (50) |
| Venezuela (Bolivarian Republic of) | UMI | AMR | 86 | Baral, 2020 (16) | 82 | WUENIC, 2021 (50) |
| Viet Nam | LMI | WPR | 70 | Baral, 2020 (16) | 95 | WUENIC, 2021 (50) |
| Yemen | LMI | EMR | 22 | Baral, 2020 (16) | 71 | WUENIC, 2021 (50) |
| Zambia | LMI | AFR | 64 | Baral, 2020 (16) | 85 | WUENIC, 2021 (50) |
| Zimbabwe | LI | AFR | 70 | Baral, 2020 (16) | 88 | WUENIC, 2021 (50) |

Abbreviations: AFR, Africa region; AMR, Americas region; CAR, Central African Republic; Dem., Democratic; DHS, Demographic and Health Surveys; EMR, Eastern Mediterranean Region; EUR, Europe region; LI, low income; LMI, low middle income; m, months; MICS, Multiple Indicator Cluster Surveys; Rep., republic; SEAR, South East Asia region; w, weeks; WHO, World Health Organization; WPR, Western Pacific region; UMI, upper middle income

1. *Efficacy of RSV prevention strategies*

A press release by Pfizer on 1^st^ November 2022 (34) summarised the most recent efficacy data for its bivalent prefusion F-based maternal vaccine (RSVpreF). A Phase 3 clinical trial reported efficacy against severe medically attended LRTI of 81.8% (95% CI 40.6 - 96.3%) 90 days (3 months) after birth and 69.4% (95% CI 44.3 - 84.1) 6 months after birth. Efficacy against any medically attended RSV LRTI was reported to be 57.1% (95% CI 14.7-79.8%) 3 months after birth and 51.3% (95% CI 29.4-66.8%) 6 months after birth.

A press release by Sanofi on 4^th^ November 2022 (52) outlined the most recent efficacy data for Beyfortus® (nirsevimab). A pooled analysis of healthy preterm and term infants reported efficacy of 77.3% (95% CI 50.4 – 89.7%) against RSV LRTI hospitalisations after 151 days (5 months). The pooled analysis combined evidence from the Phase 3 MELODY trial (21 countries, 1490 infants, July 2019 - March 2020) and the Phase 2b trial (23 countries, 1453 infants, Nov 2016 - Dec 2017). The primary endpoint from the Phase 3 MELODY trial was reported to be 74.5% (95% CI 49.6 – 87.1%) against any medically attended RSV LRTI.

Figure S3 shows the efficacy scenarios used for both interventions.

**Figure S3. Efficacy scenarios used for maternal vaccination and monoclonal antibody**


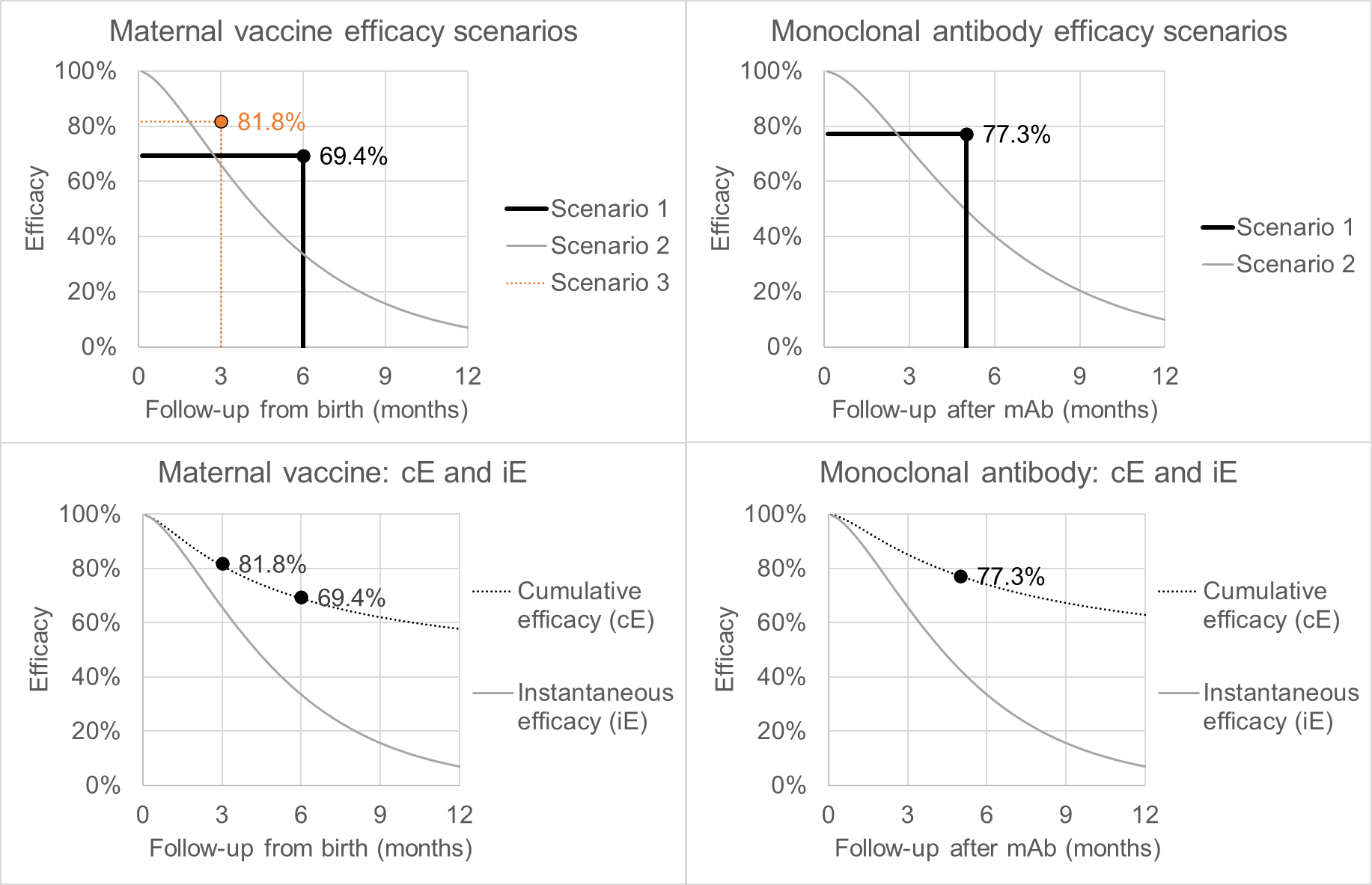


Caption: The thick black lines in the top row show the base case (scenario 1) assumptions. For maternal vaccination this assumes efficacy of 69.4% (severe RSV disease) and 51.3% (non-severe RSV disease) for a 6-month period and zero protection thereafter. For mAb this assumes efficacy of 77.3% (severe RSV disease) and 74.5% (non-severe RSV disease) for a 5-month period, and zero protection thereafter. The grey lines in the top row show the scenario 2 assumptions. For this scenario we used previously described methods (53) to fit estimates of instantaneous efficacy (iE) that were consistent with the reported cumulative efficacy (cE) at 3 and 6 months of follow-up (see plots on the bottom row of the figure). As part of the fitting process we assumed the pooled age distribution of RSV disease described earlier (page 27) and assumed that iE could not become negative. Finally we applied one additional scenario (scenario 3) for maternal vaccination with efficacy of 81.8% (severe RSV disease) and 57.1% (non-severe RSV disease) for a 3-month period, and zero protection thereafter.

1. *Vaccine programme costs*

The price per dose was assumed to be $3.50 in Gavi countries for both RSV mAb and RSV maternal vaccination. In non-Gavi countries the price was assumed to be $7.00. Syringe and safety box prices were derived from the UNICEF supply catalogue, with the assumption a 2ml syringe will be adequate to administer doses (54).

For vaccine wastage, we assumed a rate of 5% (% of price per dose) for doses, syringes and safety boxes based on the maximum acceptable vaccine wastage according to WHO (55).

International handling fees were derived from the UNICEF Handling Fees (as % of price per dose) (58) with 1.40% assumed for Gavi countries and 3.5% for non-Gavi countries.

International transportation fees tend to vary depending on distance from manufacturer to country of interest, however, a mid-value of 6% (% of price per dose) was used based on a similar assumption for a previous study on rotavirus vaccines (56).

The incremental health system delivery cost per dose was assumed to be $0.74 in low income countries and $2.02 in middle-income countries, based on a study by Baral et al (16).

All costs were inflated (57) to 2022 USD (January).

**Methods used for economic evaluation of maternal vaccination and infant mAb in 133 LMICs**

In the main paper, we present an economic evaluation of maternal vaccination and infant mAb for the purpose of a model comparison, and also to identify influential input parameters. The methods of this evaluation are summarised below, using the standard WHO checklist for appraisal of economic evaluations of immunisation programmes (18).

**Table S10.** WHO checklist for appraisal of economic evaluation and model comparison exercise

| **Aspect** | **Questions for critical appraisal** | **Relevant text in main paper** |
| --- | --- | --- |
| Framing the analysis | Is there a clear statement of the study question? | “The paper…compares the cost-effectiveness results of UNIVAC to a separate model published by PATH [16], and identifies the inputs with largest influence on the cost-effectiveness results.  (Introduction).  “Each model evaluated the cost-effectiveness of two interventions (maternal vaccine, infant mAb) over the lifetimes of a single birth cohort (2025) in 133 LMICs.”  (Methods) |
|  | Have the comparators being compared been clearly described? | “Each RSV prevention strategy was compared to nothing (a scenario without any pharmaceutical RSV intervention) and to each other.” (Methods). |
|  | Has a cost-utility analysis been performed? If not, has that decision been justified appropriately? | “A standardised output spreadsheet was used to compare modelled estimates of the cost per DALY averted, and other outcome measures, across 133 LMICs.”  (Methods). |
|  | Is the perspective of the analysis clearly stated? If a societal or multiple perspectives have been adopted, have the costs and outcomes been disaggregated to allow judgements to be made from different perspectives? Are the costs and outcomes reported consistent with the perspective reported? | “We used a discount rate of 3% for future costs and health effects, a currency year of US$ 2022 (January) and a societal cost perspective.”  (Methods)  “The costs reported in Zhang et al (51) represent all-cause pneumonia rather than RSV-attributable pneumonia and/or bronchiolitis. There was also substantial variation in the cost perspective and age ranges reported by the different studies. RSV healthcare costs should therefore be strengthened at country level to ensure they capture all items relevant to the chosen cost perspective, and to ensure they represent children aged <5 years.”  (Additional File 1, page 29) |
|  | Is/are the institution(s) sponsoring the study and the individual authors clearly stated? | “This work was supported, in full, by the Bill & Melinda Gates Foundation [Grant Number].” (Funding).  “Sarwat Mahmud, MPH, Ranju Baral, PhD, Colin Sanderson, PhD, Clint Pecenka PhD, Mark Jit PhD, Andy Clark PhD  Department of Health Services Research and Policy, Faculty of Public Health and Policy, London School of Hygiene and Tropical Medicine, London, UK.  PATH, Seattle, WA, USA.  Department of Infectious Disease Epidemiology, London School of Hygiene and Tropical Medicine, London, UK; Modelling and Economics Unit, Public Health England, London, UK.’  (Title). |
|  | Are the time frame and analytic horizon clearly stated and justified? | “Each model evaluated the cost-effectiveness of two interventions (maternal vaccine, infant mAb) over the lifetimes of a single birth cohort (2025) in 133 LMICs.” (Methods) |
|  | Are broader economic benefits besides improved health, reduced health care expenditure and short-term productivity gains incorporated? If yes, is this consistent with the way vaccines are funded and the decision-maker(s)' objectives? | Broader economic benefits are not included. |
| Costs | Have the methods used for the estimation of costs been clearly stated? | “UNIVAC calculates total healthcare costs by multiplying the number of clinic visits and hospital admissions by the respective average cost per visit or hospital admission. Average costs of visits / admissions per disease episode can be calculated outside the model by calculating the cost per visit or admission for different types of healthcare provider and the share of total visits/admissions provided by each. It is assumed that the mix of providers does not change after vaccination.”  (Methods, page 8).  (Additional File 1, page 29)  “The cost of RSV prevention strategies includes a number of parameters, including the price and wastage of doses, syringes and safety boxes. International handling fees and international transportation fees are also applied, together with the incremental cost to the health system of introducing the new RSV prevention strategy. Full calculations are provided in the supplementary appendix (page 5).”  (Methods) (Additional File 1, page 5)  (Additional File 1, page 33) |
|  | Has a summary of the expected resource use and unit costs for each alternative been provided, including a specification of the assumptions behind calculations of the costs? | (Additional File 1, page 29)  (Additional File 1, page 33) |
|  | Have the data sources used to estimate costs been clearly stated? | “Table 2. Programme impact and cost assumptions used for RSV prevention strategies in the model comparison exercise.” (Methods, page 15)  (Additional File 1, page 29)  (Additional File 1, page 33) |
|  | If productivity losses were estimated have they been reported separately? Has their relevance been discussed? Have the methods used to estimate productivity losses been described and justified? | “The costs reported in Zhang et al (51) represent all-cause pneumonia rather than RSV-attributable pneumonia and/or bronchiolitis. There was also substantial variation in the cost perspective and age ranges reported by the different studies. RSV healthcare costs should therefore be strengthened at country level to ensure they capture all items relevant to the chosen cost perspective, and to ensure they represent children aged <5 years.”  (Additional File 1, page 29) |
|  | Have future costs been included? If yes, was this a requirement of the reference case for the local policy maker, for whom the analysis was meant and have the results been presented with and without including these future costs? | “Each model evaluated the cost-effectiveness of two interventions (maternal vaccine, infant mAb) over the lifetimes of a single birth cohort (2025) in 133 LMICs.” (Methods, page 15) |
|  | Is the currency stated? If so, is the date of the currency and prices used in the model stated, with details of any adjustments or conversions provided? | “We used…a currency year of US$ 2022 (January).  (Methods, page 15)  “An inflation calculator (57) was applied to inflate 2013 USD to 2022 USD (January).”  (Additional File 1, page 29)  “All costs were inflated (57) to 2022 USD (January).”  (Additional File 1, page 33) |
| Effects | Was the evidence on vaccine efficacy identified systematically, and was taken account of the biological characteristics of the pathogen in question and how its infectious nature may have influenced the efficacy estimates derived from trials? | (Additional File 1, page 32)  “As a static model, [UNIVAC] takes no account of indirect effects, such as herd immunity. The importance of these effects is yet to be established for maternal RSV vaccines and infant mAbs.  (Methods) |
|  | Was the effective coverage of vaccines calculated by multiplying vaccination coverage adjusted for non-compliance, by vaccine efficacy adjusted for loss of potency due to heat and freeze exposure, where such data were available? | “Coverage of RSV prevention strategies…” (Additional File 1, page 29) |
|  | Was the population effectiveness (or "impact") of vaccines calculated using empirical information on both the direct and indirect effects of the vaccination program, and was this information integrated in a mathematical model? | “As a static model, [UNIVAC] takes no account of indirect effects, such as herd immunity. The importance of these effects is yet to be established for maternal RSV vaccines and infant mAbs.  (Methods) |
|  | Are adverse events from immunization impacts likely to have a substantial impact on the results of the analysis? If so, have they been included on both the costs and effects sides of the analysis? | “The model has the option to include serious adverse events, but recent clinical trials have reported similar rates of adverse rates in the vaccine and placebo groups, so this is not currently recommended.” (Methods) |
|  | Was the estimated duration of vaccine protection over time data driven, completely transparent, and subjected to thorough uncertainty analysis? | (Additional File 1, page 32) |
|  | Have estimates of burden been presented in natural units – cases, deaths, years of life lost (YLL)? Have estimates of DALYs or QALYs lost been presented as final outcome of burden of disease? | Table S12 and \|Table S13 (Additional File 1, page 45) |
|  | If suitable QALY weights were not readily available, have DALYs been used for cost-utility analysis? | “Table 1. Disease burden and healthcare costs used for model comparison exercise” (Methods) |
|  | When DALYs have been used in cost-utility analysis, were they subjected to social weighting, such as age weighting? If yes, was this explicitly desired by the policy maker the analysis was meant to advise? | Age weighting was not considered. |
| Modelling | Are the model structure and implicit or explicit assumptions clearly described? | UNIVAC decision-support model.  (Methods) |
|  | Is the model type (static, dynamic or stochastic) clearly stated and justified in light of likely changes to the force of infection and the role of chance in the transmission process? Have the model’s strengths and weaknesses been discussed? | “As a static model, [UNIVAC] takes no account of indirect effects, such as herd immunity. The importance of these effects is yet to be established for maternal RSV vaccines and infant mAbs.  (Methods)  “Unlike dynamic models, static models are unable to capture indirect effects, such as herd immunity. For this reason, static cohort models are likely to underestimate the benefit and cost-effectiveness of RSV prevention strategies. This limitation should be carefully communicated to decision-makers when sharing results. However it is currently unclear whether or to what extent RSV interventions will reduce infectiousness or prevent acquisition of RSV infection, and thus whether herd immunity is an issue here. Dynamic models also require data that is often unreported or highly uncertain in LMICs.” (Discussion) |
|  | Has the model been validated? If so, has it been validated in as many facets of validation as possible? | “We compared results from UNIVAC (developed by researchers at LSHTM) to those from a separate proportionate outcomes static cohort model developed in Stata by PATH (16), using established principles for multi-model comparisons (63).”  (Methods)  “UNIVAC results were consistent with the results of a separate model published by PATH.”  (Results) |
| Discounting | Is the discount rate clearly stated and justified? | “We used a discount rate of 3% for future costs and health effects.”  (Methods) |
|  | Have WHO recommended schemes of (i) 3% and 0% discounting for consumption and health respectively in the base case, and (ii) 3% discounting for both health and consumption been used? | Differential discounting was not considered. |
| Uncertainty | Are all known sources of uncertainties not accounted for justified to the extent possible? | “We ran scenario and sensitivity analyses to identify the parameters that had most influence on the UNIVAC cost-effectiveness results. For both RSV prevention strategies (maternal vaccine, infant mAb) we compared the percentage of 133 LMICs that would be willing to pay for each intervention, at different WTP thresholds, for the base case and eight alternative what-if scenarios (supplementary appendix, page 43). We also varied each parameter in turn by -/+10% i.e. by multiplying the central estimate by 0.9 and 1.1 respectively and noting the effect of this change on the cost per DALY averted.”  (Methods).  “There is a good deal of uncertainty around several influential inputs, including intervention price, efficacy, and duration of protection. Given these gaps in the current available evidence, our illustrative results should not be used to prioritise one intervention over the other. Our estimates should also not be considered a replacement for more thorough economic evaluations at country-level. Such studies should include engagement with stakeholders to build consensus on the most appropriate input data and scenarios for uncertainty analysis.”  (Discussion) |
|  | Have methodological uncertainties been accounted for with scenario analysis and model and parameter uncertainties with probabilistic sensitivity analysis? | “We ran scenario and sensitivity analyses to identify the parameters that had most influence on the UNIVAC cost-effectiveness results.” (Methods)  “UNIVAC also includes options to run probabilistic uncertainty analysis.” (Methods) |
|  | Has the full range of potential cost-effectiveness results been presented, preferably in a probabilistic way, but at a minimum by presenting a best and worst case scenario? | “We ran scenario and sensitivity analyses to identify the parameters that had most influence on the UNIVAC cost-effectiveness results.” (Methods)  “UNIVAC also includes options to run probabilistic uncertainty analysis.” (Methods) |
|  | Has the intervention identified as being the most cost-effective, the highest average net benefit? | “Infant mAb prevented more DALYs in 92% (122/133) of LMICs and had a lower net cost than maternal vaccination in 86% (115/133) of LMICs. Infant mAb was dominant (had both greater impact and lower net costs than maternal vaccination) in 79% (105/133) of LMICs..”  (Results). |
|  | Have the results been presented for a range of willingness-to-pay values? | “Figure 3. Percentage of 133 LMICs willing to pay for RSV prevention strategies at different willingness-to-pay thresholds.”  (Results) |
|  | Has the sensitivity of the results to the following variables been assessed: discount rate, vaccination effectiveness (where unknown or uncertain), incidence of disease (including complication rates where relevant), case fatality risks and vaccine price? | “We ran scenario and sensitivity analyses to identify the parameters that had most influence on the UNIVAC cost-effectiveness results.” (Methods) |
|  | Have the findings been compared to other economic evaluations undertaken in the same or neighbouring countries? | “We compared results from UNIVAC (developed by researchers at LSHTM) to those from a separate proportionate outcomes static cohort model developed in Stata by PATH (16), using established principles for multi-model comparisons (63).”  (Methods)  “UNIVAC results were consistent with the results of a separate model published by PATH.”  (Results) |
| Other factors | Is there a discussion of other important factors for the decision under consideration? | “WTP thresholds should not be the only criteria used for appraising RSV interventions at country-level (69).”  (Discussion) |
| Conclusions | Is an answer given to the study question? | “UNIVAC generated similar results to a separate model published by PATH.” (Discussion)  “Forthcoming RSV interventions (maternal vaccines and infant mAbs) are worth serious consideration in LMICs…”  (Abstract)  “Our cost-effectiveness results were most sensitive to changes in the price, efficacy and duration of protection of each strategy, and the rate (and cost) of RSV hospital admissions. Other parameters were also influential, such as the mean age of severe RSV disease.”  (Discussion) |
|  | Do the conclusions follow from the data reported? | “Forthcoming RSV interventions (maternal vaccines and infant mAbs) are worth serious consideration in LMICs…  (Abstract) |
|  | Are the conclusions accompanied by the appropriate caveats? | “…but there is a good deal of uncertainty around several influential inputs, including intervention price, efficacy, and duration of protection.”  (Abstract). |

**Scenarios evaluated to identify influential inputs/assumptions**

**Table S11.** Description of alternative scenarios

| Baseline |  | Description |
| --- | --- | --- |
| Scenario 1 | Base case | Maternal vaccination ($3.50 per dose, 69% efficacy, 6 months protection) and infant mAb ($3.50 per dose, 77% efficacy, 5 months protection). |
| Scenario 2 | Efficacy = Fitted gamma | Gradual waning of protection based on gamma curves fitted to available trial data. See page 32 for more details. |
| Scenario 3 | Efficacy = 3 months duration | This scenario is only applied to maternal vaccination and reflects of 81.8% (severe RSV disease) for a 3-month period, and zero protection thereafter. |
| Scenario 4 | Efficacy = Lower 95% CI | Assuming the lower bound of the 95% confidence interval for efficacy (maternal vaccine=44.3%, mAb=50.4%) |
| Scenario 5 | Efficacy = Upper 95% CI | Assuming the upper bound of the 95% confidence interval (maternal vaccine=84.1%, mAb=89.7%). |
| Scenario 6 | 10-year intervention period with declining RSV mortality | The analysis of 1 cohort (2025) was extended to 10 cohorts (2025-2034) with a declining annual trend in RSV mortality in the absence of intervention (consistent with overall trend in all-cause under-five deaths) to reflect improvements in living standards and access to care over time. |
| Scenario 7 | Price per dose halved | Half the price per dose (GAVI: $1.75, non-GAVI: $3.50). |
| Scenario 8 | Price per dose doubled | Double the price per dose (GAVI: $7.00, non-GAVI: $14.00). |

Acronyms: mAb, monoclonal antibody; RSV, respiratory syncytial virus; GAVI, Global Alliance for Vaccines and Immunizations.

**Scenario analysis**

**Figure S4.** Percentage of 133 LMICs willing to pay for mAb RSV intervention.


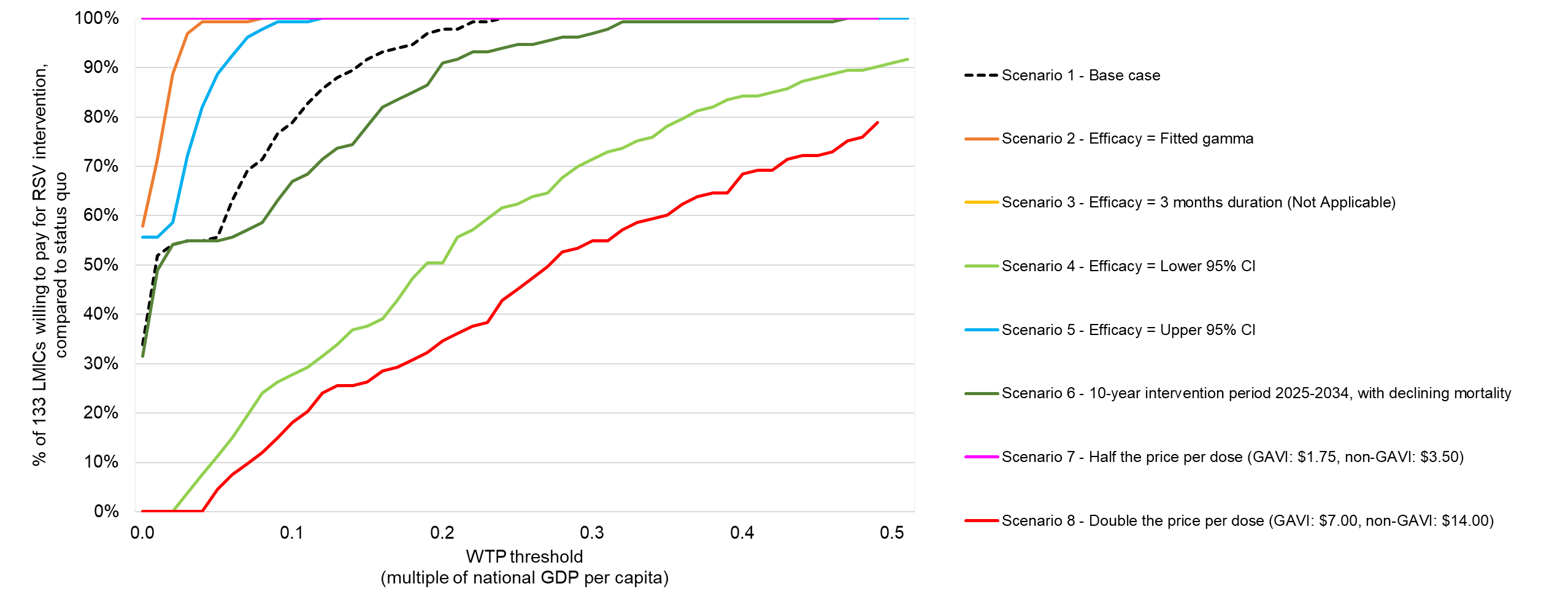


Caption: Several alternative ‘what-if’ scenarios were explored relative to the base case scenario, represented by the dotted line. For the base case scenario, almost all countries using mAb would be cost effective at 0.25 times the GDP per capita threshold. Scenarios below the base case scenario represent those less favourable, the least favourable being scenario 6, assuming the lower range of efficacy, and scenario 8, assuming double the price per dose. However, even with the least favourable assumptions, mAb would still be cost-effective in at least 80% of countries based on a WTP threshold of half the national GDP per capita.

**Figure S5.** Percentage of 133 LMICs willing to pay for maternal vaccine RSV intervention.

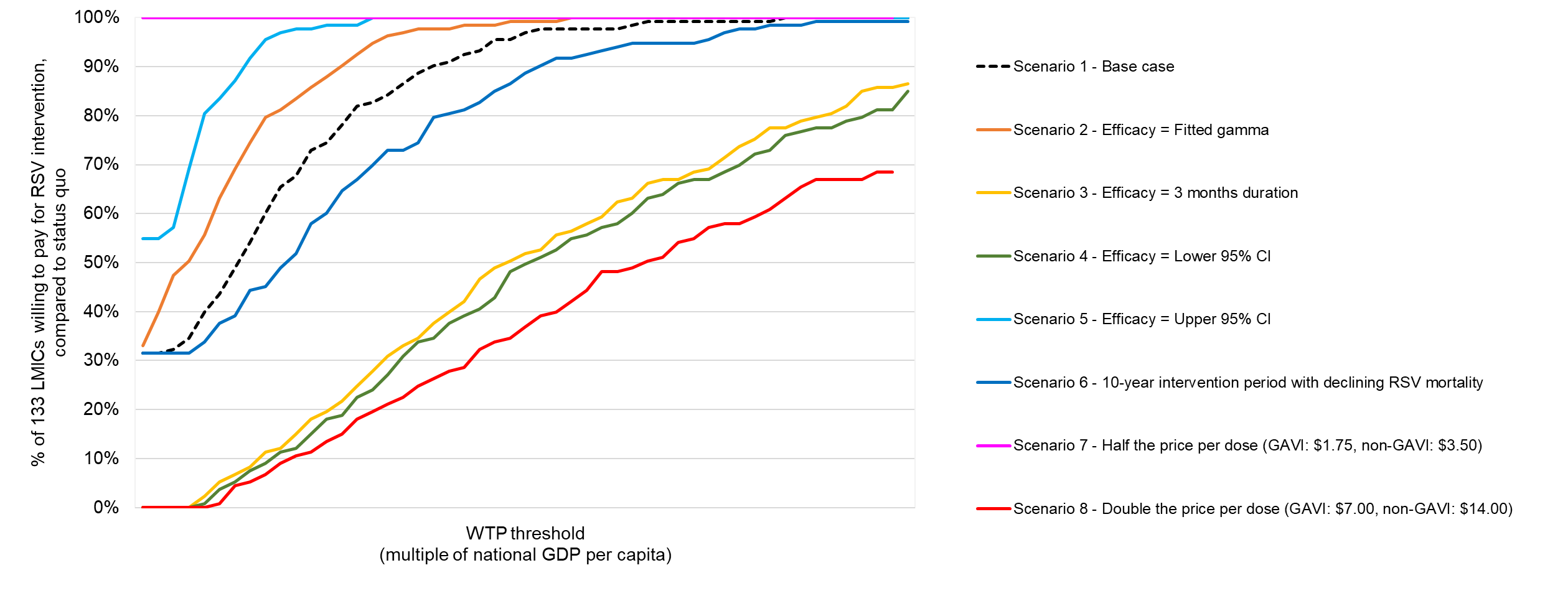


Caption: Several alternative ‘what-if’ scenarios were explored relative to the base case scenario, represented by the dotted line. For the base case scenario, almost all countries using maternal vaccine would be cost effective at <0.01 times the GDP per capita threshold. Scenarios below the base case scenario represent those less favourable, the least favourable being scenario 6, assuming the lower range of efficacy, and scenario 8, assuming double the price per dose. However, even with the least favourable assumptions, mAb would still be cost-effective in at least 90% of countries based on a WTP threshold of half the national GDP per capita.

**Sensitivity analysis**

**Figure S6.** Percentage difference in cost per DALY averted in a low-income country, relative to baseline cost of US$3 for mAb and US$36, when each parameter is varied by +/-10%


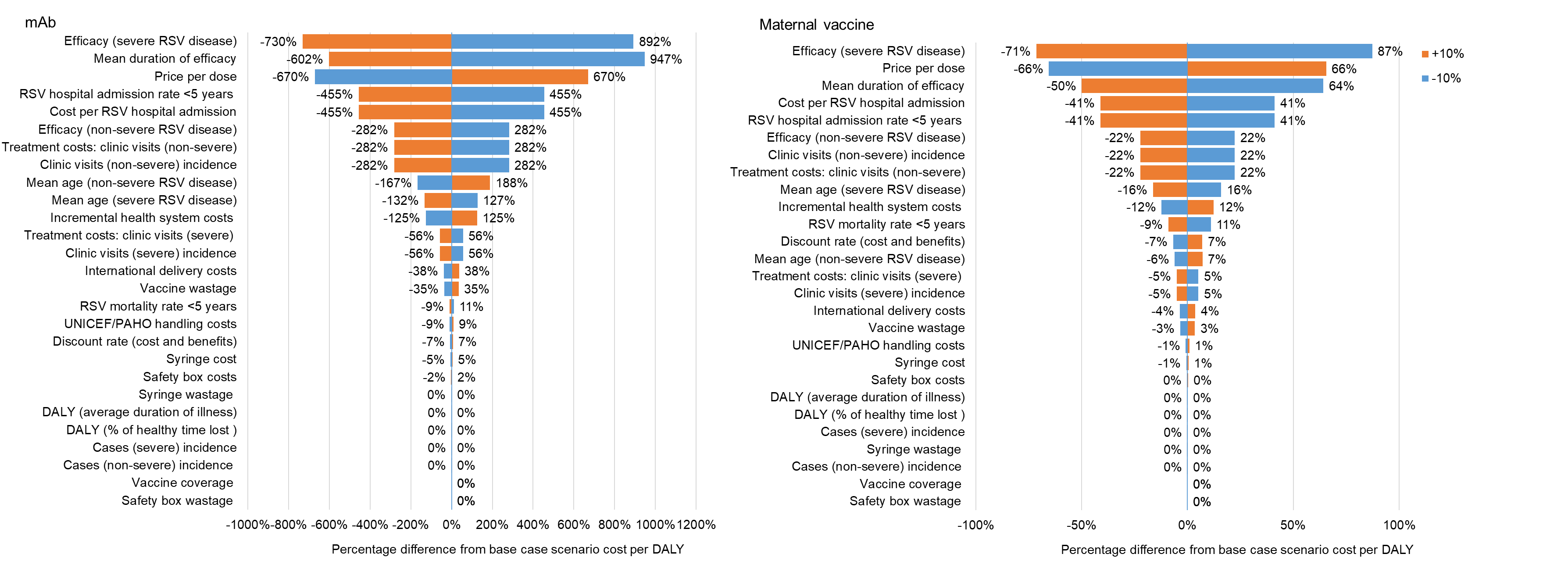


Caption: Cost per DALY averted was shown to be most sensitive to efficacy and duration of protection against severe RSV disease, price per dose, RSV hospital admission rates and costs for mAb and maternal vaccine.

**Figure S7.** Percentage difference in cost per DALY averted in a middle-income country, relative to baseline cost of US$733 for mAb and US$929 for maternal vaccine, when each parameter is varied by +/-10%


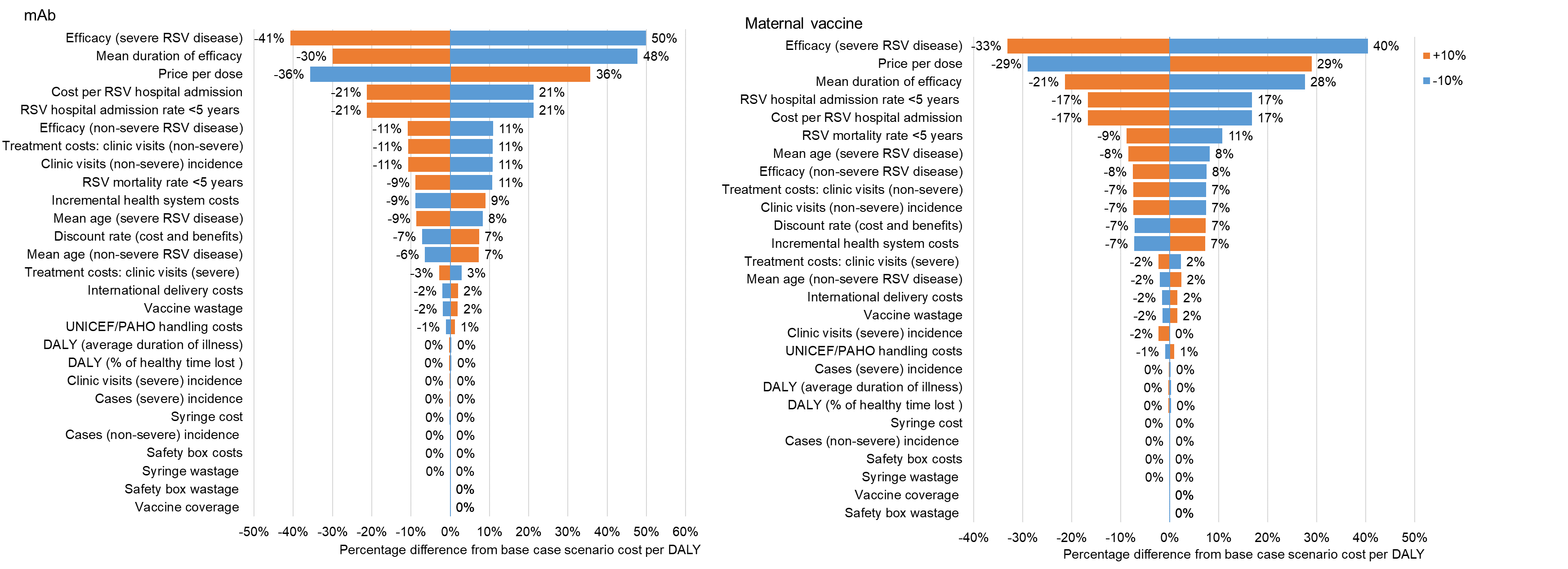


Caption: Cost per DALY averted was shown to be most sensitive to efficacy and duration of protection against severe RSV disease, price per dose, RSV hospital admission rates and costs.

**Comparison exercise**

All intermediate outcomes were similar with and without each intervention for the LSHTM (UNIVAC) and PATH models (Table S12 and S13).

**Table S12**. Comparison of ***maternal vaccine*** estimates by UNIVAC model and PATH model for the 2025 birth cohort in 133 LMICs

|  |  | **UNIVAC** | **PATH** | **Difference** |
| --- | --- | --- | --- | --- |
| Baseline | Cases | 31,882,631 | 31,729,439 | 0% |
|  | Visits | 20,797,633 | 20,123,119 | 3% |
|  | Admissions | 3,468,109 | 3,448,403 | 1% |
|  | Deaths | 108,904 | 107,535 | 1% |
|  | DALYs* | 3,116,915 | 3,131,302 | 0% |
|  | Program costs* | $0 | $0 | 0% |
|  | Treatment costs* | $2,499,520,164 | $2,449,440,258 | 2% |
|  |  |  |  |  |
| Vaccine | Cases | 27,723,308 | 27,638,264 | 0% |
|  | Visits | 18,032,036 | 17,494,389 | 3% |
|  | Admissions | 2,664,673 | 2,657,419 | 0% |
|  | Deaths | 87,358 | 86,501 | 1% |
|  | DALYs* | 2,491,362 | 2,516,652 | -1% |
|  | Program costs* | $524,999,363 | $520,888,197 | 1% |
|  | Treatment costs* | $2,038,255,391 | $2,007,666,977 | 2% |

Abbreviations: DALYs. Disability-adjusted life years; ICER, incremental cost-effectiveness ratio.

**Table S13**. Comparison of ***monoclonal antibody*** estimates by UNIVAC model and PATH model for the 2025 birth cohort in 133 LMICs

|  |  | **LSHTM** | **PATH** | **Difference** |
| --- | --- | --- | --- | --- |
| Baseline | Cases | 31,882,631 | 31,729,439 | 0% |
|  | Visits | 20,797,633 | 20,123,119 | 3% |
|  | Admissions | 3,468,109 | 3,448,403 | 1% |
|  | Deaths | 108,904 | 107,535 | 1% |
|  | DALYs* | 3,116,915 | 3,131,302 | 0% |
|  | Program costs* | $0 | $0 | 0% |
|  | Treatment costs* | $2,499,520,164 | $2,449,440,258 | 2% |
|  |  |  |  |  |
| mAb | Cases | 24,741,207 | 24,999,671 | -1% |
|  | Visits | 16,117,626 | 15,914,311 | 1% |
|  | Admissions | 2,229,278 | 2,280,680 | -2% |
|  | Deaths | 70,970 | 71,953 | -1% |
|  | DALYs* | 2,018,407 | 2,093,863 | -4% |
|  | Program costs* | $731,666,525 | $743,289,731 | -2% |
|  | Treatment costs* | $1,762,795,479 | $1,777,431,873 | -1% |

Abbreviations: DALYs. Disability-adjusted life years; ICER, incremental cost-effectiveness ratio; mAb, monoclonal antibody.

The proportion of countries willing to pay for infant mAB or maternal vaccine compared to no RSV intervention (status quo) was also very similar for both models (Figure S3).

**Figure S8. LSHTM (UNIVAC) and PATH model comparison of the percentage of 133 LMICs willing to pay for RSV intervention compared to status quo**

**
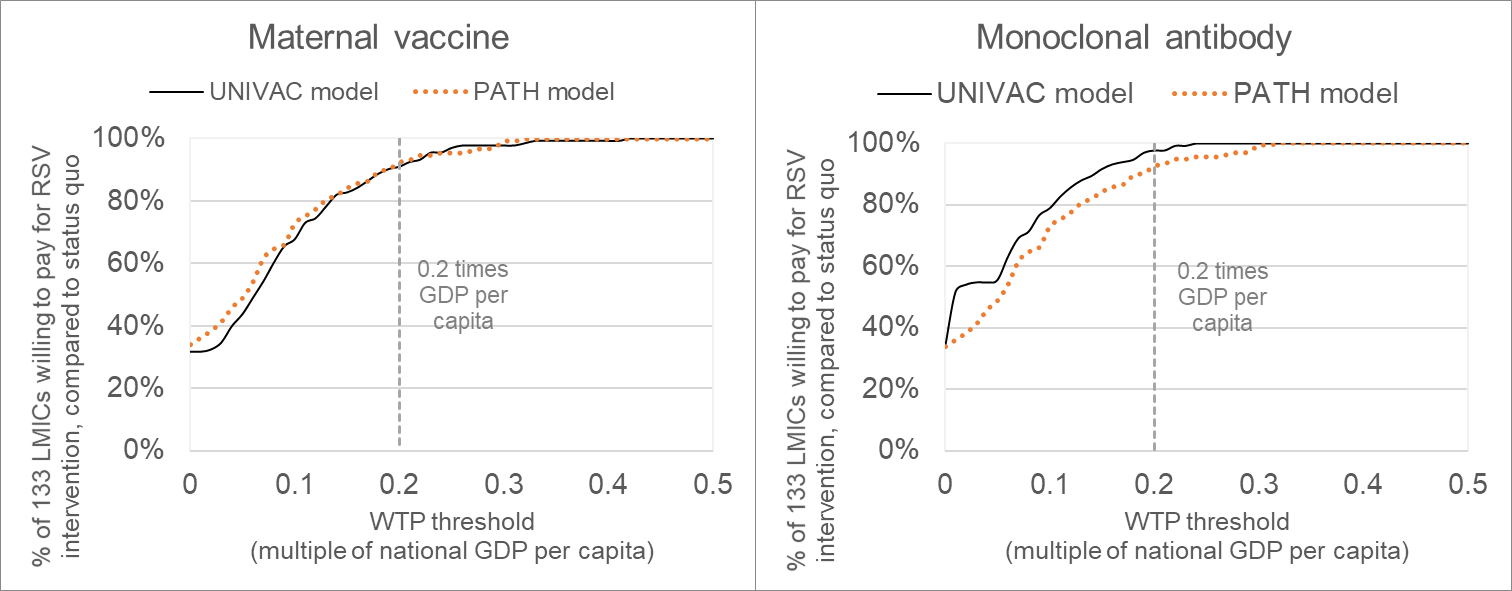
**

Caption: Both models predict a similar proportion of LMICs that would be willing to pay for each intervention at different WTP thresholds. In this comparison exercise the proportion of countries willing to pay for mAb is marginally higher than the proportion willing to pay for maternal vaccine. Both interventions are compared to status quo (rather than directly compared to each other) because the rank order (intervention with the most favourable cost-effectiveness) is very sensitive to subtle changes in the efficacy/waning assumptions used for both interventions. In addition in some populations it may only be feasible to use one of the available options to protect a specific group of infants. In these circumstances a comparison to status quo is appropriate. However, if it is feasible that either intervention could be used to protect the same infants (and better data emerges on the most uncertain inputs associated with each intervention i.e. efficacy, duration of protection, dose price) then a direct comparison would be appropriate.
